# Next generation sequencing under attack: investigating insider threats and organizational behaviour

**DOI:** 10.7717/peerj-cs.3008

**Published:** 2025-08-27

**Authors:** Nasreen Anjum, Hani Alshahrani, Darakhshan Syed, Asadullah Shaikh, Mahreen Ul Hassan

**Affiliations:** 1 Faculty of Technology, School of Computing, University of Portsmouth, Portsmouth, United Kingdom; 2 Department of Computer Science, Najran University, Najran, Saudi Arabia; 3 Emerging Technologies Research Lab (ETRL), College of Computer Science and Information Systems, Najran University, Najran, Saudi Arabia; 4 Department of Computer Science, Bahria University, Karachi, Pakistan; 5 Department of Information System, Najran University, Najran, Saudi Arabia; 6 Department of Microbiology, Shaheed Benazir Bhutto Women University, Peshawar, KPK, Pakistan

**Keywords:** Cybersecurity, Next generation sequencing, Human factors, Cybersecurity training, Cybersecurity awareness, Bio-cybersecurity, Privacy and security

## Abstract

Next generation sequencing (NGS) has become a cornerstone of modern genomics, enabling high-throughput analysis of DNA and RNA with wide applications across medicine, research, and biotechnology. However, the growing adoption of NGS technologies has introduced significant cyber-biosecurity risks, particularly those arising from insider threats and organizational shortcomings. While technical vulnerabilities have received attention, the human and behavioral dimensions of cybersecurity in NGS environments remain underexplored. This study investigates the role of human factors and organizational behavior in shaping cyber-biosecurity risks in NGS workflows. A mixed-method approach was employed, combining survey data from 120 participants across four countries with statistical analyses including chi-square tests, cross-tabulations, and cluster analysis. The study assessed cybersecurity training availability, employee engagement, training effectiveness, and awareness of insider threats. Findings reveal substantial gaps in training frequency and participation, with 36% of respondents reporting no access to NGS-specific cybersecurity training. Only a minority of participants felt confident in detecting cyber threats, and 32.5% had never applied cybersecurity knowledge in practice. Chi-square results indicate significant associations between training frequency and threat recognition, training relevance, and knowledge application. Cluster analysis further categorized organizations into “robust,” “moderate,” and “emergent” cybersecurity maturity profiles. The study offers an evidence-based framework to enhance cyber-biosecurity in NGS settings by addressing human-centric risks. It recommends role-specific training, frequent policy updates, and improved organizational communication to mitigate insider threats. These insights support the development of targeted interventions and policies to strengthen the cybersecurity culture in genomics organizations.

## Introduction

Next generation sequencing (NGS) is a groundbreaking technology that has transformed the field of genomics by enabling scientists to read and analyze DNA and RNA at an unprecedented scale and speed. Unlike traditional sequencing methods, such as Sanger sequencing, which can only process a single DNA fragment at a time, NGS allows for the simultaneous sequencing of millions of DNA fragments in parallel. This high-throughput capability has dramatically reduced the cost and time required for sequencing, making it accessible for a wide range of applications. For example, while sequencing the entire human genome once took years and cost billions of dollars, NGS can now accomplish the same task in a matter of days for just a few hundred dollars (Suminda et al., 2022).

This leap in technology has opened new frontiers in understanding the genetic foundations of diseases and has led to its application across a wide array of disciplines, including biomedical research, cancer genomics, personalized medicine, forensic science, agriculture, environmental monitoring, and more (Hu et al., 2021; Zhong et al., 2021; Kumar, Cowley & Davis, 2024). NGS technologies are being increasingly adopted not only in controlled laboratory settings but also in non-traditional environments such as crime scenes, patient homes, and even space missions (Albujja, 2024). It is even being used in environmental monitoring to study ecosystems and biodiversity, and in space missions to analyze the genetic material of organisms in extreme environments?.

Despite the many benefits of NGS, its rapid adoption introduces new challenges, particularly in the area of cyber-biosecurity. Cyber-biosecurity refers to the protection of biological data, systems, and infrastructure from cyber threats, ensuring the integrity, confidentiality, and availability of sensitive genomic information (Duncan et al., 2019; Murch et al., 2018). The sensitive nature of genomic data, which contains personal and heritable information, makes it a prime target for cyber threats. These risks are compounded by the growing accessibility of personal genomic data, with over 26 million people having taken at-home ancestry tests by 2019 and projections indicating that 60 million individuals globally will have their genomes sequenced by 2025 (Regalado, 2024; Phillips, Douglas & Marshall, 2020). This widespread use of NGS data raises significant concerns regarding its security, particularly given the growing awareness of cyber-biosecurity threats, including unauthorized access, data breaches, theft, and exploitation (Duncan et al., 2019).

### Problem statement

While technological advancements in NGS have revolutionized multiple fields, they have simultaneously introduced significant cyber-biosecurity risks. The sensitive nature of genomic data, with its direct link to personal identity, makes it particularly vulnerable to cyberattacks. Anjum et al. (2025) has identified numerous newly emerging risks to NGS systems including synthetic DNA malware attacks, data breaches, unauthorized access, and sequencing pipeline vulnerabilities that could compromise the integrity of genomic data. Among these, insider threats pose a particularly significant risk due to their potential to undermine security at various stages of the NGS workflow.

Insider threats in NGS environments can be intentional, involving deliberate acts of data manipulation and sabotage, or unintentional, arising from errors and negligence. These threats may manifest in multiple ways, such as unauthorized access to genomic databases, manipulation of sequencing results, or disruption of research activities. For instance, during DNA extraction, an insider could steal or contaminate biological samples, compromising the integrity of the sequencing process (Gymrek et al., 2013). Similarly, during library preparation, an attacker could introduce DNA-encoded malware, which, upon sequencing, could exploit vulnerabilities in computational systems. Additionally, personnel with privileged access to laboratory information management systems (LIMS) could misuse their credentials to exfiltrate or alter genomic data (Ney et al., 2017).

The scientific literature and industry surveys provide ample evidence of the importance and prevalence of this insider threat issue (Homoliak et al., 2019). Although external attacks are often the focus of cybersecurity efforts, it is equally vital to address the threats that originate within the organizational perimeter. According to the Global Report on the Insider threat Risks by Ponemon Institute and DTEX Systems (Gold, 2023), the average annual cost of insider-threat related risks has risen to $16.2 million, a 40% increase over the past four years. It now takes an average of 86 days to contain such incidents, and most insider threats (55%) are due to employee negligence or mistakes. The report also highlights that many organizations underfund their insider risk management efforts, dedicating only about 8.2% of their security budgets to insider threat mitigation, with most of that spent on post-incident responses rather than prevention. The Clearswift Insider Threat Index Report (Clearswift, 2022) from September 22, 2017, underscores this concern, revealing that 42% of IT security incidents result from employee actions, and 74% are linked to extended corporate activity. Since 2015, 40% of organizations have reported an increase in security incidents. These figures highlight the significant risk posed by insider threats and the urgent need to address them.

Despite advancements in cybersecurity, organizations managing NGS data remain vulnerable due to insufficient cybersecurity training, limited awareness, and a lack of employee engagement. Many personnel handling NGS data may not fully grasp the specific threats or the critical importance of secure practices. Furthermore, organizations often fail to implement regular and tailored cybersecurity training, leading to gaps in knowledge and increasing the risk of data breaches. This underscores the necessity of a thorough investigation into how human factors contribute to cybersecurity risks and what measures can be implemented to mitigate these vulnerabilities. Addressing these issues is essential for safeguarding sensitive genomic information and maintaining trust in NGS technologies.

### Aim of the study

The aim of the study is to explore the influence of insider threats (intentional/unintentional) on the cyber-biosecurity of NGS technology. Specifically, this study aims to investigate the level of cybersecurity awareness among the employees, quality and effectiveness of cybersecurity training programs, and the cybersecurity threats that arise due to the low engagement with cybersecurity practices. By identifying knowledge gaps and weaknesses in organizational approaches, the study will present insights and will offer guidelines for the development of more robust and effective policies and training programs.

### Objectives of the study

1. To investigate and analyse how human factors contribute to cyber-biosecurity risks in NGS data and workflow. This objective focuses on the role of insider threat and cybersecurity practices in the organization in shaping cybersecurity vulnerabilities.
2. To evaluate how frequently cybersecurity trainings are offered by the organizations and how actively employees participate or engage.
3. To examine how well cybersecurity programs improve employees’ ability to recognize and respond to threats and whether the training material addresses the needs of NGS data protection.
4. To assess the key factors that prevent effective cybersecurity education, specifically focusing on organizational shortcomings and employee participation gaps.
5. To examine the impact of cybersecurity incidents on organizational training and policies. This objective focuses on how real-world incidents have influenced the adaptation and improvement of cybersecurity measures within organizations.

### Research questions

1. How do human factors, including insider threats, contribute to cyber-biosecurity risks in NGS operations?
2. What is the current level of knowledge and awareness of cybersecurity among individuals involved in handling NGS data?
3. How frequently do organizations offer cybersecurity training programs tailored to the specific needs of NGS operations, and what is the level of employee engagement in these programs?
4. How confident are employees in detecting and responding to cybersecurity threats specific to NGS data, and how relevant are the training programs to their day-to-day tasks?
5. What are the primary barriers faced by organizations in implementing effective cybersecurity training for NGS data protection, and how do past incidents shape organizational responses to improving cybersecurity practices?

### Hypotheses

Building on the objectives and literature review, we propose the following testable hypotheses:

1. **H1:** “Organizations with robust cybersecurity practices will exhibit significantly fewer insider threats, higher employee confidence in threat detection (Q7), and more frequent incident-driven policy updates (Q12) compared to moderate/emergent clusters.”
2. **H2:** “Organizations with moderate cybersecurity practices will show intermediate levels of threat awareness (Q17–Q20) and inconsistent policy enforcement, with reliance on reactive measures post-incident.”
3. **H3:** “Organizations with emergent cybersecurity practices will report the lowest employee confidence (Q7), poorest threat detection (Q10), and minimal training engagement (Q5–Q6), correlating with higher insider risks.”

### Organization of study

This study is organized as follows: ‘Literature Review’ reviews relevant literature on insider threats, organizational behavior, and training effectiveness, identifying gaps that this study aims to address. ‘Research Methodology’ describes the research methodology, including data collection, participant selection, ethical considerations, and statistical techniques used for analysis. ‘Results and Discussion’ presents and discusses the results, highlighting key findings related to training frequency, awareness, and organizational practices. Finally, ‘Conclusion, Recommendations and Future Work’ concludes the study with a summary of findings and practical recommendations for enhancing cybersecurity in NGS workflows.

## Literature review

The intersection of cybersecurity and biological data, often referred to as cyber-biosecurity, has gained increasing attention due to the sensitive nature of genomic data and its implications for privacy, security, and ethical concerns. Foundational work in this area has established the importance of securing biological data from both external and internal threats. For instance, Duncan et al. (2019) introduced the concept of cyber-biosecurity, emphasizing the need to protect biological data and systems from cyber threats, particularly in the context of agriculture and healthcare. Their work laid the groundwork for understanding the unique vulnerabilities of biological data, including genomic information, and the potential consequences of breaches.

Murch et al. (2018) further expanded on the definition of cyber-biosecurity, describing it as the “convergence of cybersecurity and biosecurity to protect the bioeconomy.” They highlighted the dual nature of threats, where cyberattacks can compromise biological systems, and biological data can be exploited for malicious purposes. This dual focus underscores the need for integrated security measures that address both cyber and biological risks.

Similarly, Peccoud et al. (2018) provided a comprehensive framework for understanding cyber-biosecurity, defining it as “the protection of biological data, processes, and systems from cyber threats.” Their work emphasized the importance of securing the entire lifecycle of biological data, from collection and storage to analysis and sharing, and called for interdisciplinary approaches to address the complex challenges at the intersection of biology and cybersecurity.

In addition, Ney (2019) explored the emerging threats to bioinformatics systems, particularly those involving NGS technologies. Ney defined cyber-biosecurity as “the safeguarding of biological information systems from unauthorized access, manipulation, or theft,” and highlighted the critical role of insider threats in compromising genomic data. This work provided early insights into the specific risks associated with NGS technologies and the need for tailored cybersecurity measures.

### Insider threats in biological sciences and DNA-related data

While the foundational literature establishes the importance of cyber-biosecurity, there is a growing body of work focusing specifically on insider threats in biological sciences and DNA-related data. Insider threats, whether intentional or accidental, pose unique challenges due to the sensitive nature of genomic data and the potential for misuse. Below, we critically analyze five key studies that address insider threats in this domain:

Arshad et al. (2021) investigated security and privacy challenges in DNA-genomics applications, identifying insider threats as a significant risk factor. The authors emphasized that insiders with authorized access, whether through malicious intent or accidental actions, can compromise sensitive genomic data, leading to breaches and data misuse. While their study highlights the need for enhanced access controls and employee training, it does not explore the organizational and behavioral factors that contribute to insider threats in NGS environments.

Bhushan (2023) explored the growing threats to genomic data, with a focus on both insider and external vulnerabilities. The authors highlighted insider threats where authorized personnel either misuse or inadvertently expose sensitive genomic data. However, their work primarily focuses on technical solutions, such as encryption and access control, without addressing the human factors that drive insider threats, such as lack of training or organizational culture.

Alsaffar et al. (2022) analyzed the security and privacy challenges associated with the digital DNA lifecycle. Their work highlighted vulnerabilities at various stages of the DNA lifecycle, from sequencing to storage and sharing, emphasizing that insider threats, such as unauthorized access by lab technicians or information technology (IT) personnel, pose significant risks. While this study provides a comprehensive overview of vulnerabilities, it does not offer actionable insights into how organizations can mitigate insider threats through training and policy changes.

Burrell et al. (2022) identified insider threats—whether malicious, negligent, or accidental—as a critical cybersecurity risk in the biotechnology and healthcare sectors. The authors underscored the importance of robust monitoring systems and the enhancement of cybersecurity training to mitigate these threats. However, their study lacks a detailed analysis of how organizational behavior and employee engagement influence the effectiveness of such training programs.

Millett, Dos Santos & Millett (2019) highlighted insider risks in the biotech sector, emphasizing unauthorized access and data manipulation as major concerns. Their study stressed the need for stronger security measures and greater awareness among employees. While this work provides valuable insights into the risks posed by insiders, it does not address the specific challenges of NGS technologies or the role of organizational culture in mitigating insider threats.

### Critical analysis and research gaps

The reviewed literature highlights significant concerns surrounding insider threats to genomic data, particularly in the context of NGS technologies. However, several critical gaps remain:

**Lack of focus on organizational behavior:** While many studies emphasize the technical aspects of cybersecurity, such as encryption and access control, there is limited research on how organizational behavior and culture influence insider threats. For example, Arshad et al. (2021) and Bhushan (2023) focus on technical solutions but do not explore how organizational policies and employee engagement can mitigate risks.

**Insufficient attention to training effectiveness:** Although several studies, such as Burrell et al. (2022) and Millett, Dos Santos & Millett (2019), highlight the importance of cybersecurity training, they do not critically evaluate the effectiveness of such training programs. There is a lack of research on how training can be tailored to address the specific challenges of NGS technologies and how it can be integrated into organizational culture.

**Limited exploration of human factors:** The role of human factors, such as employee awareness, confidence, and engagement, in mitigating insider threats is underexplored. For instance, Alsaffar et al. (2022) and Ney (2019) discuss the risks posed by insiders but do not provide a detailed analysis of how human factors contribute to these risks.

**Gaps in NGS-specific research:** While Ney (2019) and Peccoud et al. (2018) provide foundational insights into the risks associated with NGS technologies, there is a lack of research on how organizations can design policies and training programs specifically tailored to NGS environments.

### Contribution of this study

This study addresses these security gaps by examining how human behavior and organizational culture drive insider threats in NGS settings. In contrast to previous studies, which primarily focus on technical solutions, our research investigates the effectiveness of cybersecurity training programs, the role of employee engagement, and the impact of organizational culture on mitigating insider threats. By analyzing data from 120 participants across diverse roles and expertise levels, we provide actionable insights into how organizations can design tailored training programs and foster a proactive cybersecurity culture. Our work contributes to the field by:

- **Systematization of human-centric risks in NGS cybersecurity:** Identifies and categorizes organizational behavior, insider threats, and training gaps unique to NGS workflows, addressing the literature’s underemphasis on human factors in genomic data security. Provides the first empirical mapping of threat vectors across the NGS pipeline (Table 1) and establishes testable hypotheses (H1–H3) linking organizational practices to insider risks.
- **Evidence-based training optimization model:** Demonstrates statistically (*via* chi-square tests) that role-specific training frequency correlates with improved threat detection (Q7) and knowledge application (Q9). Offers actionable thresholds for training design (*e.g*., quarterly sessions show 23% higher confidence *vs*. annual).
- **Organizational maturity typology:** Proposes a data-driven classification of NGS organizations into robust, moderate, and emergent cybersecurity profiles using cluster analysis, revealing that “robust” organizations combine frequent training (Q5), proactive policies (Q13), and cross-departmental communication (Q14).
- **Bio-cybersecurity awareness benchmark:** Establishes baseline metrics for NGS-specific cybersecurity awareness, exposing critical gaps: 34% of users are unfamiliar with common threats (Q19), and 22% unaware of “Bio-Cyber Security” (Q20). These metrics enable targeted interventions.
- **Policy levers for incident response:** Identifies underutilized post-incident policy updates (only 24% of organizations revise training after breaches, Q12) and advocates for structured drills, leadership engagement, and dedicated cybersecurity liaisons.

**Table 1:** Results of descriptive statistics for demographic information.

Statistics	Q1	Q2	Q3	Q4
Mean	63.58	26.19	3.61	42.89
Median	67.00	31.00	2.00	39.00
Std	10.90	14.38	9.19	16.59

By addressing these gaps, our study not only builds upon previous research but also provides a comprehensive framework for enhancing cybersecurity in NGS environments.

## Research methodology

A well structured methodology is used to investigate the current state of cyber-biosecurity protocols, understanding, and training among organisations handling sensitive NGS data. The methodology’s objective is to methodically collect and analyse data in order to draw significant insights on the effectiveness of cyber-biosecurity awareness initiatives, individual’s awareness levels, and these organisations’ overall security culture. This section describes the collection of data, and analysis of collected responses using various analytical methods.

The survey questions have been carefully designed to collect multiple aspects of cybersecurity awareness and training, particularly for organizations dealing with NGS data-handling. The questions were categorized during the design process into seven important parts in order to fully address the various aspects of cybersecurity concerns and behaviors. The demographic data, training frequency and availability, training effectiveness and relevance, identifying the barriers and challenges, incident-driven modifications, organizations’ behavior and security culture, and user awareness and understanding of cybersecurity were the categories into which the questions are classified. To ensure that the collected data would offer a comprehensive picture of the cybersecurity landscape within NGS environments, each part was specifically intended to address a particular aspect of cybersecurity related to NGS environment.

### Data collection

The dataset we collected comprises responses from five different organizations utilizing NGS technology, either fully or partially, or dealing with sensitive DNA data. The data includes responses from individuals across four countries: Pakistan, South Korea, the United Kingdom, and Thailand, with a total of 120 participants. These individuals represent a diverse cross-section of NGS users, including professionals, researchers, teachers, IT/cybersecurity experts, and students. We designed 20 questionnaires for data collection. The objective is to gather insights in cyber-biosecurity awareness and investigate the risks linked with insider threats, organizational behavious and culture in dealing with cybersecurity risks, and engagement of employes with security practices and training. To ensure the comprehensive view of human factors affecting NGS data security, demographic information was gathered including role, age, experience level, and frequency of handling and managing of sensitive data. By targeting a wide range of NGS users and roles, the dataset provides a holistic perspective on how different organizations manage sensitive genomic data.

### Data cleaning methodology

To ensure unbiased and reliable results, we implemented a rigorous data cleaning protocol to maintain data integrity and relevance for our study on cybersecurity awareness in NGS environments. The process involved:

1. **Duplicate removal:** Eliminating redundant responses to prevent skewed analysis.
2. **Missing data imputation:** Replaced missing values with the median.
3. **Categorical variables:** Filled gaps with “Unknown/Not Sure” where applicable.
4. **Relevance filtering:** Applied inclusion criteria to exclude respondents outside the NGS domain or those not handling sensitive data.

### Ethical considerations

This study employed a structured, anonymized questionnaire distributed *via* a secure online platform (Google Forms). Ethical approval was obtained prior to data collection, and informed consent was explicitly acquired from all participants, clearly explaining the study’s objectives, voluntary participation, confidentiality assurances, and the right to withdraw at any stage. To maintain confidentiality and protect privacy, all personally identifiable information, including participant names, email addresses, and specific organizational identifiers, was removed from the dataset before analysis. Data were securely stored on encrypted servers accessible only to authorized research personnel. Although the dataset generated during this study is available as open-access material for replication or further research purposes, personal identifiers and organizational names have been permanently removed to comply with ethical and privacy constraints.

### Participant selection and sampling procedure

Participants for this study were purposefully selected to represent diverse roles and levels of experience within organizations managing NGS data in healthcare contexts. A purposive sampling strategy was used to ensure inclusion of professionals directly engaged with cybersecurity and NGS operations, such as healthcare practitioners, bioinformaticians, IT/cybersecurity specialists, and research personnel, from organizations actively involved in NGS workflows.

Organizations were identified based on their active use or management of sensitive genomic data through NGS technologies. Invitations to participate were distributed across professional networks and specialized forums to enhance representation across various roles and geographic locations. While efforts were made to mitigate selection bias by including diverse professional roles and organizational contexts, it is acknowledged that voluntary participation may introduce potential bias. To reduce this limitation, anonymization of responses, clear explanation of study objectives, and the assurance of confidentiality were emphasized to encourage honest and unbiased responses from participants.

### Statistical techniques

Many statistical techniques are selected to offer a strong basis for analysing the complex relationships that exist between individual behaviours, organisational procedures, and demographic characteristics in the context of cybersecurity in NGS contexts.

#### Descriptive statistics

To summarize the data’s variability and central patterns, descriptive statistics were used (George & Mallery, 2018). This method provides a general overview of the respondents’ demographic data, the availability and frequency of cybersecurity training, the effectiveness and practicality of cybersecurity trainings, and the level of user understanding and awareness of cybersecurity issues specific to NGS technology. Descriptive statistics, which compute the mean, median, and standard deviation, made it possible to comprehend the distribution and main patterns in the responses, assisting in the identification of important trends and areas for improvement.

#### Frequency analysis

By measuring the frequency of replies, frequency analysis provides information about the most frequent routines, behaviours, and awareness levels among the respondents (Freund & Wilson, 2003). This technique is especially helpful for emphasising the frequency of specific behaviours or knowledge levels, such as the frequency with which organisations that offer cybersecurity training or the frequency with which users engage in such initiatives. Frequency analysis is also useful for finding gaps or areas that require improvement, that include the percentage of people who participate in cybersecurity training.

#### Cross-tabulation

To investigate the correlations between various variables, including the role or experience level of respondents and their involvement in cybersecurity training the cross-tabulation method is used. By identifying patterns and correlations between variables, this approach made it possible to understand how various groups within an organisation interact with cybersecurity policies. When it comes to finding variations among roles or experience levels, cross-tabulation is very useful since it can help guide specific alternatives (Momeni et al., 2018).

#### Chi-square test

The chi-square test is used to find out if there were any significant correlations between categorical variables like the one between cybersecurity training and effectiveness of training. To determine whether organisations offering cybersecurity training are effectively successful and whether users understand the necessary knowledge to recognise or perceive any cybersecurity related threat. Furthermore, this approach supports the assessment of hypotheses concerning organizational procedures and internal vulnerabilities since it is especially suitable for investigating the independence of categorical data (Rana & Singhal, 2015).

#### Cluster analysis

Organisations are grouped using clustering based on their cybersecurity practices and cultures. By spotting unique patterns or clusters in the data, this technique is useful for classifying organisations based on how they handle cybersecurity. Cluster analysis showed how various organisations align in terms of cybersecurity behaviours, communication methods, and overall culture through analysing the responses to relevant questions. This method works especially well for showing differences in cybersecurity maturity between various organisations. This approach corresponded closely to the investigation’s hypotheses by offering a data-driven classification into “robust,” “moderate,” and “emergent” profiles. Finding common patterns and investigating the relationship between managerial competence and cybersecurity outcomes, including decrease in insider threats, are made possible by the clustering technique (Scitovski et al., 2021).

## Results and Discussion

The results of this study are divided into seven key sections. In ‘Demographic information’, the statistical methods used to analyze the demographic information of respondents are presented. These methods include descriptive statistics and frequency analysis, which provide insights into the respondents’ age group, organizational role, level of experience, and the frequency with which they handle sensitive NGS data.

‘Frequency and availability of training’ details the statistical approaches employed to assess the frequency and availability of cybersecurity training within NGS environments. Descriptive statistics, frequency analysis, and cross-tabulation methods are utilized to evaluate the regularity and accessibility of cybersecurity training sessions in organizations managing NGS data.

‘Effectiveness and relevance of training’ focuses on the analysis of the effectiveness and relevance of cybersecurity training in NGS environments. This section employs descriptive statistics, frequency analysis, and chi-square tests to measure how effective and pertinent these training programs are for organizations processing NGS data.

In ‘Barriers and challenges’, the barriers and challenges related to cybersecurity training in NGS environments are analyzed. Descriptive statistics and frequency analysis are applied to identify the key obstacles organizations face when implementing cybersecurity training programs.

‘Incident-driven modifications’ examines the modifications made to cybersecurity training programs in response to actual incidents. Descriptive statistics and frequency analysis are used to explore how organizations adapt their cybersecurity policies and training in response to cybersecurity threats or breaches.

‘Organization behavior and security culture’ presents the methods used to analyze organizational behavior and security culture regarding cybersecurity training in NGS environments. Descriptive statistics, frequency analysis, and cluster analysis are employed to assess the overall cybersecurity culture, including communication methods, security policies, and organizational attitudes toward cybersecurity.

Finally, ‘User knowledge and awareness of cybersecurity’ investigates user knowledge and awareness of cybersecurity within NGS environments. Descriptive statistics and frequency analysis are utilized to evaluate the extent to which users are aware of cybersecurity concerns specific to NGS technology and their adherence to best practices.

### Demographic information

Fundamental background information is gathered in the demographic section, such as the respondent’s age group, role within the company, level of experience, and frequency of handling sensitive NGS. This data makes the responses more relevant and enables research of cybersecurity awareness and behaviour based on the provided demographics. Following questions are considered for this section:

**Q1: **Please indicate your role within the organization:

**Q2:** How long have you been working in the NGS?

**Q3:** What is your age group?

**Q4:** How often do you handle sensitive NGS data that could be targeted in a cybersecurity attack?

#### Descriptive statistics

**Figure 1:**
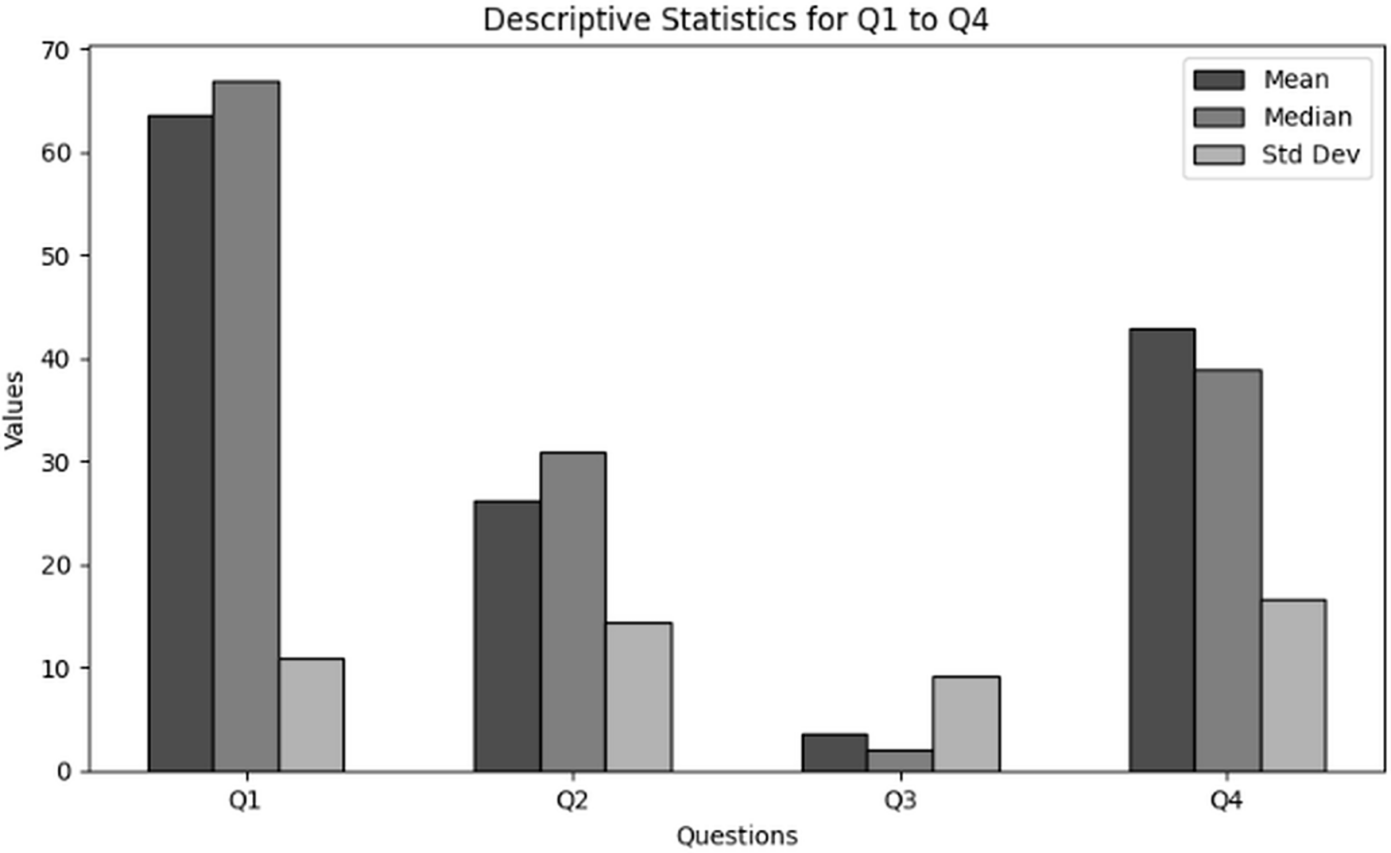
The graphical visualization of descriptive statistics of demographic information in cybersecurity dealing with NGS data through Q1–Q4.

The purpose of descriptive statistics is to summarize the data to obtain comprehensive insights into demographic information of users in cybersecurity working with NGS data. This includes calculating means, medians, and standard deviations as shown in Table 1. For every question (Q1 through Q4), the mean, median, and standard deviation are included in the table to provide an overview of the responses’ primary patterns and variations. Figure 1 presents the graphical visualization of the descriptive statistics of Q1–Q4 to summarize the values of mean, median and standard deviations.

**Key observations drawn from the results of descriptive statistics:** The conclusions drawn from these descriptive statistics to analyze the demographic information of users in cybersecurity working in NGS environment is presented below. The findings present significant insights:

**Role within the organization (Q1):** The majority of the respondents based on role are classified as students, which indicate the major involvement of academia. Furthermore, moderate portion on respondents are IT or cybersecurity specialists. However, the results indicate that there is no role based variety of respondents which indicates NGS as a new field of interest.

**Level of experience (Q2):** The results obtained clearly indicate that most of the respondents are unexperienced with NGS. This indicates that a considerable portion of respondents are new to the field.

**Age group (Q3):** Most of the respondents are skewed towards 18–24 years age group. Although there are outliers in the upper age groupings, most users are in the lower age groups.

**Handling sensitive NGS data (Q4):** A significant number of respondents “rarely” handled sensitive NGS data. However, on average, the respondents deal with NGS data regularly. The overall results showed a varying degree of responders’ interaction with NGS. However, most are at the learning stage, *i.e*., students.

#### Frequency analysis

**Figure 2:**
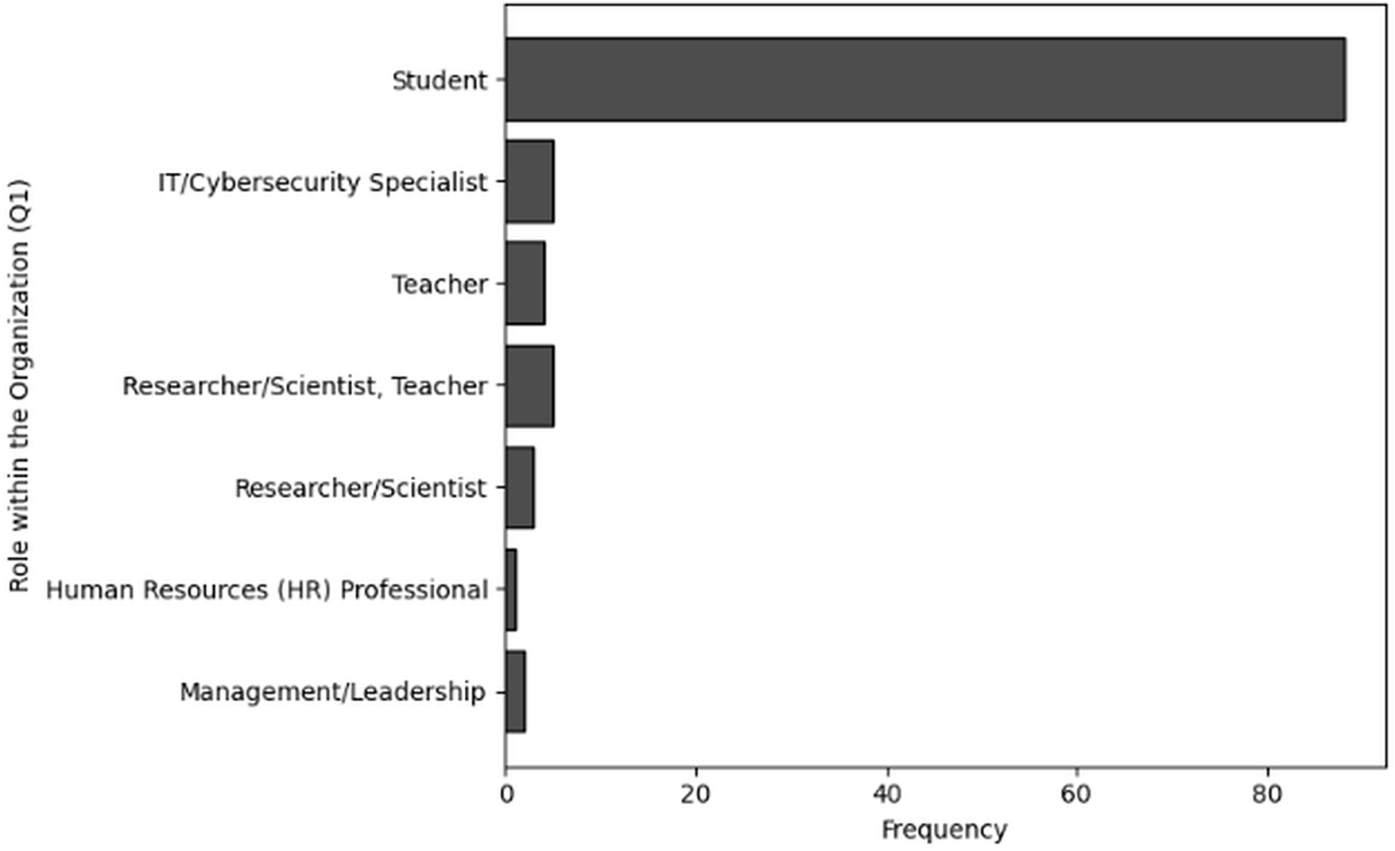
The graphical visualization of frequency analysis of role within the organization (Q1).

**Figure 3:**
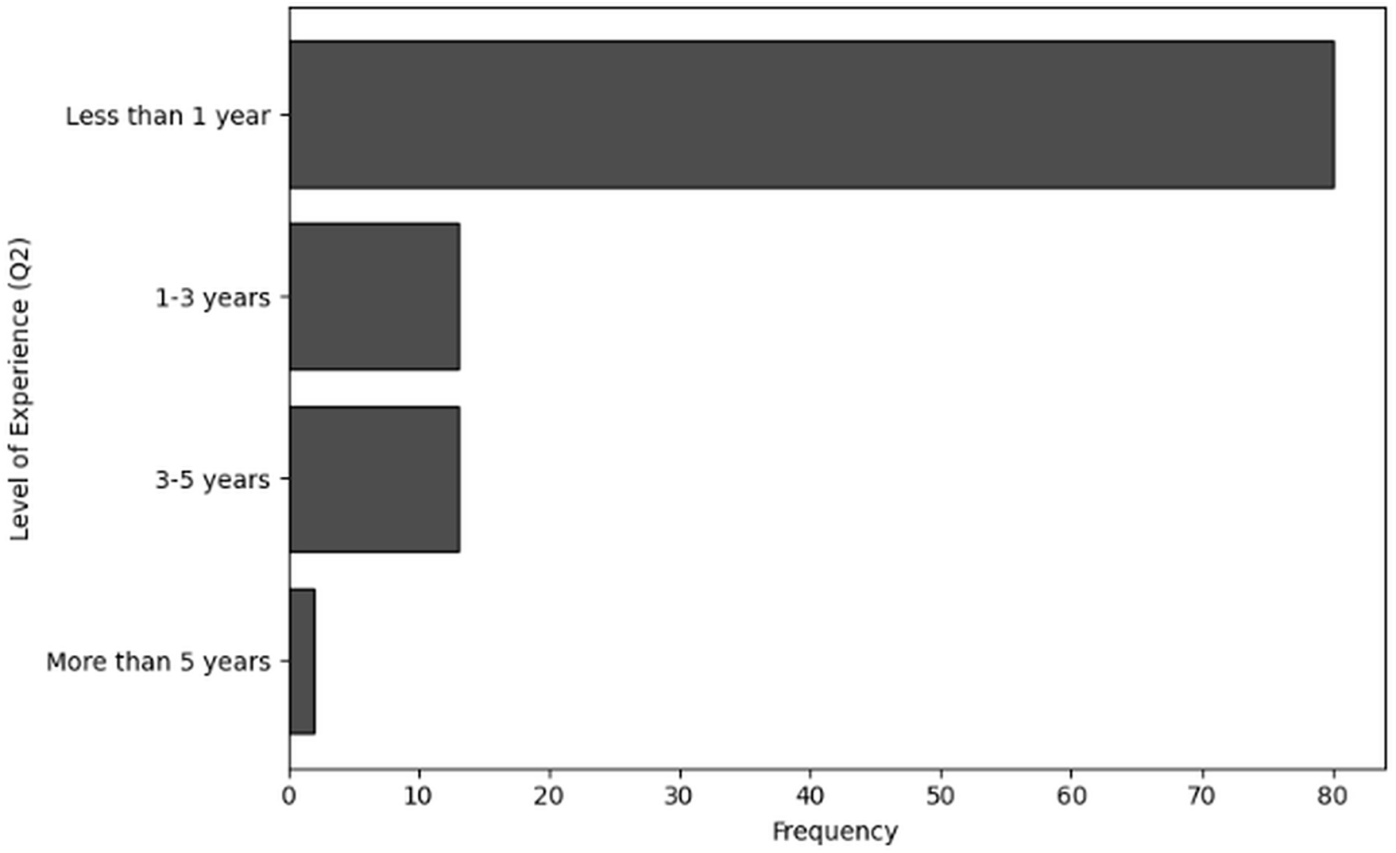
The graphical visualization of frequency analysis of level of experience (Q2).

**Figure 4:**
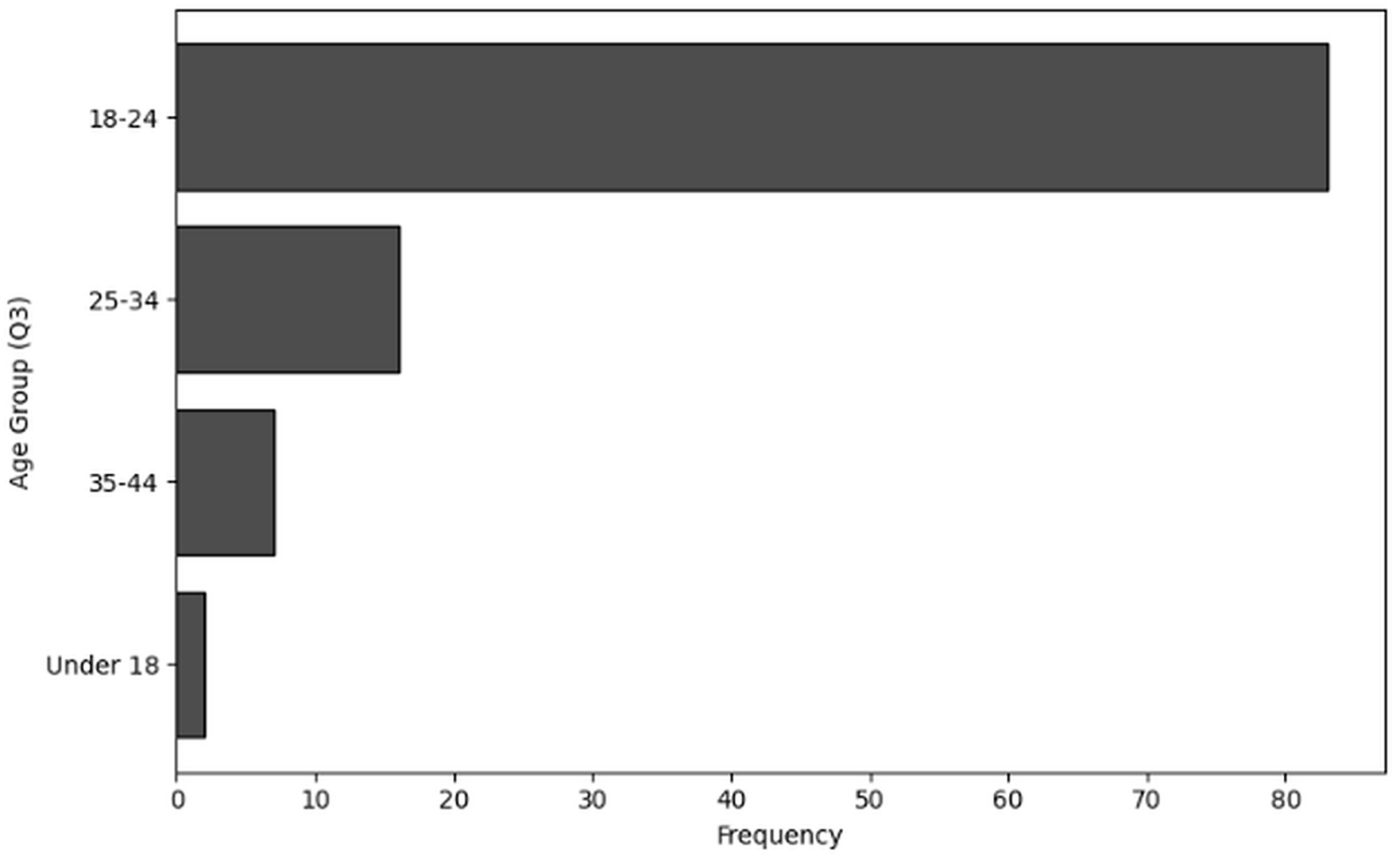
The graphical visualization of frequency analysis of age group (Q3).

**Figure 5:**
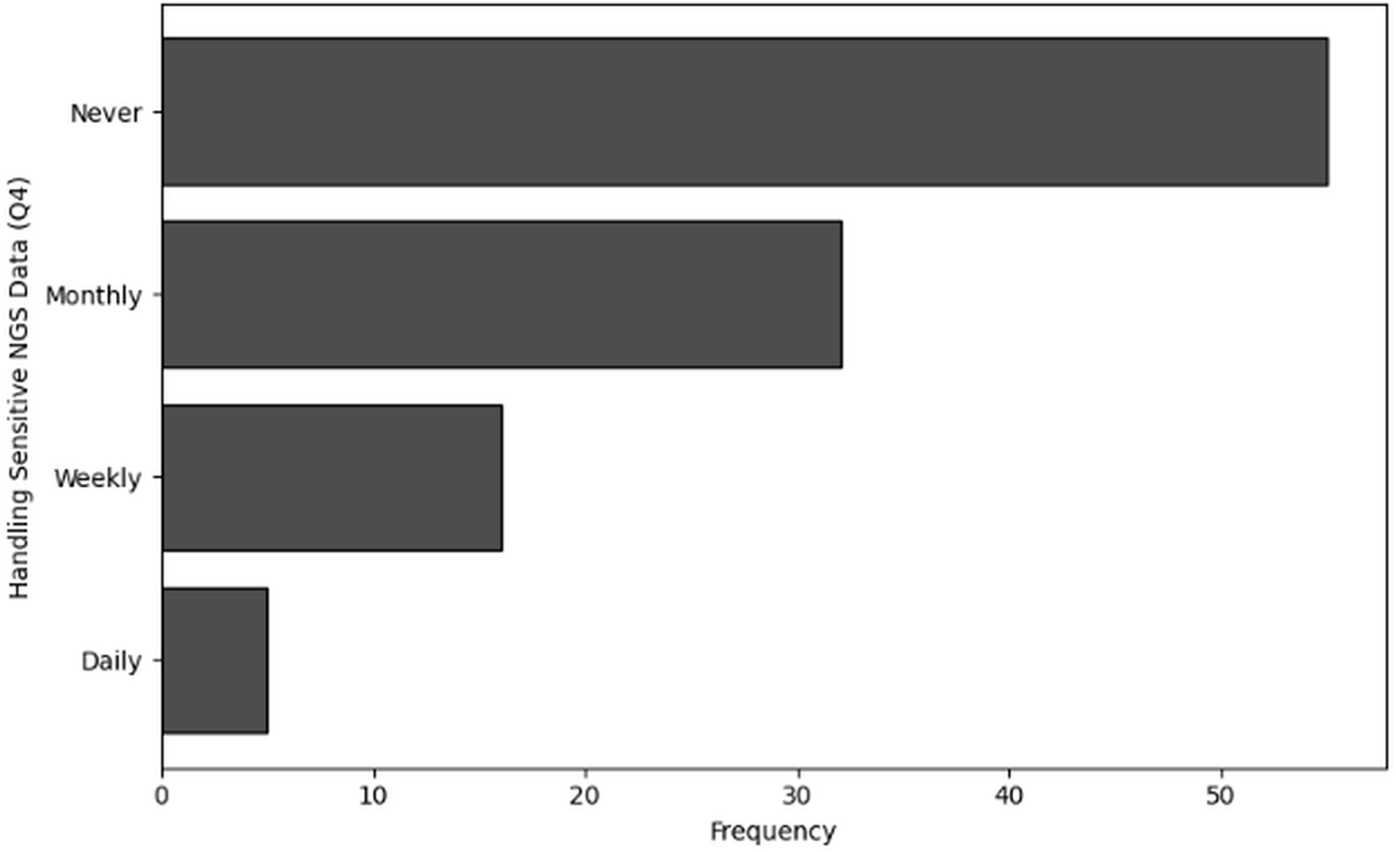
The graphical visualization of frequency analysis of handling sensitive NGS data (Q4).

The purpose of frequency analysis is to summarize the respondents replies to count the responses for all the categories to determine frequent behaviors in terms of demographic information of users working in NGS by considering relevant questions (Q1–Q4). The results of responses to analyze the frequency of user inputs for of role within the organization (Q1), level of experience (Q2), age group (Q3) and handling sensitive NGS data (Q4) are shown in Tables 2, 3, 4 and 5, respectively. The visual representations of frequency analysis of Q1, Q2, Q3 and Q4 are shown in Figs. 2, 3, 4 and 5, respectively.

**Table 2:** Results of frequency analysis of role within the organization (Q1).

Role within the organization (Q1)	Frequency
Student	88
IT/Cybersecurity specialist	5
Teacher	4
Researcher/Scientist, Teacher	5
Researcher/Scientist	3
Human resources (HR) professional	1
Management/Leadership	2

**Table 3:** Results of frequency analysis of level of experience (Q2).

Level of experience (Q2)	Frequency
Less than 1 year	80
1–3 years	13
3–5 years	13
More than 5 years	2

**Table 4:** Results of frequency analysis of age group (Q3).

Age group (Q3)	Frequency
Under 18	2
18–24	83
25–34	16
35–44	7

**Table 5:** Results of frequency analysis of handling sensitive NGS data (Q4).

Handling sensitive NGS data (Q4)	Frequency
Never	55
Monthly	32
Weekly	16
Daily	5

**Key observations drawn from the results of frequency analysis:** The conclusions drawn from frequency analysis to analyze the demographic information of users in cybersecurity working in NGS environment are presented below. The findings present significant insights:

**Role within the organization (Q1):** The most common category of respondents (*i.e*., 88) identified as students, representing most users. This suggests an active representation of individuals who are probably working in academic environments and engaged in NGS-related research. IT/Cybersecurity professionals make up a lower portion of the responders (*i.e*., 5), which is significant because these positions are usually directly responsible for administering and preserving cybersecurity procedures. Furthermore, Teachers are only 3, the number of responders who are researcher/scientist and teacher as well are 5. While the frequency count of responders who are purely Researcher/Scientist is 3. In addition to performing research related to NGS, which involve cybersecurity considerations, teachers and researchers/scientists play a crucial role in sharing knowledge. Furthermore, the frequency of HR and management professionals indicate that individuals holding leadership or HR positions are not aware of cybersecurity related to NGS. Even though their presence adds value by offering insights from various organisational opinions.

**Level of experience (Q2):** Most users have less than a year’s experience with NGS, suggesting that most users are still relatively new to this field. Lesser percentages of users were using the service for 1–3 years (13 responses), these users still be developing their cybersecurity skills, but they are probably more familiarised with NGS procedures. The group of respondents that have 3–5 years (13 responses) of experience are more matured and have a better awareness of NGS and related cybersecurity threats. Additionally, the group with the least experience is over more than 5 years (two responses). These responders most likely have extensive understanding of NGS operations and the cybersecurity difficulties they encounter, making them experts in their organization.

**Age group (Q3):** The majority of responders’ age group is 18–24 years old 83 respondents. Typically, individuals in this age range are just starting their professions and are still learning about cybersecurity and NGS. The age ranges of 25–34 (16) and 35–44 (7) indicate those who are more likely to be well-established in their current positions and have senior or specialised roles in their companies. Additionally, the frequency of under 18 (2) users suggests that few participants are in the very early phases of their training or involvement in NGS-related activities considering the presence of responders under 18. Here it can be concluded that a greater number of younger users participate in NGS activities.

**Handling sensitive NGS data (Q4):** A substantial number of respondents (55) said they “never” handle sensitive NGS data. This represent the roles played by students and other participants in the field who are not directly handling sensitive NGS data in practice. Some responders handle sensitive data “monthly” (32), indicating occasional involvement in high-security duties. Furthermore, fewer people handle sensitive data “weekly” (16), suggesting a greater degree of engagement in tasks for which cybersecurity is essential. Few respondents “daily” (5) handle sensitive NGS data. These people most likely work in positions where they regularly interact with data that requires strict cybersecurity protocols.

#### Overall conclusion of demographic information

According to the demographic study, the responder population is generally made up of younger people, mainly students between the ages of 18 and 24, who are relatively new to the NGS field; the majority having less than a year of experience. This group is not likely to handle sensitive NGS data on a regular basis; in particular, a significant portion never interacts with it. Even though their number is smaller, the existence of IT/cybersecurity specialists emphasises the importance of important positions that are specifically in charge of cybersecurity across organisations. However, the aggregate data indicates that a considerable proportion of participants can benefit from focused cybersecurity experience and training, particularly considering their limited background and infrequent handling of confidential data. These findings highlight the necessity of customised approaches to improve cybersecurity knowledge and behaviours among the emerging NGS users.

### Frequency and availability of training

This section evaluates how regularly, and easily accessible cybersecurity training sessions are in organisations that handle NGS data. The main goal is to ascertain the frequency of both the trainings’ offering and employee participation. The section seeks to determine the following by assessing these factors:

**Frequency of cybersecurity training:** The degree to which the company is committed to providing its staff with regular cybersecurity training and education, particularly regarding NGS operations. Regular training sessions show that upholding strong cybersecurity standards is being done in a proactive manner.

**Frequency of participation in cybersecurity training:** The extent to which employees take part in these training initiatives. High participation percentages imply that staff members are involved in ongoing training to safeguard critical NGS data and are conscious of the significance of cybersecurity.

Following questions are considered for this section:

**Q5:** How often does your organization provide cybersecurity training specifically tailored to NGS operations?

**Q6:** How often do you participate in cybersecurity training or awareness programs specifically tailored for NGS data security?

#### Descriptive statistics

**Figure 6:**
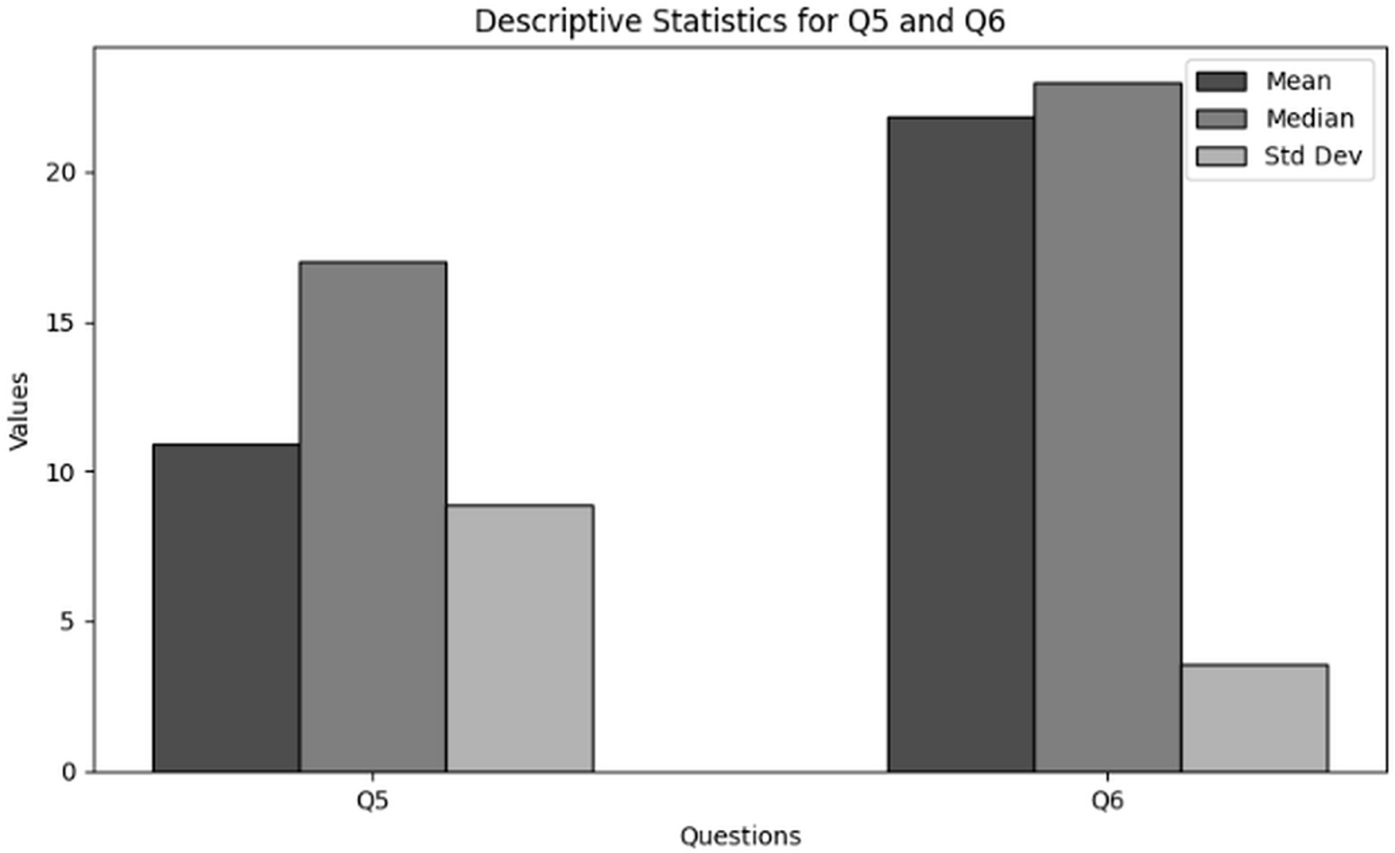
The graphical visualization of descriptive statistics of frequency and availability of training in cybersecurity dealing with NGS data through Q5 and Q6.

The purpose of descriptive statistics is to summarize the data to obtain comprehensive insights into frequency and availability of training of users in cybersecurity working with NGS data. This includes calculating means, medians, and standard deviations as shown in Table 6 respectively. For every question (Q5 and Q6), the mean, median, and standard deviation are included in the table to provide an overview of the responses’ primary patterns and variations. Figure 6 presents the graphical visualization of the descriptive statistics of Q5 and Q6 to summarize the values of mean, median and standard deviations.

**Table 6:** Results of descriptive statistics for frequency and availability of training of users in cybersecurity dealing with NGS data through Q5 and Q6.

Statistics	Q5	Q6
Mean	10.93	21.83
Median	17.00	23.00
Std	8.86	3.56

**Key observations drawn from the results of descriptive statistics:** The conclusions drawn from descriptive statistics to analyze the Frequency and Availability of Training in cybersecurity dealing with NGS data through Q5 and Q6 is presented below. The findings present significant insights:

**Frequency of cybersecurity training (Q5):** The statistical results obtained in terms of the frequency of cybersecurity trainings specific to NGS conclude that the average frequency of trainings conducted in various organizations working in NGS environment is moderately low. However, some organizations offer trainings on regular basis while mostly do not offer any training sessions.

**Frequency of participation in cybersecurity training (Q6):** The statistical results obtained in terms of the organization’s participation in cybersecurity training and awareness programs specifically tailored for NGS data security concludes that participation in cybersecurity training for NGS data security is comparatively high in some organizations. Although, some respondents do not participate at all which indicates a major required of cybersecurity education related to NGS.

#### Frequency analysis

The purpose of frequency analysis is to summarize the identification of common activities and behaviors in terms of frequency and availability of Training in cybersecurity dealing with NGS data by counting the number of inputs for each category. The results of the frequency analysis for training availability and frequency, based on questions Q5 and Q6, reveal some important insights. For Q5, which inquired about the frequency of training, a significant portion of respondents, 44 in total, indicated that they had never received training. This was followed by 24 respondents who mentioned receiving training annually, while 19 respondents reported bi-annual training.

**Figure 7:**
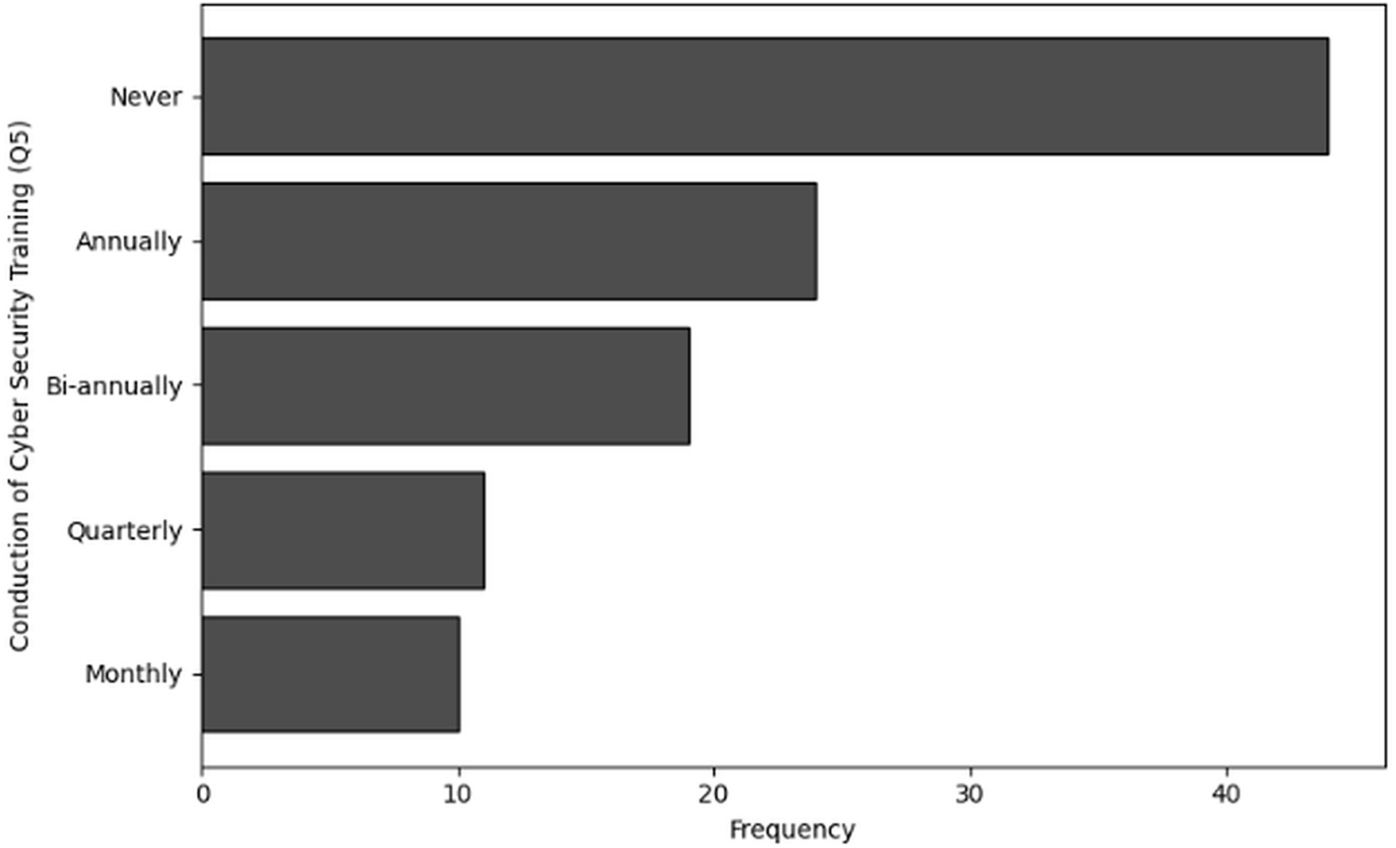
The graphical visualization of frequency analysis for frequency of cybersecurity training (Q5).

**Figure 8:**
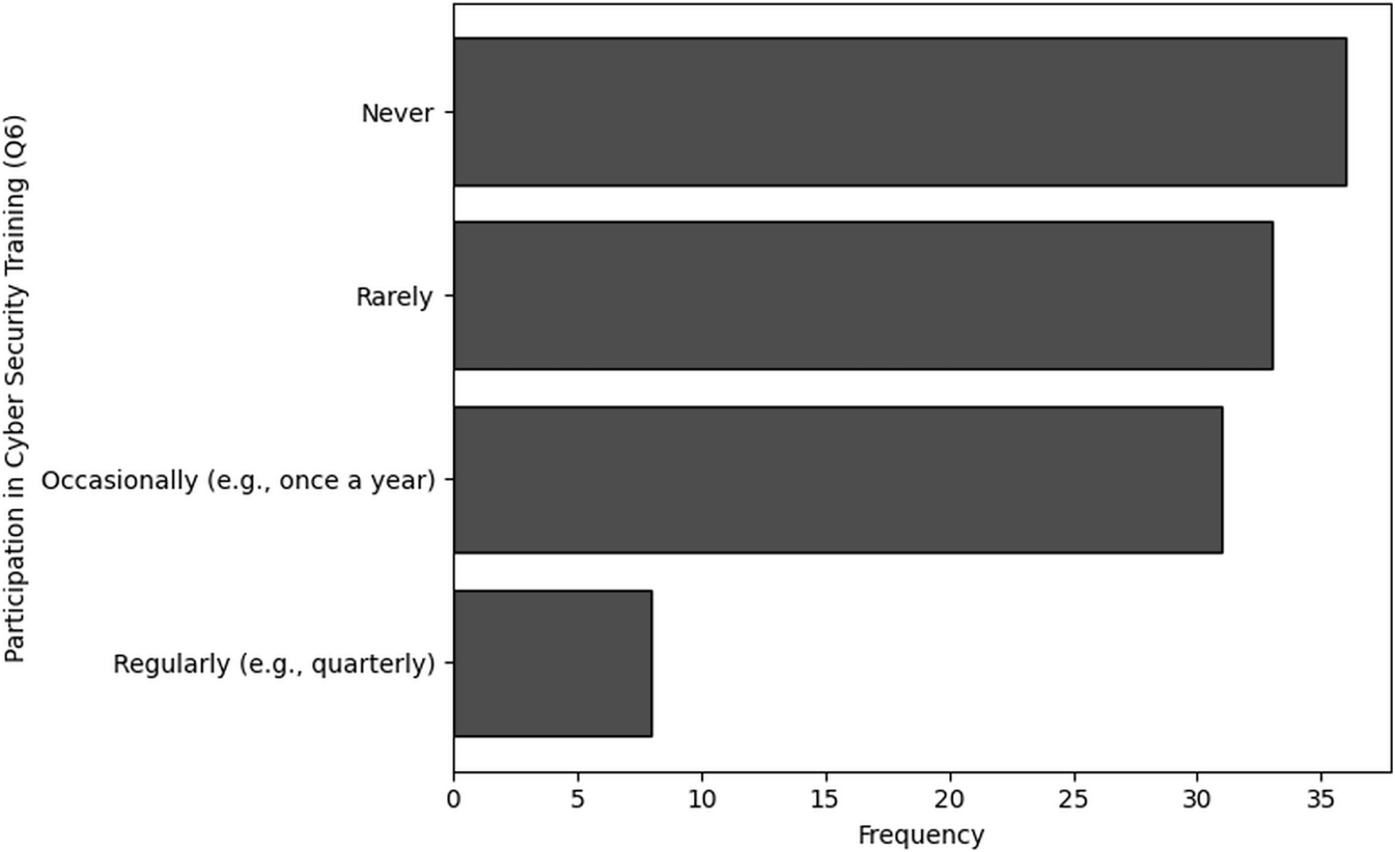
The graphical visualization of frequency analysis for frequency of participation in cybersecurity training (Q6).

A smaller group of 11 respondents indicated they received training quarterly, and only 10 individuals stated that they underwent training on a monthly basis. Similarly, the analysis of Q6, which focused on the availability of training, shows that 36 respondents indicated that training was never available to them. Furthermore, 33 individuals noted that training was rarely accessible, while 31 respondents mentioned that training was available occasionally, such as once a year. Only eight respondents reported that training was available to them regularly, such as on a quarterly basis. The visual representations of frequency analysis are shown in Figs. 7 and 8.

**Key observations drawn from the results of frequency analysis:** The conclusions drawn from the frequency analysis to analyze the Frequency and Availability of Training in cybersecurity dealing with NGS data are presented below. The findings present significant insights:

**Frequency of cybersecurity training (Q5):** The results of frequency analysis obtained in terms of cybersecurity training specific to NGS concludes that majority of organization dealing with NGS data never provide any training related to cybersecurity. Many organizations provide this training annually, but a very small number of organizations opt for frequent training and awareness programs related to cybersecurity.

**Frequency of participation in cybersecurity training (Q6):** The results of frequency analysis obtained in terms of the organization’s participation in cybersecurity training or awareness programs specifically tailored for NGS data security concludes that A considerable percentage of participants (*n* = 36) never engage in cybersecurity training for NGS data protection, while numerous others do very rarely (*n* = 33). Only 28% respondents occasionally and 7% individuals engage regularly to participate in trainings related NGS based cybersecurity.

#### Cross-tabulation

**Figure 9:**
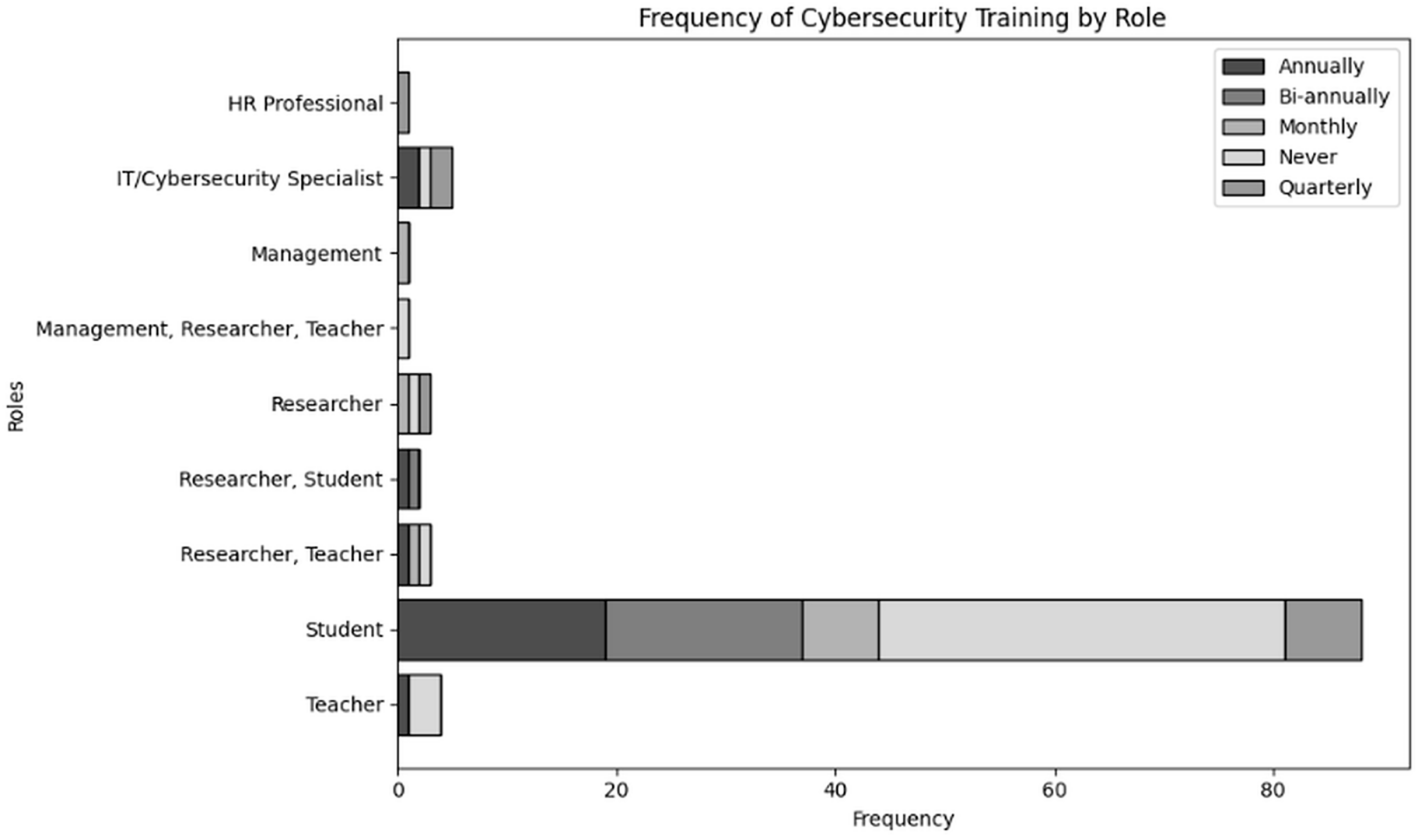
Visual representation of frequency of cybersecurity trainings in organization specifically tailored to NGS operations by role.

**Figure 10:**
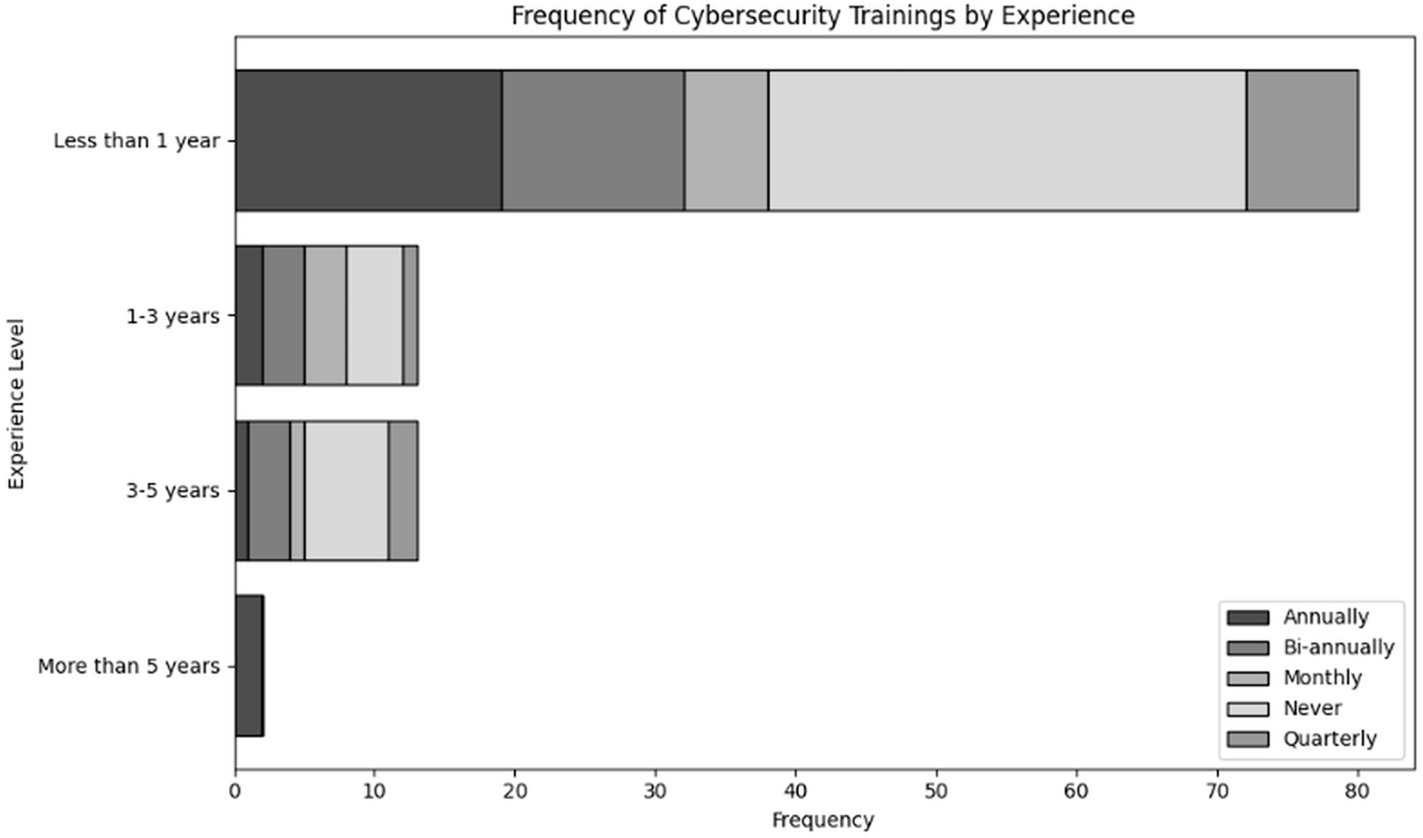
Visual representation of frequency of cybersecurity trainings in organization specifically tailored to NGS operations by experience.

**Figure 11:**
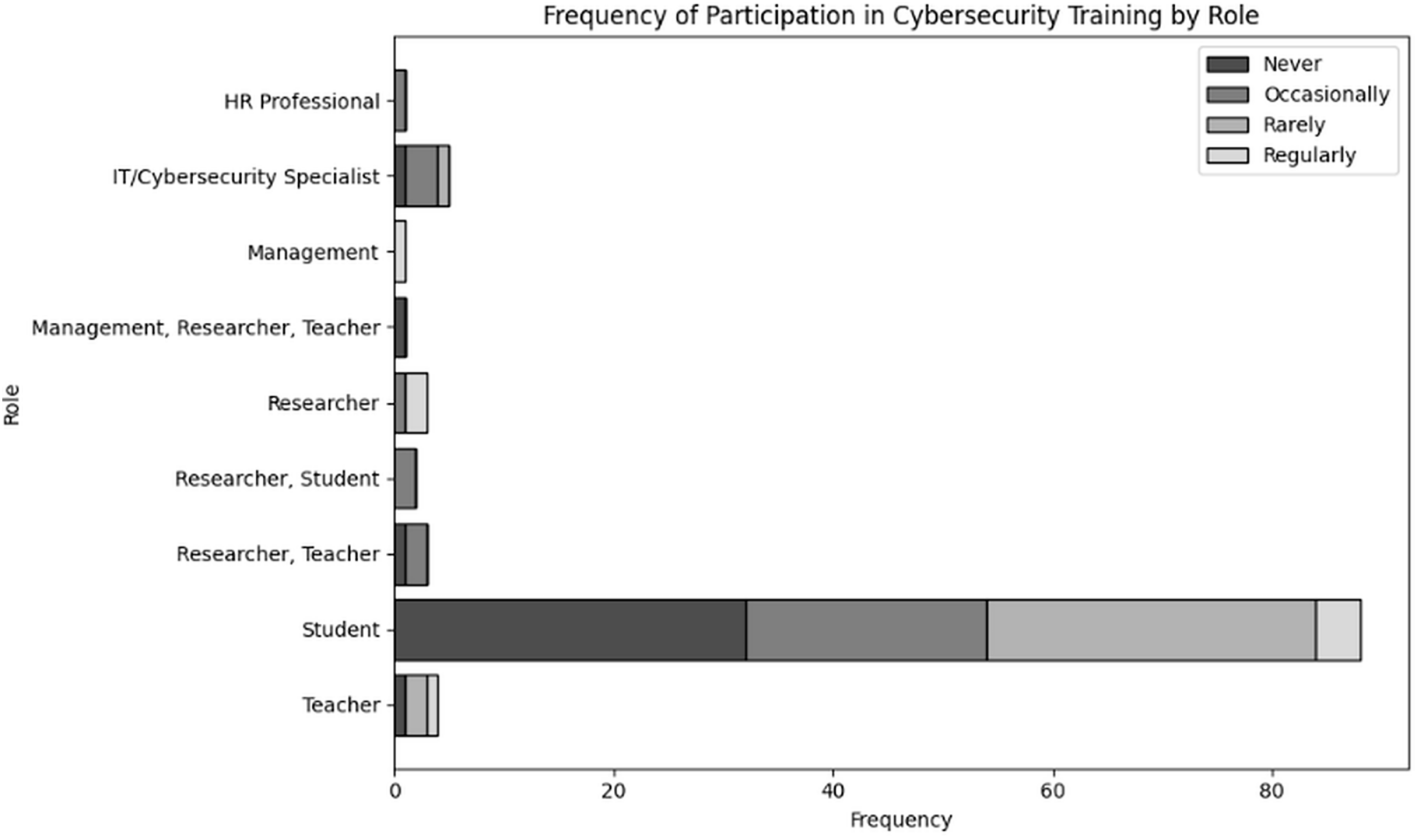
Visual representation of frequency of participation in cybersecurity trainings specifically tailored to NGS operations by role.

**Figure 12:**
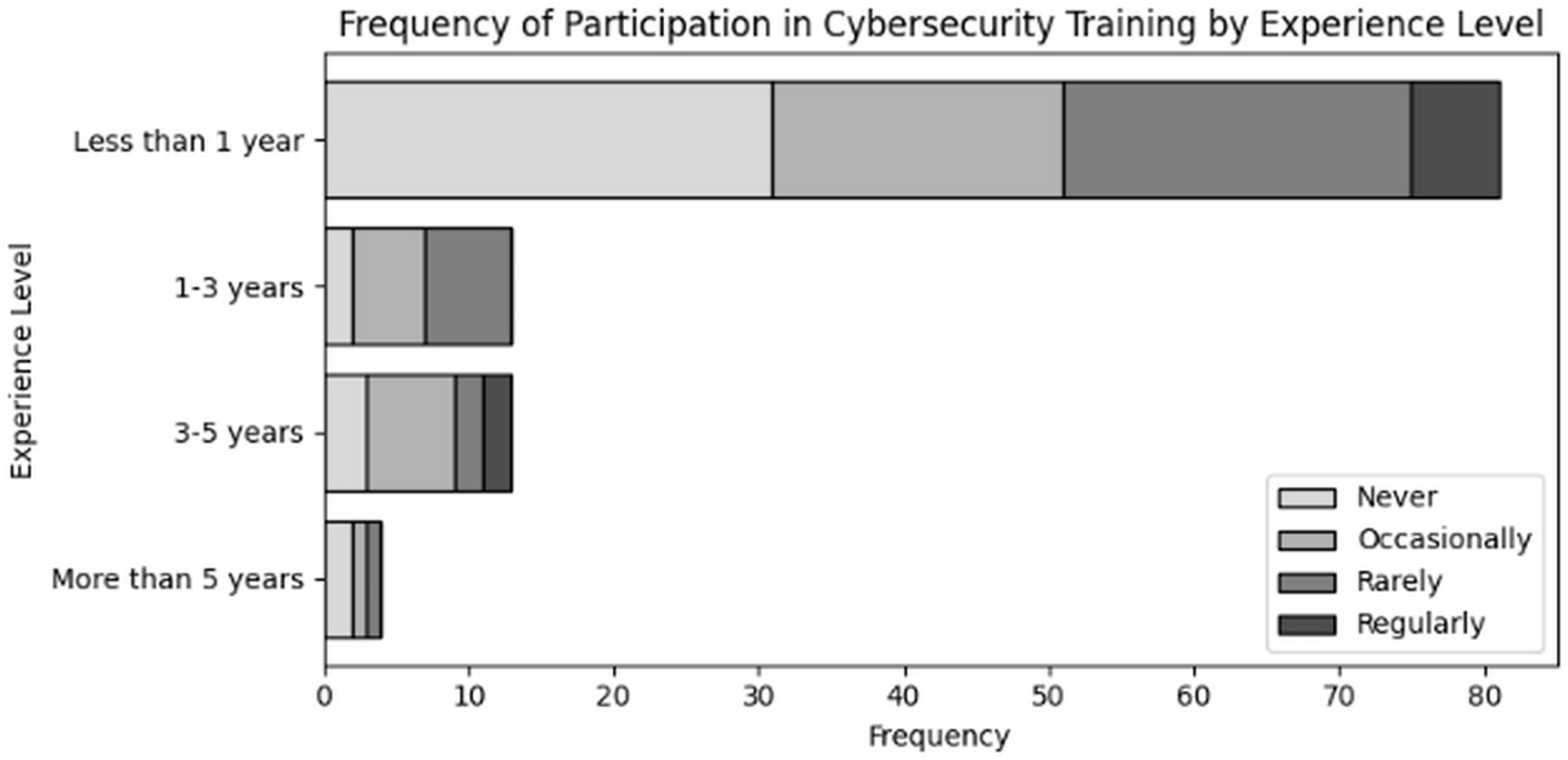
Visual representation of frequency of participation in cybersecurity trainings specifically tailored to NGS operations by experience.

Evaluate organizational actions in relation to various roles of user and levels of expertise inside an organization dealing with NGS data. The results of cross tabulation based on user inputs for frequency of cybersecurity training in organization specifically tailored to NGS operations by role and experience are shown in Tables 7 and 8, respectively. Additionally, the results of cross tabulation based on user inputs for frequency of participation in cybersecurity training specifically tailored to NGS operations by role and experience are shown in Tables 9 and 10, respectively. The visual representations of cross tabulation analysis are shown in Figs. 9, 10, 11 and 12.

**Table 7:** Frequency of cybersecurity trainings in organization specifically tailored to NGS operations by role.

Role	Annually	Bi-annually	Monthly	Never	Quarterly
HR professional	0	0	0	0	1
IT/Cybersecurity specialist	2	0	0	1	2
Management	0	0	1	0	0
Management, Researcher, Teacher	0	0	0	1	0
Researcher	0	0	1	1	1
Researcher, Student	1	1	0	0	0
Researcher, Teacher	1	0	1	1	0
Student	19	18	7	37	7
Teacher	1	0	0	3	0

**Table 8:** Frequency of cybersecurity trainings in organization specifically tailored to NGS operations by experience.

Experience	Annually	Bi-annually	Monthly	Never	Quarterly
Less than 1 year	19	13	6	34	8
1–3 years	2	3	3	4	1
3–5 years	1	3	1	6	2
More than 5 years	2	0	0	0	0

**Table 9:** Frequency of participation in cybersecurity training specifically tailored to NGS operations by role.

Role	Never	Occasionally	Rarely	Regularly
HR professional	0	1	0	0
IT/Cybersecurity specialist	1	3	1	0
Management	0	0	0	1
Management, Researcher, Teacher	1	0	0	0
Researcher	0	1	0	2
Researcher, Student	0	2	0	0
Researcher, Teacher	1	2	0	0
Student	32	22	30	4
Teacher	1	0	2	1

**Table 10:** Frequency of participation in cybersecurity training specifically tailored to NGS operations by experience.

Experience	Never	Occasionally	Rarely	Regularly
Less than 1 year	31	20	24	6
1–3 years	2	5	6	0
3–5 years	3	6	2	2
More than 5 year	2	1	1	0

**Key observations drawn from the results of frequency analysis:** The conclusions drawn from the frequency analysis to analyze the influence of different roles and experiences on the frequency and availability of training in cybersecurity dealing with NGS data are presented below. The findings present significant insights:

Considering Table 7, students are the most common role, with 19 having annual training, 18 biannual, 7 monthly, 37 never receiving training, and 7 quarterly. This suggests that there is a mixed commitment from the organization to student training, with a significant number of students not receiving regular training. Additionally, compared to other positions, IT/cybersecurity professionals have comparatively superior training *i.e*., two receive quarterly and annual training each. However, one respondent never received training, suggesting a possible deficiency in ongoing learning even among cybersecurity professionals. Furthermore, training occurs less frequently in the roles of teachers and researchers/scientists. Due to their responsibilities involving the processing of sensitive NGS data, only a small number receive cybersecurity training on a quarterly or yearly basis, which suggests a lack of regular cybersecurity training.

Considering Table 8, most people who receive training are those with less than a year’s experience; 19 receive training annually, 13 biannually, six monthly, eight quarterly and 34 never receive training. This suggests that there was a considerable initial concentration on training, but that a significant percentage is still not receiving regular training. Additionally, individuals with 1–3 years and 3–5 years experience levels frequently to report “Never” having received training; this is especially true for individuals with 3–5 years experience levels. As individuals become familiar to the organization, there is a decrease in the frequency of training, which could result in outdated security policies. Furthermore, most users with over 5 years of experience receive training annually. This is the result of outdated training resources for long-term users, which could cause organisational vulnerabilities.

Considering Table 9, only four students routinely participate in training, out of the significant number of students (32), who never engaged. Given that students are likely to handle sensitive data while pursuing their education, this suggests a possible disconnect from their understanding of cybersecurity policies. Furthermore, in case of IT/cybersecurity specialist there is no regular participation in training, despite the occasional participation of only three IT specialists. This points to a lack of ongoing professional growth, especially for individuals in the role of cybersecurity maintenance. However, teachers and researchers/scientists, on the other hand, typically have low engagement levels; many of them participate infrequently or never at all. This implies that for keeping the users updated, regarding the security of critical NGS data, there is a need for more participation in cybersecurity training.

Considering Table 10, users with less than a year of experience are less likely to be regular participants, with 31 respondents had never engaged and only six engaging regularly. This indicates that although there is initial training provided, there is no emphasis placed on ongoing participation. Furthermore, in case of the participation of users with 3–5 years of experience, a moderate number of users are participating occasionally. The absence of recommended, continuous training programs are the cause of this trend. Additionally, the participation of individuals with more than 5 years of expertise is also limited. The majority either never participate at all or participate either ocassionally or rarely. This disengagement could put the organisation at risk by resulting in outdated cybersecurity practices and training.

#### Overall conclusion of frequency and availability of training

According to a frequency analysis of cybersecurity training and participation conducted within organisations managing NGS significant gaps exist in the availability and participation of these crucial programs. Concerningly, a significant number of organisations never provide cybersecurity training that is specifically relevant to NGS operations. This indicates that the organisation is not committed to upholding robust cybersecurity standards in this critical field. Even though some organisations do offer training every year (24) or every 2 years (19), this frequency is usually insufficient to keep up with the constantly changing cybersecurity threats subject to NGS data. This lack of organizational priority is also reflected in the participation rates of staff in cybersecurity training. Few responders rarely participate in any kind of training, and a significant number of participants never do so. It indicates that employees do not prioritise training enough, even in situations where it is provided. A small portion of participants frequently engage in cybersecurity training programs specifically designed for NGS data security, implying that there is a need for substantial enhancement in the frequency of training sessions and individuals’ participation.

### Effectiveness and relevance of training

The effectiveness and relevance of cybersecurity training programs within NGS data processing organisations are evaluated in this section. It specifically aims to assess:

**Confidence in identifying threats:** The level of confidence employees has in recognizing cybersecurity threats specific to NGS operations. This reflects the effectiveness of the training in equipping them with the necessary skills and knowledge to identify and respond to potential threats.

**Relevance to day-to-day responsibilities:** How appropriate and beneficial the training information is in the context of the employees’ day-to-day tasks. This makes it easier to determine whether the training will be useful to them in their roles protecting NGS data.

**Application of cybersecurity knowledge:** It evaluates whether users have had the chance to use the knowledge they gained from the training in real-world scenarios. This evaluates the training’s usefulness in equipping users to deal with cybersecurity threats as well as its practical consequences.

**Detection of potential cybersecurity threat:** It evaluates whether the training has made it possible for users or their groups to identify possible cybersecurity risks to NGS or other private information. This is a clear indicator of how well the training improved the workforce’s ability to recognise threats, which in turn improved the organization’s overall security posture. Following questions are considered for this section:

**Q7:** How confident are you in identifying a cybersecurity threat specific to NGS operations?

**Q8:** How relevant do you find the cybersecurity training content to your day-to-day responsibilities?

**Q9:** Since the last cybersecurity training session, have you encountered situations where you applied the knowledge learned?

**Q10:** Have you or anyone in your team ever detected a potential cybersecurity threat to NGS or other sensitive data?

#### Descriptive statistics

**Figure 13:**
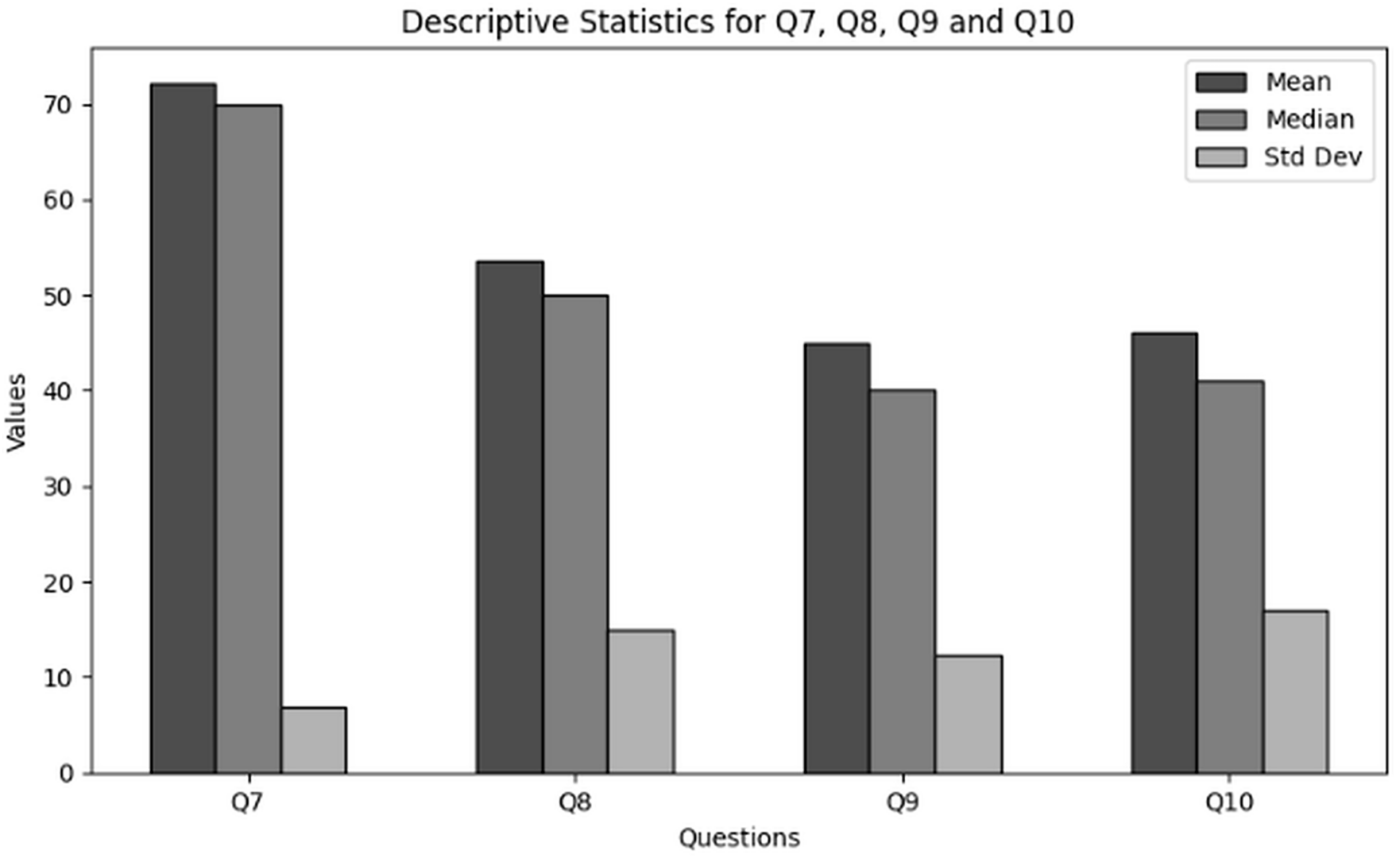
The graphical visualization of descriptive statistics for effectiveness and relevance of training of users through Q7–Q10.

The purpose of descriptive statistics is to summarise the data to obtain an understanding regarding effectiveness and relevance of training of users and their awareness in cybersecurity working with NGS data. This includes calculating means, medians, and standard deviations as shown in Table 11 respectively. For every question (Q7–Q10), the mean, median, and standard deviation are included in the table to provide an overview of the responses’ primary patterns and variations. Figure 13 presents the graphical visualization of the descriptive statistics to summarize the values of mean, median and standard deviations.

**Table 11:** Results of descriptive statistics for effectiveness and relevance of training of users in cybersecurity dealing with NGS data.

Statistics	Q7	Q8	Q9	Q10
Mean	72.24	53.68	44.99	46.06
Median	70.00	50.00	40.00	41.00
Std	6.85	14.98	12.30	16.93

**Key observations drawn from the results of frequency analysis:** The conclusions drawn from the descriptive statistics to analyze the effectiveness and relevance of training of users in cybersecurity dealing with NGS data through Q7, Q8, Q9, and Q10 are presented below. The findings present significant insights:

**Confidence in identifying cybersecurity threats (Q7):** According to the results descriptive statistics most participants are reasonably confident in their abilities to recognize cybersecurity threats related to NGS operations. Furthermore, a large number of respondents are not sure feel confident in their abilities. Most respondents’ confidence levels are clustered near the mean, as indicated by the low standard deviation, which suggests that confidence is generally stable throughout the group of respondents.

**Relevance to day-to-day responsibilities (Q8):** The results indicate that currently the respondents have the perspective of neutral to slightly acceptable towards the relevance of cybersecurity to day-to-day responsibilities while dealing with NGS. However, many of them finds the relevance somewhat irrelevant NGS data. This shows that there is great potential to enhance the way cybersecurity training is tailored to satisfy the tasks and responsibilities of individuals based on their roles.

**Application of cybersecurity knowledge (Q9):** The statistical results obtained in terms of the application of the conducted trainings within the organization working with NGS data conclude that the application of the concepts gained since the last training session in an organization is moderately high. However, many individuals utilize the skills at hand, others practice less frequently.

**Detection of potential cybersecurity threat (Q10):** According to the results descriptive statistics most participants indicate that although some teams have identified cybersecurity threats, this is not a frequent event. It suggests a decrease in threat activity or difficulties in detection. Furthermore, lack of major outliers in the response distribution is also indicated. Further evidence of increased variability in respondents’ and their teams’ experience with the detection of cybersecurity threats derives from the larger standard deviation. This indicates variations in the ability of threat detection among organisations or varying degrees of exposure to threats.

#### Frequency analysis

**Figure 14:**
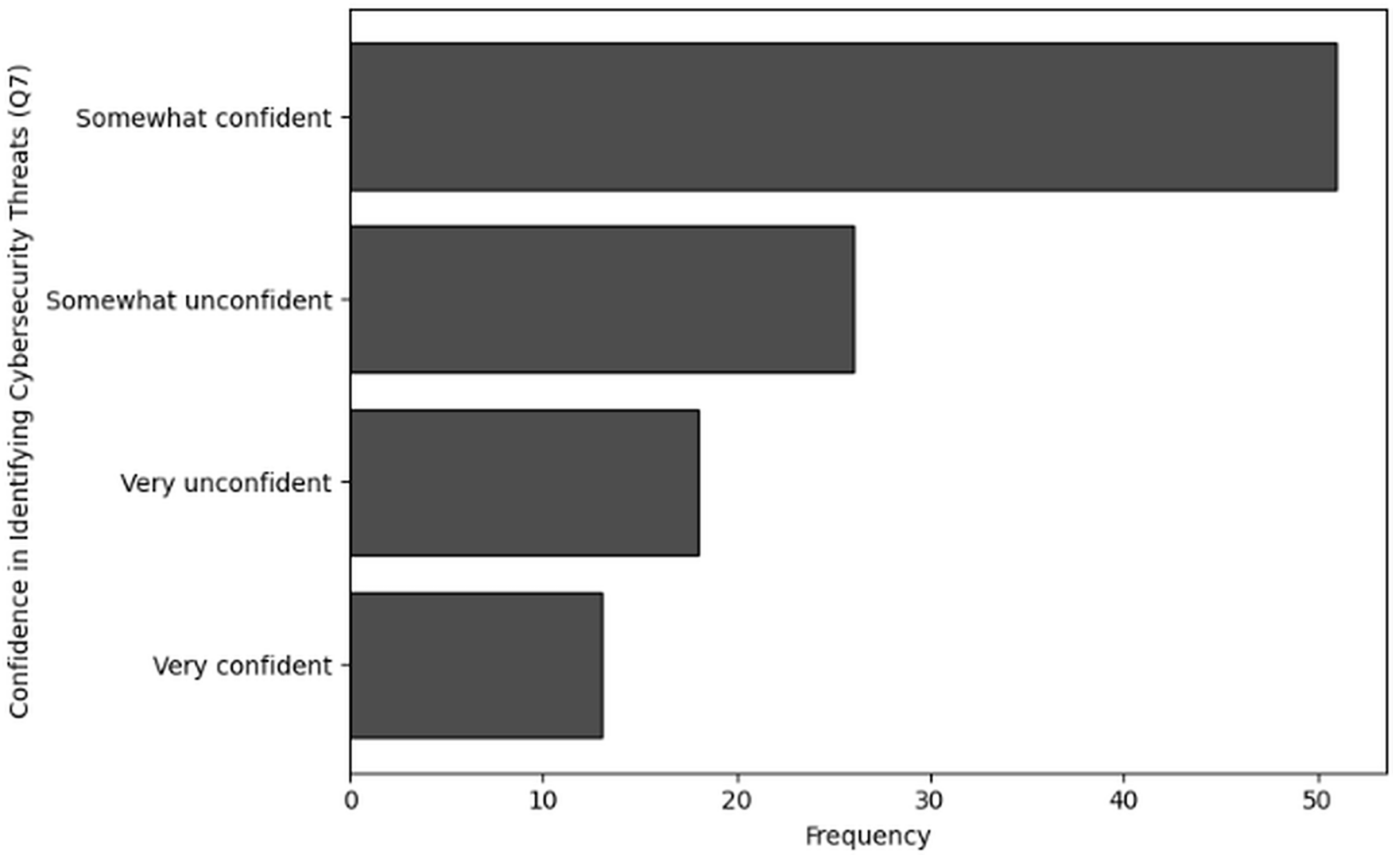
The graphical visualization of frequency analysis for confidence in identifying cybersecurity threats (Q7).

**Figure 15:**
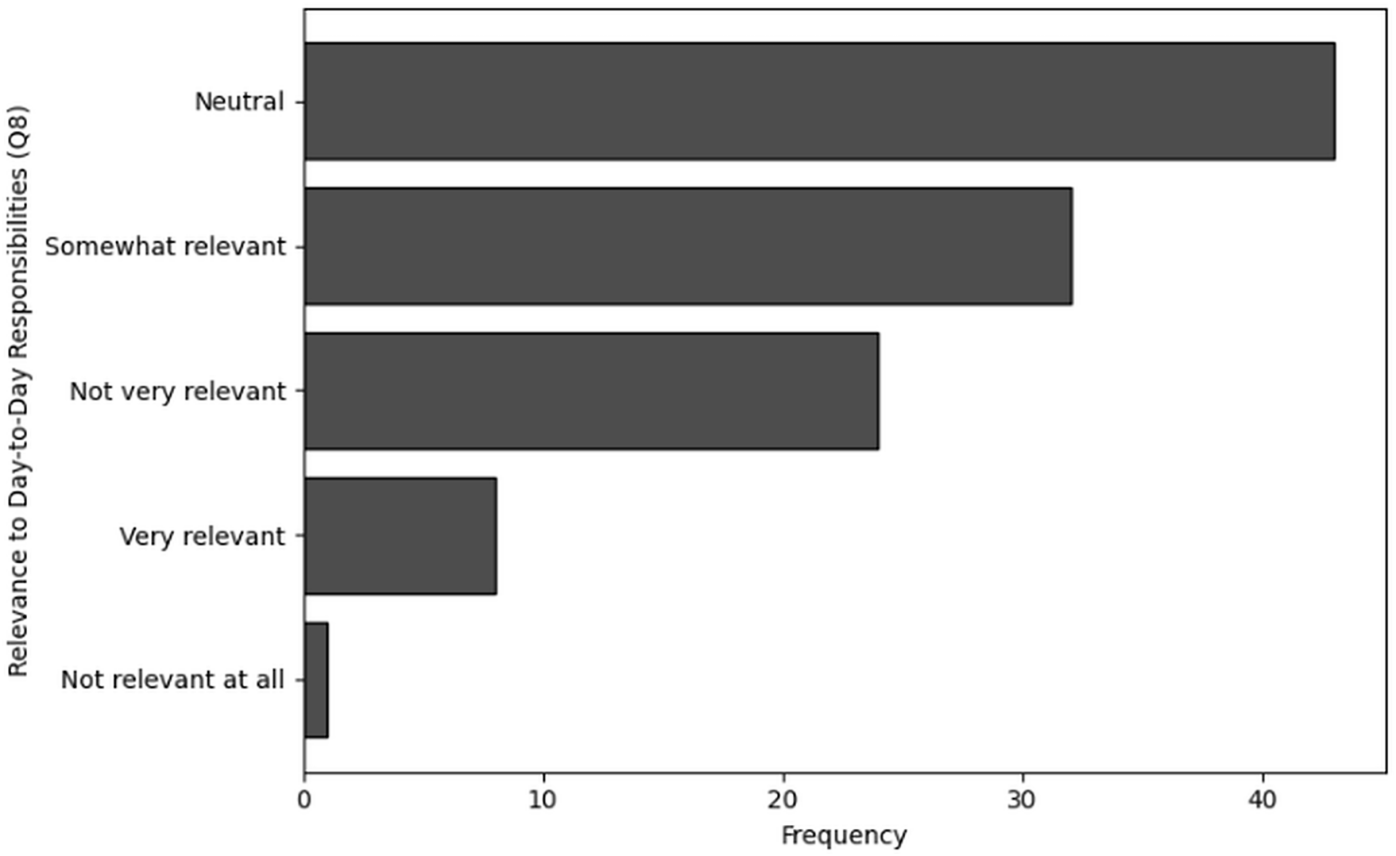
The graphical visualization of frequency analysis for relevance to day-to-day responsibilities (Q8).

**Figure 16:**
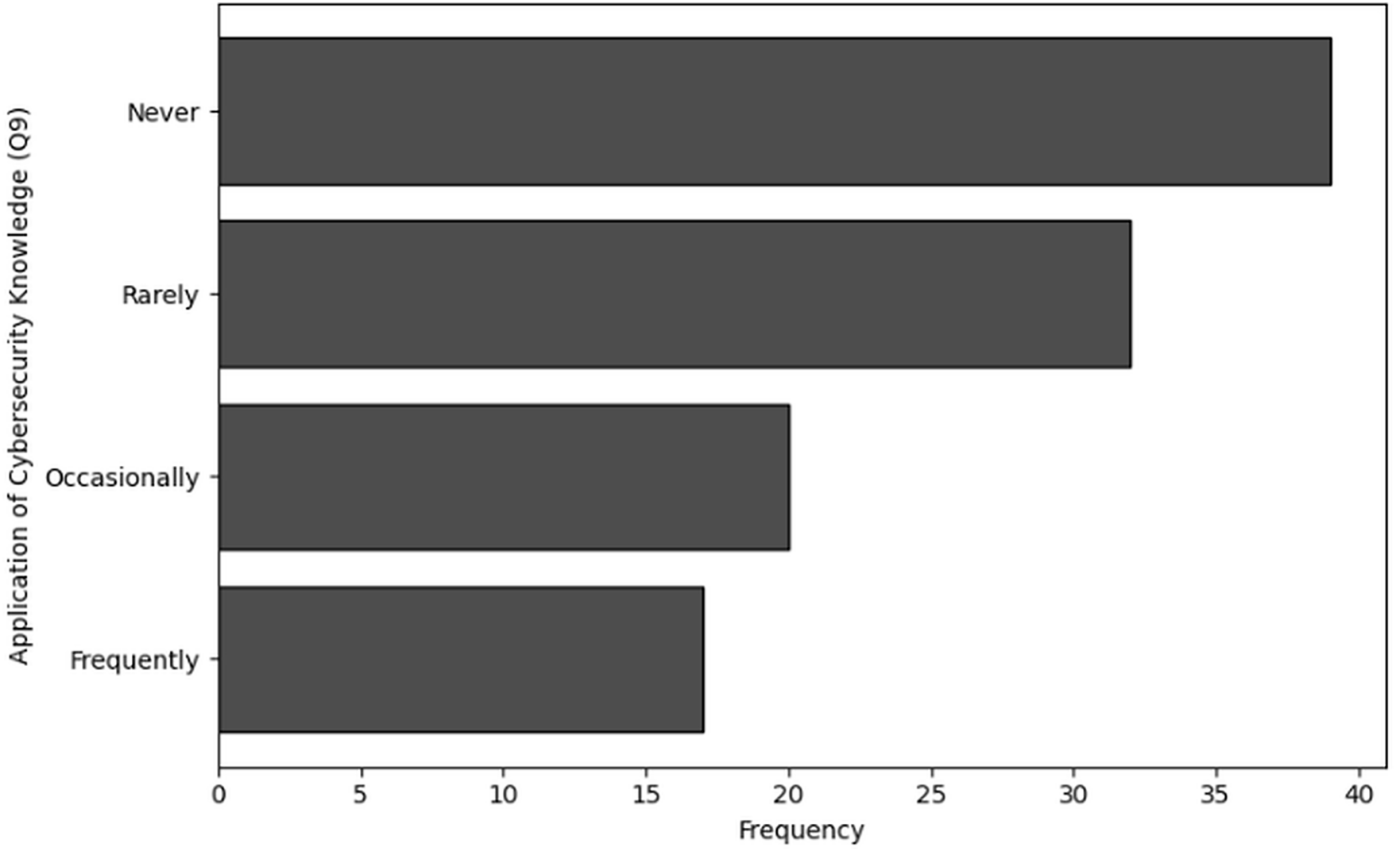
The graphical visualization of frequency analysis for application of cybersecurity knowledge related to NGS (Q9).

**Figure 17:**
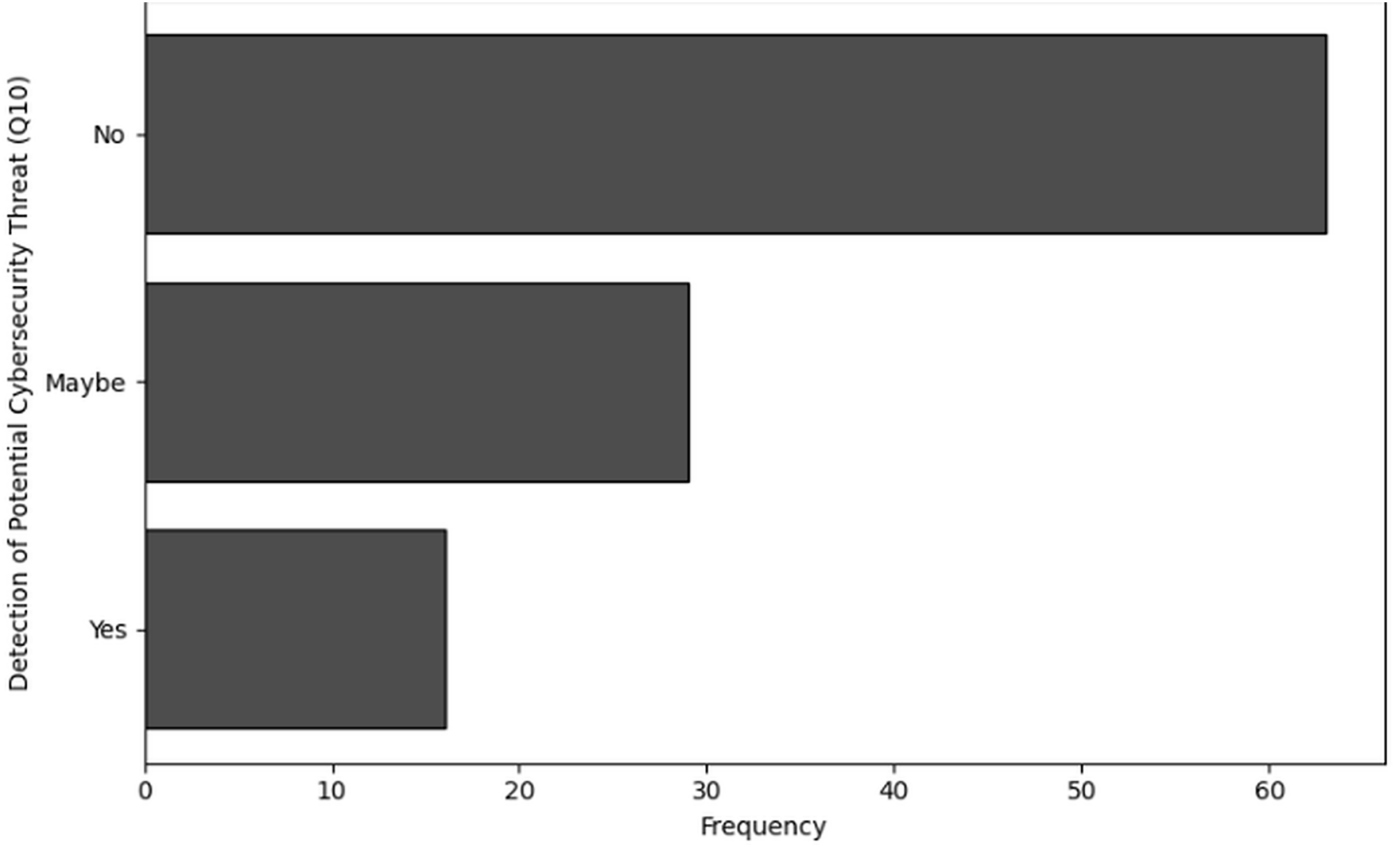
The graphical visualization of frequency analysis for detection of potential cybersecurity threats related to NGS (Q10).

The purpose of frequency analysis is to summarize the identification of common activities and behaviors in terms of the effectiveness and relevance of training of users in cybersecurity dealing with NGS data by counting the number of inputs for each category. The results of responses to analyze the frequency of user inputs for confidence in identifying cybersecurity threats (Q7), relevance of cybersecurity training in day-to-day responsibilities (Q8), application of cybersecurity knowledge (Q9) and detection of potential cybersecurity threat (Q10) are shown in Tables 12, 13, 14 and 15, respectively. The visual representations of frequency analysis for Q7, Q8, Q9 and Q10 are shown in Figs. 14, 15, 16, and 17, respectively.

**Table 12:** Results of frequency analysis for confidence in identifying cybersecurity threats (Q7).

Confidence in identifying cybersecurity threats (Q7)	Frequency
Somewhat confident	51
Somewhat unconfident	26
Very unconfident	18
Very confident	13

**Table 13:** Results of frequency analysis for relevance of cybersecurity training in day-to-day responsibilities (Q8).

Relevance to day-to-day responsibilities (Q8)	Frequency
Neutral	43
Somewhat relevant	32
Not very relevant	24
Very relevant	8
Not relevant at all	1

**Table 14:** Results of frequency analysis for the application of cybersecurity knowledge (Q9).

Application of knowledge (Q9)	Frequency
Never	39
Rarely	32
Occasionally	20
Frequently	17

**Table 15:** Results of frequency analysis for the detection of potential cybersecurity threat (Q10).

Detection of threats (Q10)	Frequency
No	63
Maybe	29
Yes	16

**Key observations drawn from the results of frequency analysis:** The conclusions drawn from the frequency analysis to analyze the effectiveness and relevance of training in cybersecurity dealing with NGS data are presented below. The findings present significant insights:

**Confidence in identifying cybersecurity threats (Q7):** Fifty-one respondents feel “Somewhat confident” about their ability to identify cybersecurity threats unique to NGS operations. Although, with 26 respondents reporting feeling “Somewhat unconfident” and 18 reporting “Very unconfident,” there is a noticeable inclination towards lower confidence levels. Only 13 respondents indicated that they were “Very confident”. Although there appears to be a moderate level of confidence, this distribution indicates that many respondents need more training before experiencing completely comfortable in the ability to recognise cybersecurity threats.

**Relevance to day-to-day responsibilities (Q8):** The frequency analysis of Q8 shows that the majority of responses are neutral, with most respondents ( $n = 43$) neither agreeing nor disagreeing with the relevance of cybersecurity to their daily work. This suggests that many users may lack sufficient awareness or understanding to assess its relevance. Additionally, a significant number of respondents ( $n = 32$) find it “somewhat relevant,” indicating a moderate level of understanding when it comes to cybersecurity in their routine responsibilities.

However, an observable group of respondents ( $n = 24$) consider it “not very relevant,” reflecting a disconnect between the users involved in NGS workflows and the cybersecurity training they receive. Only a small number ( $n = 8$) find the training clearly relevant to their day-to-day duties. Overall, these results indicate that while some value is recognized, substantial improvements are needed to ensure cybersecurity training aligns more closely with users’ operational responsibilities.

**Application of cybersecurity knowledge (Q9):** According to the frequency analysis for question 9, a significant number of respondents *i.e*., 39 never utilise the knowledge they have gained from cybersecurity training. Additionally, many respondents (*n* = 32) rarely encountered such situations. Furthermore, only 17 users frequently and 20 users occasionally apply what they have learned. This pattern suggests that either there are few opportunities to use the training, or the instruction does not be sufficiently encouraging for employees to use the knowledge in their work scenarios. It also implies a gap between the training that is given and the practical application of this knowledge.

**Detection of potential cybersecurity threat (Q10):** Only 16 respondents experienced definite evidence of risks to cybersecurity, a limited number (*n* = 29) of respondents are unsure (“Maybe”). Furthermore, a significant number of respondents (*n* = 63) clearly never detect any potential threat. This shows that although certain individuals or groups identify cybersecurity risks, this ability is not common. This indicates that organisations need to improve their detection capabilities or that threats do not occur regularly.

#### Chi-square test

**Figure 18:**
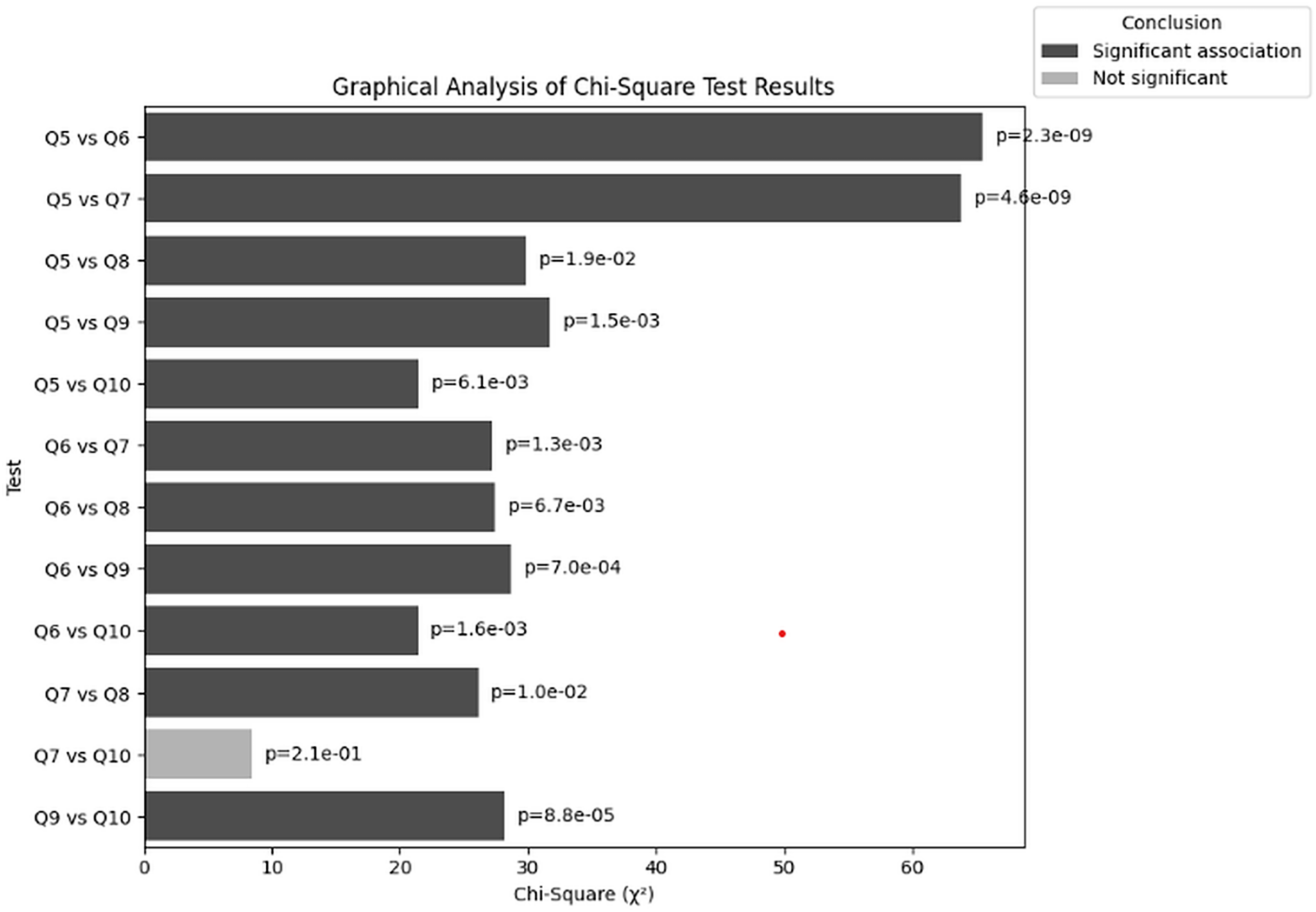
Graphical visualization of chi-square ( $\bf \chi^2$) test.

The purpose of the chi-square test is to find the correlations between cybersecurity training and effectiveness of training by using Q5–Q10. This facilitates the understanding that the training provided by organizations dealing with NGS data are either effective for users or further improvements are required. This test concludes that whether users understand the necessary knowledge to recognise or perceive any cybersecurity related threat. Table 16 summarizes the chi-square statistics, *p*-values, degrees of freedom (df), and expected frequencies. The chi-square test findings are graphically shown in Fig. 18. Significant relationships are shown by red bars, whereas non-significant associations are shown by blue bars. For reference, the *p*-values are given next to each bar. An overview of the tests that are conducted and their significance levels is provided in this visualization.

**Table 16:** Results obtained from the chi-square test through Q5–Q10.

Test	Chi-square ( $\bf \chi^2$)	*p*-value	df	Conclusion
Q5 *vs* Q6	65.43	2.27E−09	12	Significant association
Q5 *vs* Q7	63.78	4.57E−09	12	Significant association
Q5 *vs* Q8	29.83	0.0189	16	Significant association
Q5 *vs* Q9	31.74	0.0015	12	Significant association
Q5 *vs* Q10	21.42	0.0061	8	Significant association
Q6 *vs* Q7	27.23	0.0013	9	Significant association
Q6 *vs* Q8	27.41	0.0067	12	Significant association
Q6 *vs* Q9	28.64	0.0007	9	Significant association
Q6 *vs* Q10	21.37	0.0016	6	Significant association
Q7 *vs* Q8	26.09	0.0104	12	Significant association
Q7 *vs* Q10	8.47	0.2059	6	Not significant
Q9 *vs* Q10	28.16	8.76E–05	6	Significant association

**Key Observations drawn from the results of chi-square ( ${\chi^2}$) test:** The results of the chi-square tests provide significant details about how frequency of the conduction of trainings relate to users’ cybersecurity understanding and behaviour in the context of NGS. The findings present significant insights:

**Significant association between training frequency (Q5) and user participation (Q6): **There is a significant association (( ${\chi^2}$) = 65.43, *p* = 2.27E−09) between the frequency with which organizations offer cybersecurity training (Q5) and the frequency with which users participate in it (Q6). This suggests that the frequency with which the organization offers training has a significant impact on how many employees participate. More users’ involvement is probably increased by more frequent training sessions.

**Significant relationship between frequency of training (Q5) and confidence in identification of threat (Q7):** The frequency of training provided by organizations (Q5) and employees’ confidence in recognizing cybersecurity threats (Q7) significantly correlate with (( ${\chi^2}$) = 63.78, *p* = 4.57E−09). This shows that increasing the frequency of training always result into users perceiving enhanced abilities of detecting threats.

**Significant relationship between frequency of training (Q5) and relevancy of training content (Q8):** According to the observed chi-square test (( ${\chi^2}$) = 29.83, *p* = 0.0189) there is a significant relation between training frequency (Q5) and the perceived relevance of the training content to everyday responsibilities (Q8). This indicates that frequency of cybersecurity training directly impacts the relevancy of the training material.

**Significant association between frequency of training (Q5) and application of knowledge (Q9): **The frequency with which organizations offer cybersecurity training (Q5) and the extent to which users applied what they have learned in real-world circumstances (Q9) are significantly correlated (( ${\chi^2}$) = 31.75, *p* = 0.0015). This shows that employees are more likely to apply what they have learned when they receive training more frequently, highlighting the usefulness of regular trainings.

**Significant association between training frequency (Q5) and possibility threat detection (Q10):** There is a significant association (( ${\chi^2}$) = 21.42, *p* = 0.0061) between the frequency of training (Q5) and the assertion of whether users have identified potential cybersecurity threats (Q10) according to the chi-square test. This indicates that employees’ capacity to identify possible threats may be enhanced by frequent training, proving the value of ongoing training initiatives.

**Significant association between participation in training (Q6) and confidence in identification of threat (Q7):** The chi-square test between users’ training participation (Q6) and the confidence of respondents in identifying threats (Q8) showed significant relation (( ${\chi^2}$) = 27.23, *p* = 0.0013). Therefore, individuals who frequently participate in cybersecurity training sessions organized for NGS data protection typically exhibit more confidence in their potential to identify cybersecurity risks particular to their organization. On the other hand, individuals who rarely or never take part in training are probably less confident in their ability to identify and address cybersecurity issues.

**Significant association between participation in training (Q6) and relevance of training content (Q8): **The chi-square test between users’ training participation (Q6) and the perceived relevance of the training content (Q8) showed significant association (( ${\chi^2}$) = 27.41, *p* = 0.0067), which is consistent with the association between Q5 and Q8. Therefore, this significant relation shows that the relevance of cybersecurity training content to the responder’s day-to-day responsibilities is significantly related to the individual’s participation in training.

**Significant association between application of knowledge (Q9) and participation in training (Q6): **There is a strong correlation (( ${\chi^2}$) = 28.64, *p* = 0.0007) between the application of knowledge (Q9) and training participation (Q6). This indicates that users who regularly attend training sessions are more likely to put the knowledge they have learned to use, which emphasizes the significance of participating fully in training sessions.

**Significant association between identifying potential threats (Q10) and taking part in training (Q6):** A notable correlation has been observed between the frequency of training participation by individuals (Q6) and their capacity to identify possible cybersecurity threats (Q10) (( ${\chi^2}$) = 21.37, *p* = 0.0016). This result implies that training program participation is essential for improving employees’ ability to identify threats.

**Significant association between relevancy of training content (Q8) and confidence in identifying threats (Q7):** According to the results of chi-square test, there is a significant correlation (( ${\chi^2}$)= 26.09, *p* = 0.0104) between the perceived relevance of the training content (Q8) and confidence in identifying threats (Q7). This suggests that the more focused and relevant training directly impacts to improve the responders confidence level while identifying threats.

**No significant association between possibility threat detection (Q10) and confidence in identifying threats (Q7):** According to the results of chi-square test, there is a no significant correlation (( ${\chi^2}$)= 8.47, *p* = 0.2059) between the possibility of threat detection (Q10) and confidence in identifying threats (Q7). This suggests the users have confidence in identifying threats related to NGS doesn’t always have the ability to detect all threats related to NGS.

**Significant association between application of knowledge (Q9) and identification of threats (Q10):** Finally, according to the chi-square test there is a significant association (( ${\chi^2}$) = 28.16, *p* = 8.76E−05) between the application of knowledge (Q9) and the identification of possible threats (Q10). Therefore, individuals who exhibit active participation in trainings will gain more knowledge regarding the applications of cybersecurity related to NGS. In that way, eventually they will more easily identify real world threats.

Overall, the data shows a strong connection between the frequency of cybersecurity training along with involvement and the ability to utilize information practically and identify possible threats. These variables, however, don’t always transfer into better confidence in identifying threats or awareness that the training material is relevant, indicating that other elements such as the level of expertise and focus of the organization are significant to its effectiveness.

#### Overall conclusion of effectiveness and relevance of training

There are varying findings when evaluating the usefulness and effectiveness of cybersecurity training in organisations that handle NGS data. Most respondents declare to be moderately confident in their ability to recognise cybersecurity threats particular to NGS operations, but significant number of respondents are unsure or lack confidence, suggesting that the training does not have provided all users with the required skills. Although the training is partially connected with everyday duties, its relevance to day-to-day demands is seen as neutral, indicating that it may not be fully integrated or instantly applicable in practise. Furthermore, a significant number of respondents utilise training knowledge infrequently or never at all, suggesting a possible gap between the training itself and its application in real-world scenarios. Furthermore, most responders failed to identify any cybersecurity concerns, indicating that threat detection capabilities within these organisations have not yet been considerably improved by the training. While the training programs do have some effect overall, there is plenty of room for improvement in terms of making the training more appropriate, relevant, and efficient in terms of enhancing cybersecurity protocols related to NGS data.

### Barriers and challenges

This section evaluates the challenges and barriers that organizations encounter while putting in place efficient cybersecurity training initiatives. It aims to specifically identify:

**Organisational restrictions:** Any internal constraints, such financial constraints, a shortage of resources, or a lack of time set aside for training. Knowing these limitations can help to emphasise the need for improved or increased funding for cybersecurity education.

**Problems with users’ engagement:** Factors that affect user involvement, include a lack of interest, a belief that the content is irrelevant, or the challenge of obtaining training opportunities. These observations can assist organisations in creating training materials that are easier to use, more interesting, and valued by users working with NGS data.

**Training program effectiveness:** Challenges related to the quality and delivery of the training programs, such as outdated materials, inadequate trainers, or lack of hands-on, practical training opportunities. This can guide improvements in training content and methodologies.

**Cultural and structural barriers:** Broader organizational culture and structural issues that might impede effective training, such as a lack of support from leadership, insufficient emphasis on cybersecurity within the organizational culture, or fragmented communication channels.

Following question is considered for this section:

**Q11:** In your opinion, what are the biggest barriers to implementing effective cybersecurity training in your organization? (Select all that apply).

#### Descriptive statistics

**Figure 19:**
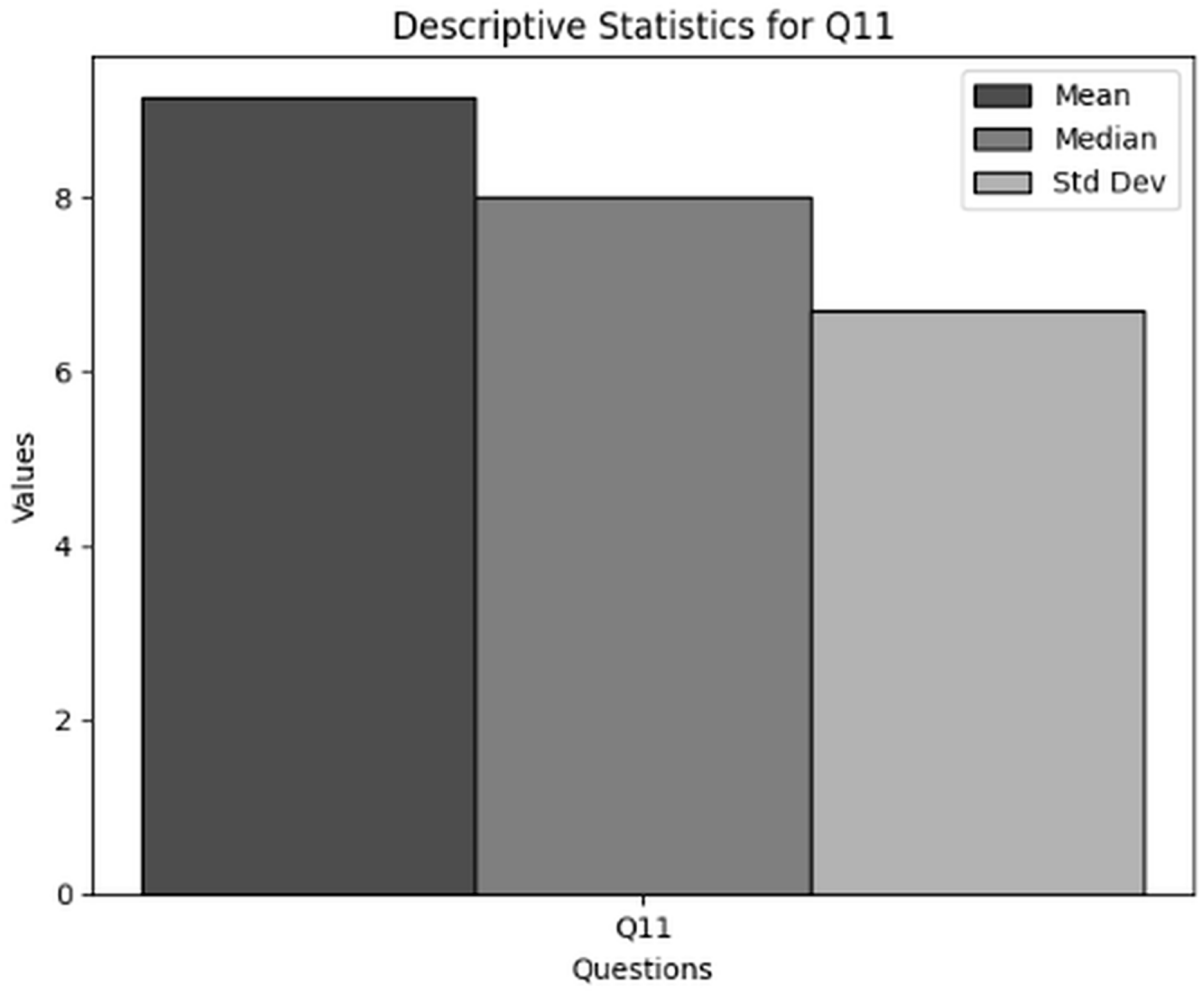
The graphical visualization of descriptive statistics for barriers and challenges faced by users (Q11).

The purpose of descriptive statistics is to summarise the data to obtain an understanding regarding the barriers and challenges faced by of users in cybersecurity working with NGS data. This includes calculating means, medians, and standard deviations as shown in Table 17. For question Q11, the mean, median, and standard deviation are included in the table to provide an overview of the responses’ primary patterns and variations. Figure 19 presents the graphical visualization of the descriptive statistics to summarize the values of mean, median and standard deviations.

**Table 17:** Results of descriptive statistics for effectiveness and relevance of training of users in cybersecurity dealing with NGS data through Q11.

Statistics	Q11
Mean	9.16
Median	8.00
Std	6.69

**Key observations drawn from the results of descriptive statistics:** The conclusions drawn from the descriptive statistics to analyze the challenges and barriers that organizations encounter while putting in place efficient cybersecurity training initiatives through Q11 are presented below. The findings present significant insights:

**Barriers to effective training (Q11):** The statistical results obtained in terms identifying the biggest barriers to implementing effective cybersecurity training in the organization concludes that the obstacles for implementing an efficient cybersecurity training are quite fluctuating. A negative skew is shown by the median value smaller than the mean. This shows that most of the organizations have reported fewer and some reported numerous barriers for implementing cybersecurity in organizations working with NGS data.

#### Frequency analysis

**Figure 20:**
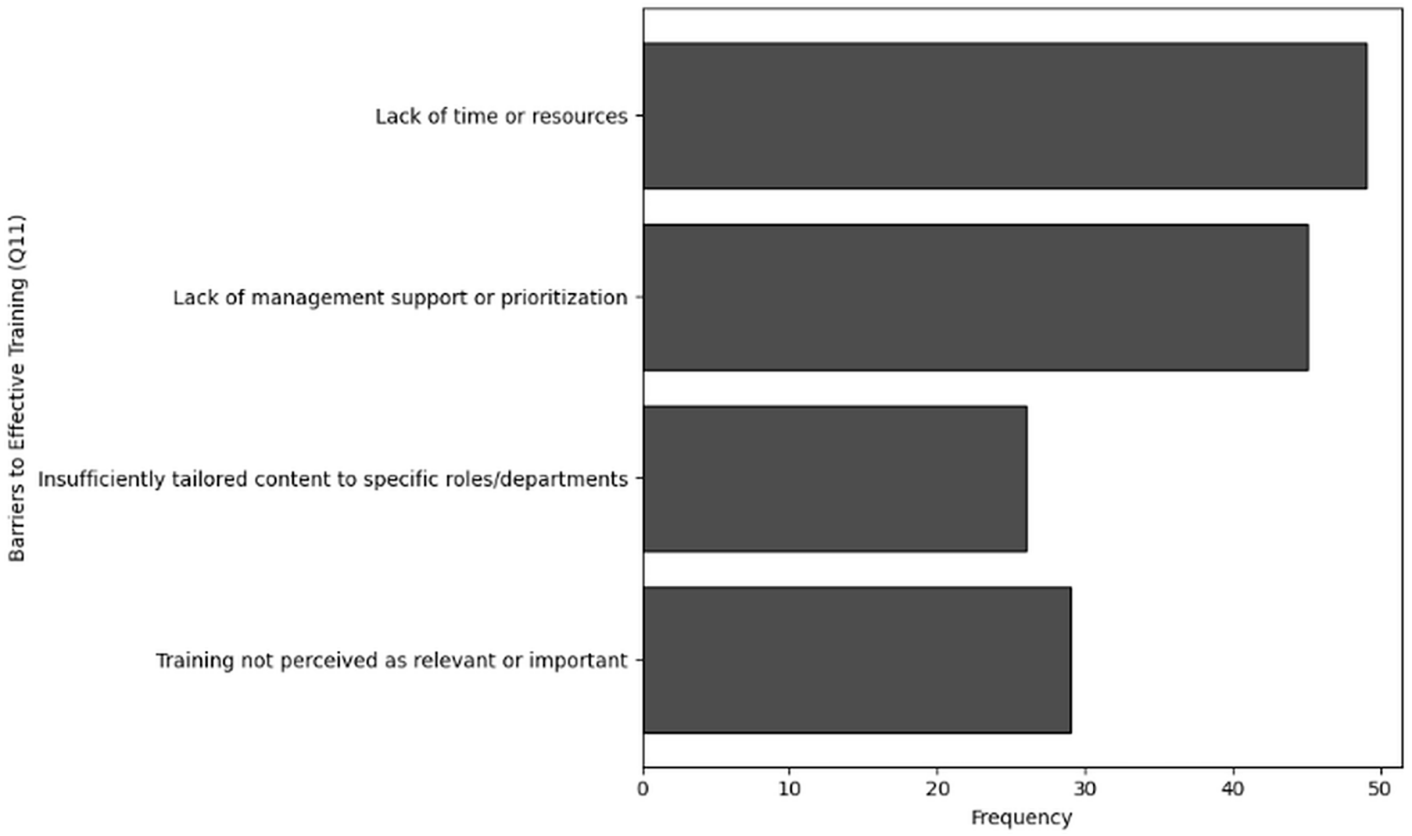
The graphical visualization of frequency analysis for common challenges and barriers related to NGS through Q11.

The purpose of frequency analysis is to summarize the identification of common challenges and barriers by counting the number of inputs for each category. The result of responses to analyze the frequency of user inputs is shown in Table 18. The visual representations of frequency analysis are shown in Fig. 20.

**Table 18:** Results of frequency analysis for common challenges and barriers (Q11).

Barriers to effective cybersecurity training (Q11)	Frequency
Lack of time or resources	34
Lack of management support or prioritization	29
Insufficiently tailored content to specific roles or departments	12
Training not perceived as relevant or important by employees	11
Lack of time/resources; Insufficiently tailored content; Lack of management support; Training not perceived relevant	9
Lack of management support; Training not perceived relevant	5
Lack of time/resources; Insufficiently tailored content; Training not perceived relevant	2
Lack of time/resources; Insufficiently tailored content	2
Lack of time/resources; Training not perceived relevant	1
Insufficiently tailored content; Lack of management support; Training not perceived relevant	1
Lack of time/resources; Lack of management support	1

**Key observations drawn from the results of frequency analysis:** The conclusions drawn from the frequency analysis to analyze the challenges and barriers that organizations encounter while putting in place efficient cybersecurity training initiatives through Q11 are presented below. The findings present significant insights:

**Barriers to effective training (Q11):** The results of frequency analysis obtained in terms identifying the biggest barriers to implementing effective cybersecurity training in the organization concludes that the most prevalent challenges to establishing efficient cybersecurity training are a lack of time or resources (*n* = 49), as well as a lack of management support or prioritization (*n* = 45). Furthermore, some respondents commented that the content of the training is not appropriately targeted to certain roles or departments (*n* = 26) and that it is not considered to be relevant or important (*n* = 29).

#### Overall conclusion of barriers and challenges

Several key challenges are revealed by analysing the barriers in establishing appropriate cybersecurity training within organisations managing NGS data. The descriptive statistics show significant variation in the barriers that organisations provide. This suggests that while some organisations report many issues, others report fewer barriers. According to this study, the most common challenges are inadequate management support or prioritisation and a lack of time or resources. A smaller but considerable subset of respondents also pointed out that the training material is not adequately adapted to specific roles and few respondents feel that the provided training is irrelevant.

### Incident-driven modifications

The organization’s response to actual cybersecurity issues and their impact on enhancing cybersecurity procedures are evaluated in this section. It aims to specifically identify:

**Incident response and modifications:** This refers to how well an organization modifies and improves its policies or training programs in response to cybersecurity issues. This illustrates the organization’s capacity to take lessons from incidents and apply adjustments to stop them from occurring repeatedly.

**Updates to organizational policies and training programs:** It evaluates whether any occurrences have led to modifications to cybersecurity training resources or programs. This demonstrates the company’s dedication to enhancing its cybersecurity posture on an ongoing basis considering practical experience.

Following question is considered for this section:

**Q12:** Has there been a cybersecurity incident within your organization such as hacking, password stealing, *etc*., that led to changes or enhancements in the training program or policy change?

#### Descriptive statistics

**Figure 21:**
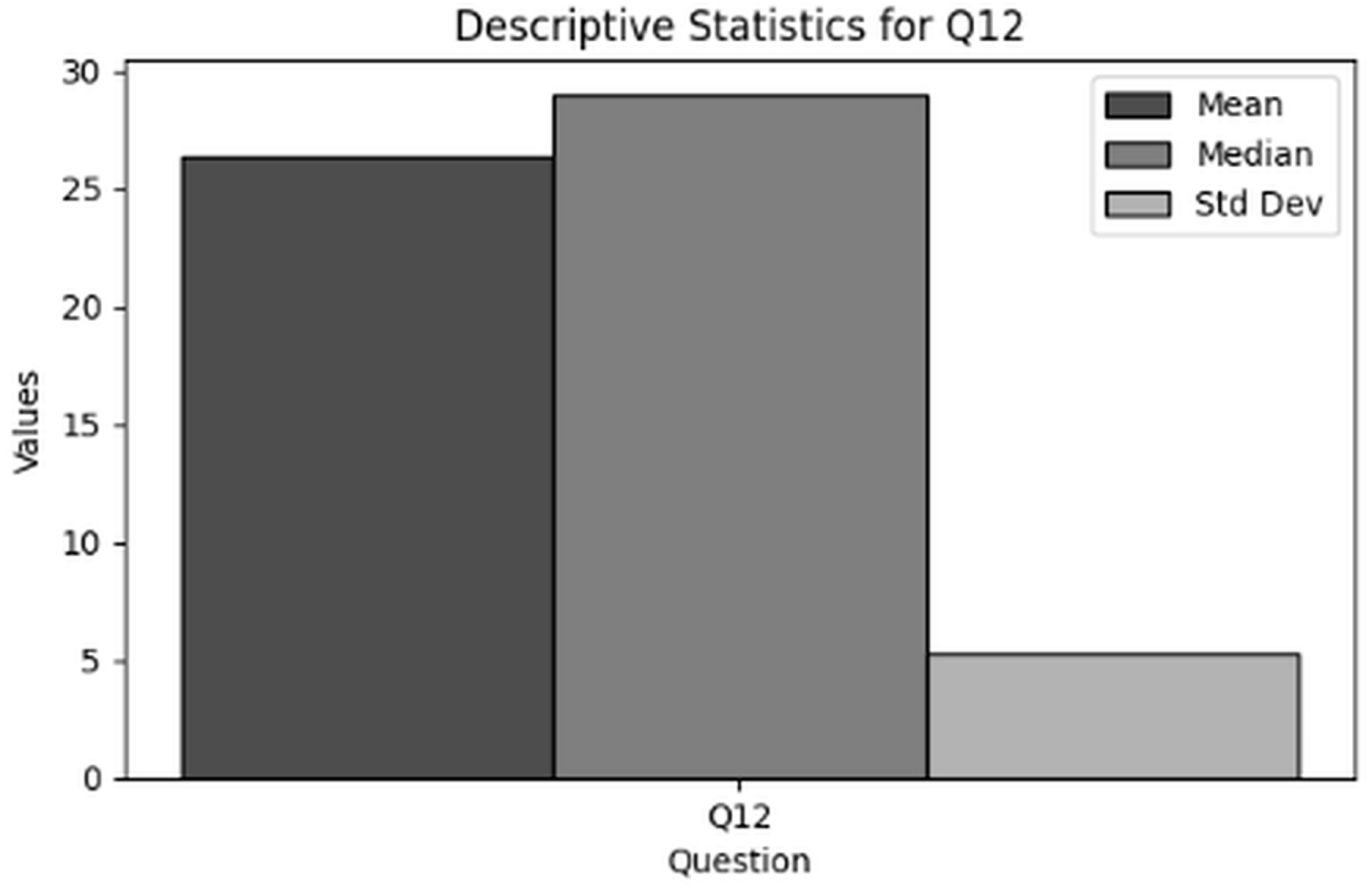
The graphical visualization of descriptive statistics for incident-driven modifications through Q12.

The purpose of descriptive statistics is to summarise the data to obtain an understanding regarding the organization’s response to actual cybersecurity issues and their impact on enhancing cybersecurity procedures working with NGS data. This includes calculating means, medians, and standard deviations as shown in Table 19. For question Q12, the mean, median, and standard deviation are included in the table to provide an overview of the responses’ primary patterns and variations. Figure 21 presents the graphical visualization of the descriptive statistics to summarize the values of mean, median and standard deviations for Q12.

**Table 19:** Results of descriptive statistics for incident-driven modifications in cybersecurity dealing with NGS data through Q12.

Statistics	Q12
Mean	26.43
Median	29.00
Std Dev	5.31

**Key observations drawn from the results of descriptive statistics:** The conclusions drawn from the descriptive statistics to analyze incident driven modifications that organizations encounter while putting in place efficient cybersecurity training initiatives through Q12 are presented below. The findings present significant insights:

**Cybersecurity breaches affecting NGS data (Q12):** The statistical results obtained in terms of identifying cybersecurity incident within the organization such as hacking, password stealing *etc*., that led to changes or enhancements in the training program or policy change concludes that the occurrence of cybersecurity breaches impacting NGS, or other sensitive data is high. A moderate level of variation is also indicated, which indicates a continuous but somewhat variable experience among organizations dealing with NGS data.

#### Frequency analysis

**Figure 22:**
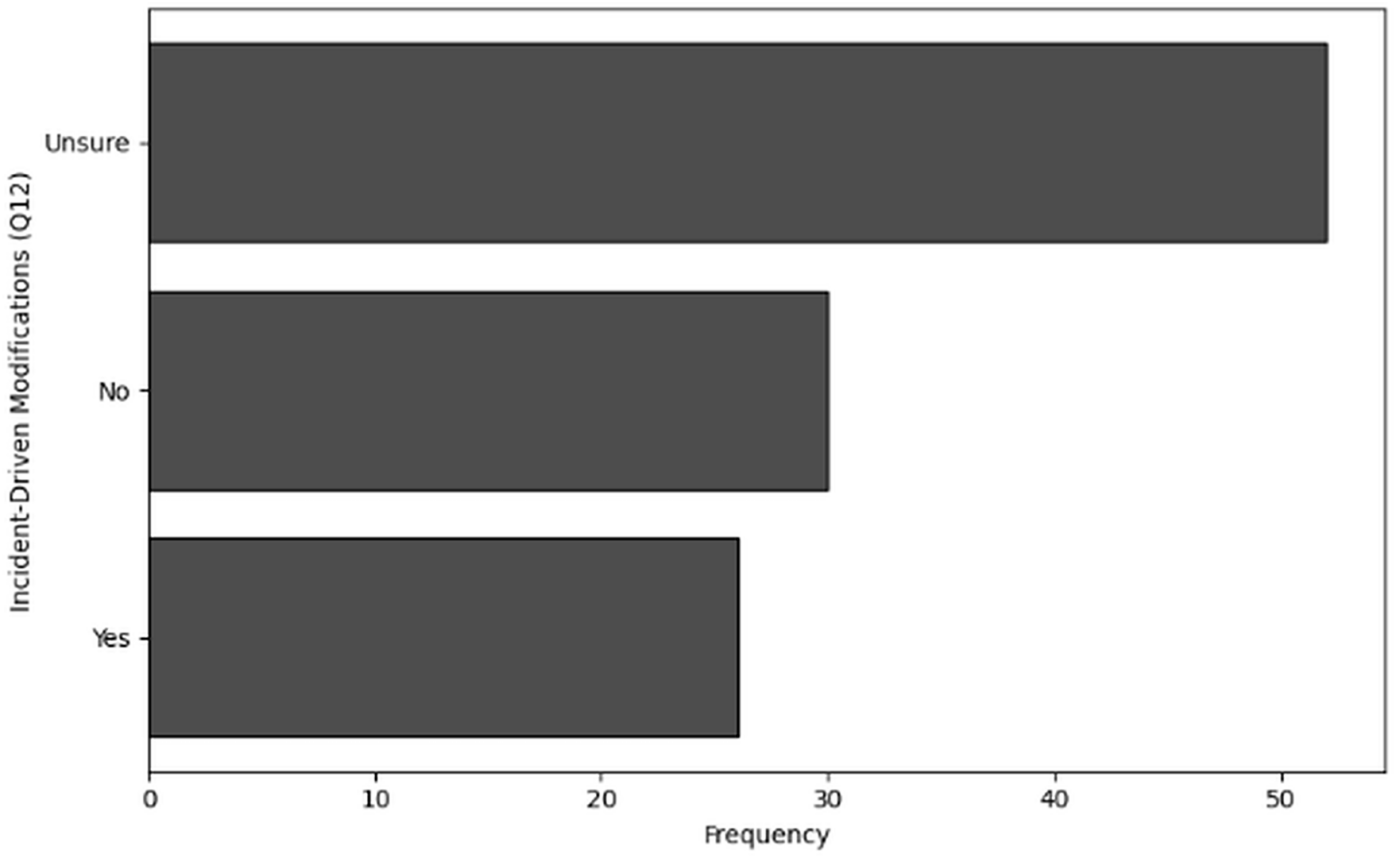
The graphical visualization of frequency analysis for incident-driven modifications through Q12.

The purpose of frequency analysis is to summarize the organization’s response to actual cybersecurity issues and their impact on enhancing cybersecurity procedures working with NGS data by counting the number of inputs. The result of responses to analyze the frequency of user inputs is shown in Table 20. The visual representations of frequency analysis are shown in Fig. 22.

**Table 20:** Results of frequency analysis for incident-driven modifications (Q12).

Responses	Frequency
Unsure	52
No	30
Yes	26

**Key observations drawn from the results of frequency analysis:** The conclusions drawn from the frequency analysis to analyze the incident-driven modifications of cybersecurity in organizations working with NGS are presented below. The findings present significant insights:

**Cybersecurity breaches affecting NGS data (Q12):** The statistical results obtained in terms of identifying cybersecurity incident within the organization such as hacking, password stealing *etc*., that led to changes or enhancements in the training program or policy change concludes that a large percentage of respondents (*n* = 52) are unclear as to whether cybersecurity incidents have resulted in improvements or modifications to policy or training initiatives or not. Only, few (*n* = 26) respondents confirmed that incidences have resulted in modifications. Although some (*n* = 30) reported no such changes.

#### Overall conclusion of incident-driven modifications

According to analysis of incident-driven improvements made in response to cybersecurity breaches in organisations handling NGS data indicate varying degrees of awareness and action. The data obtained reveal heterogeneity between organisations, and the descriptive statistics point to a reasonably high frequency of cybersecurity events resulting in modifications to training programs or policies. The analysis also shows that few respondents reported no changes at all, and a very small percentage of respondents confirmed that cybersecurity incidents had changed training and policies. Many respondents are also unclear as to how much cybersecurity incidents have caused changes in their organisations.

### Organization behavior and security culture

This section evaluates the organization’s overall cybersecurity environment with particular focus on communication methods, policies, and overall culture. Its specific objectives are to:

**Clear and accessible cybersecurity policies:** It evaluates if the company has clear, easily accessible cybersecurity procedures that are applicable to NGS operations and confidential patient data. To ensure adherence to cybersecurity best practices and to guide employee behaviour, clear policies are necessary.

**Methods of communicating about cybersecurity issues:** It analyses the channels and techniques that the organisation employs to spread knowledge on cybersecurity matters. It is essential to have strategies for communication that work to inform users about best practices, policy modifications, and possible threats. The investigation evaluates various communication strategies’ effectiveness and impact.

**Overall culture of cybersecurity:** It analyses the organization’s general cybersecurity-related attitudes, values, and behaviours. It addresses how seriously everyone is taking cybersecurity, from executives to front-line staff. An organisation that prioritises and successfully manages cybersecurity risks is likely to have a strong cybersecurity culture.

**Frequency of cybersecurity discussions in meetings:** It evaluates how frequently discussions about cybersecurity are held during meetings or in other communications inside the organisation. Frequent cybersecurity discussions can be a sign of a proactive strategy for controlling cybersecurity risks and maintaining the topic at the top of the organisational priority list.

Following questions are considered for this section:

**Q13:** Does your organization have clear and accessible cybersecurity policies specific to NGS operations or sensitive healthcare data such as DNA sequencing data?

**Q14:** What methods does your organization use to communicate about cybersecurity issues (Select all that apply)?

**Q15:** How would you describe the overall culture of cybersecurity within your organization?

**Q16:** In the last year, how often has cybersecurity been discussed in your meetings or other organizational communications?

#### Descriptive statistics

**Figure 23:**
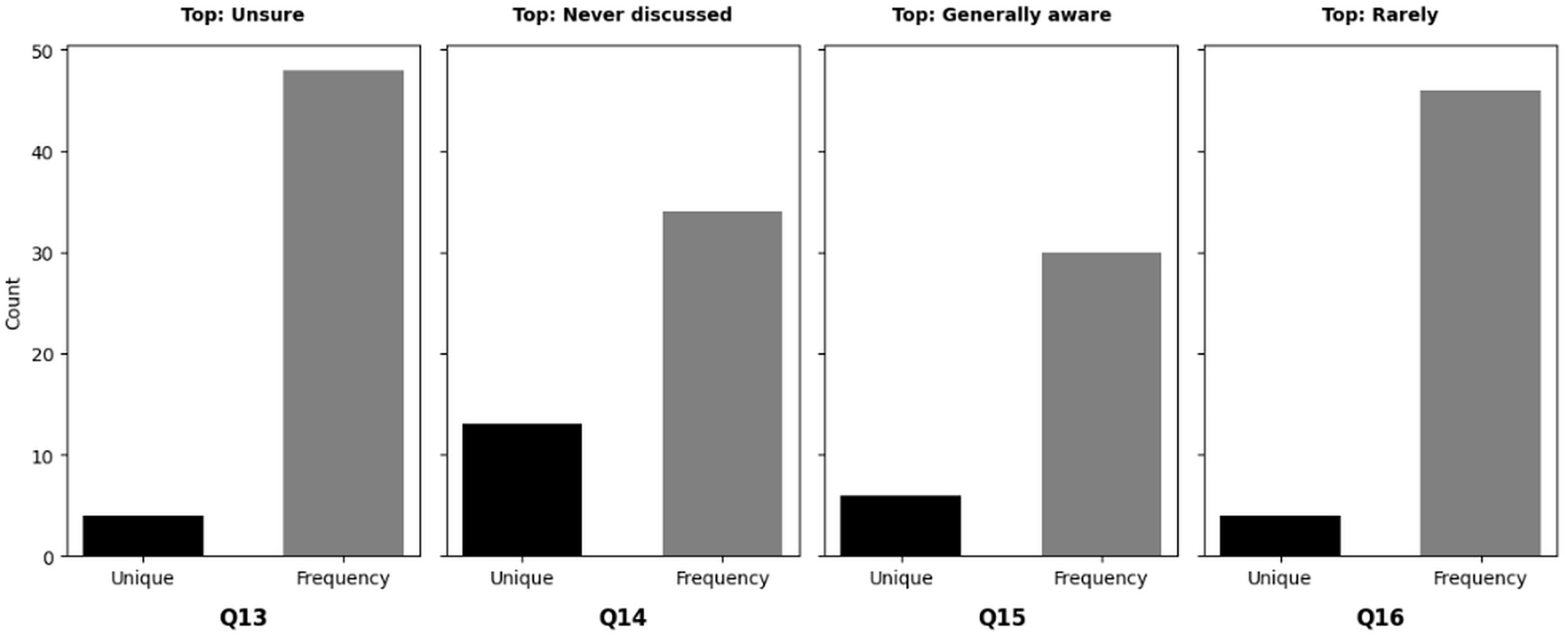
The graphical visualization of descriptive statistics for organization behaviour and security culture through Q13–Q16.

The purpose of descriptive statistics is to summarise the data to obtain an understanding regarding the organization’s overall cybersecurity environment with particular focus on communication methods, policies, and overall culture with NGS data. This includes calculating means, medians, and standard deviations as shown in Table 21. For questions Q13–Q16, the mean, median, and standard deviation are included in the table to provide an overview of the responses’ primary patterns and variations. Figure 23 presents the graphical visualization of the descriptive statistics to summarize the values of mean, median and standard deviations for Q13–Q16.

**Table 21:** Results of descriptive statistics for organization behaviour and security culture through Q13–Q16.

Statistics	Q13	Q14	Q15	Q16
Unique	4	13	6	4
Top	Unsure	Never discussed	Uncertain/Not applicable	Rarely
Frequency	48	34	30	46

**Key observations drawn from the results of descriptive statistics:** The conclusions drawn from the descriptive statistics to analyze the organization’s overall cybersecurity environment with particular focus on communication methods, policies, and overall culture with NGS data through Q13–Q16 are presented below. The findings present significant insights:

**Clear and accessible cybersecurity policies (13):** Most respondents have chosen “Unsure” which indicates that most of the users are unsure regarding the organization’s accessible cybersecurity policies designed for sensitive NGS data. This highlights the potential of a gap in the policies’ awareness and accessibility by indicating that either the policies are not clearly communicated to the organization, or they are not fully known by them.

**Methods of communicating about cybersecurity issues (Q14):** According to a significant number of respondents who selected “Never discussed” as their top choice indicates that cybersecurity issues are never mentioned in their organisation. This lack of communication creates problems since it suggests that employees are not properly updated on cybersecurity practices, rules, involved risks, and cybersecurity i not be prioritised in the organisational culture.

**Overall culture of cybersecurity (Q15):** According to a significant number of respondents who selected “Generally aware” as their top choice indicates an unclear cybersecurity culture in which users are unaware of the seriousness with which cybersecurity is taken at all organisational levels. It also implies that cybersecurity is not fully incorporated into the organization’s daily operations and policies.

**Frequency of cybersecurity discussions in meetings (Q16):** According to a significant number of respondents, cybersecurity is not frequently covered in meetings or other organisational communications (top response: “rarely”). The fact that this topic is rarely discussed indicates to a reactive rather than proactive approach to cybersecurity, where challenges are usually addressed as they occur rather than being a regular and significant concern.

#### Frequency analysis

**Figure 24:**
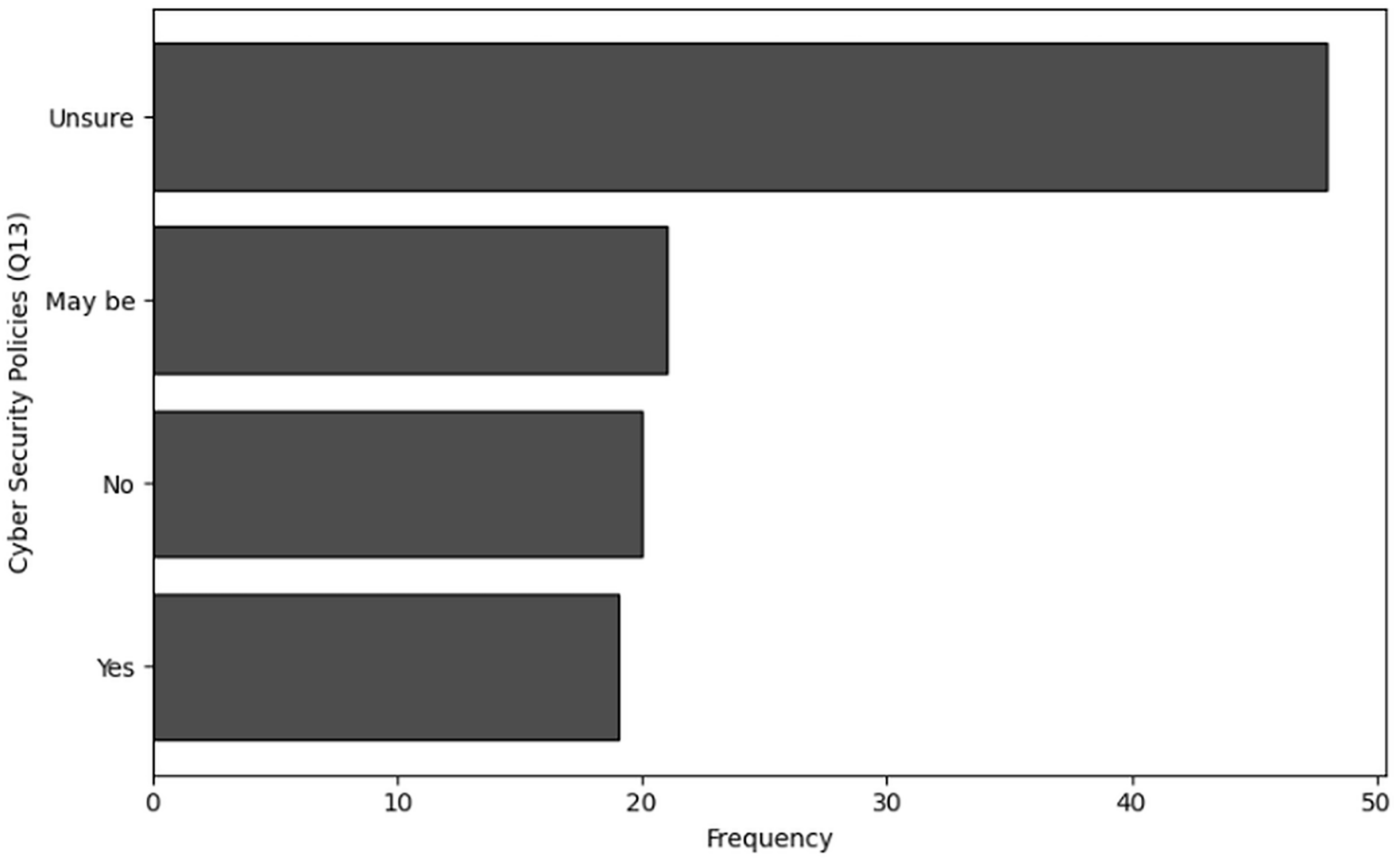
The graphical visualization of frequency analysis for organization’s cybersecurity policies specific to NGS through Q13.

**Figure 25:**
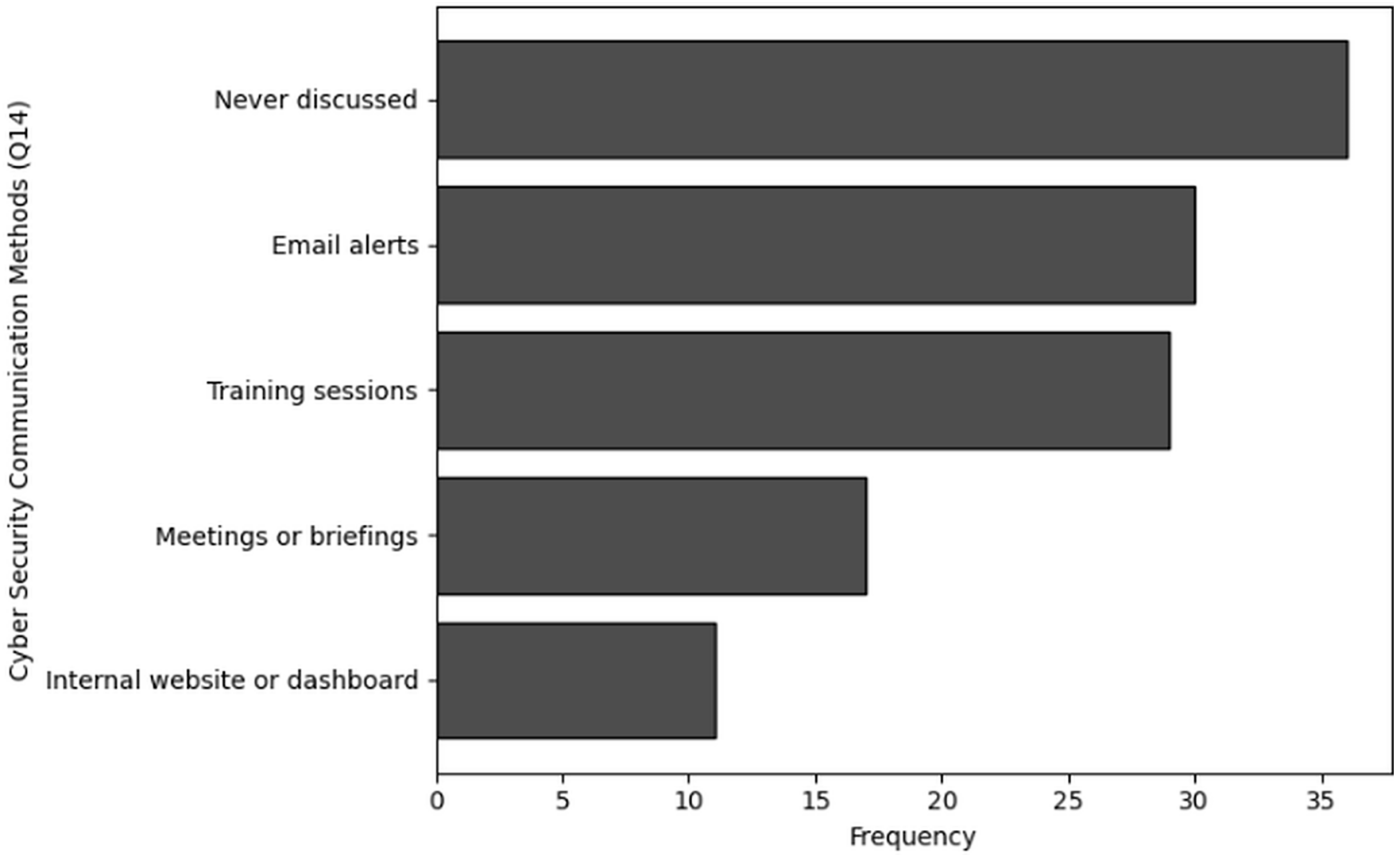
The graphical visualization of frequency analysis for organization’s cybersecurity communication methods specific to NGS through Q14.

**Figure 26:**
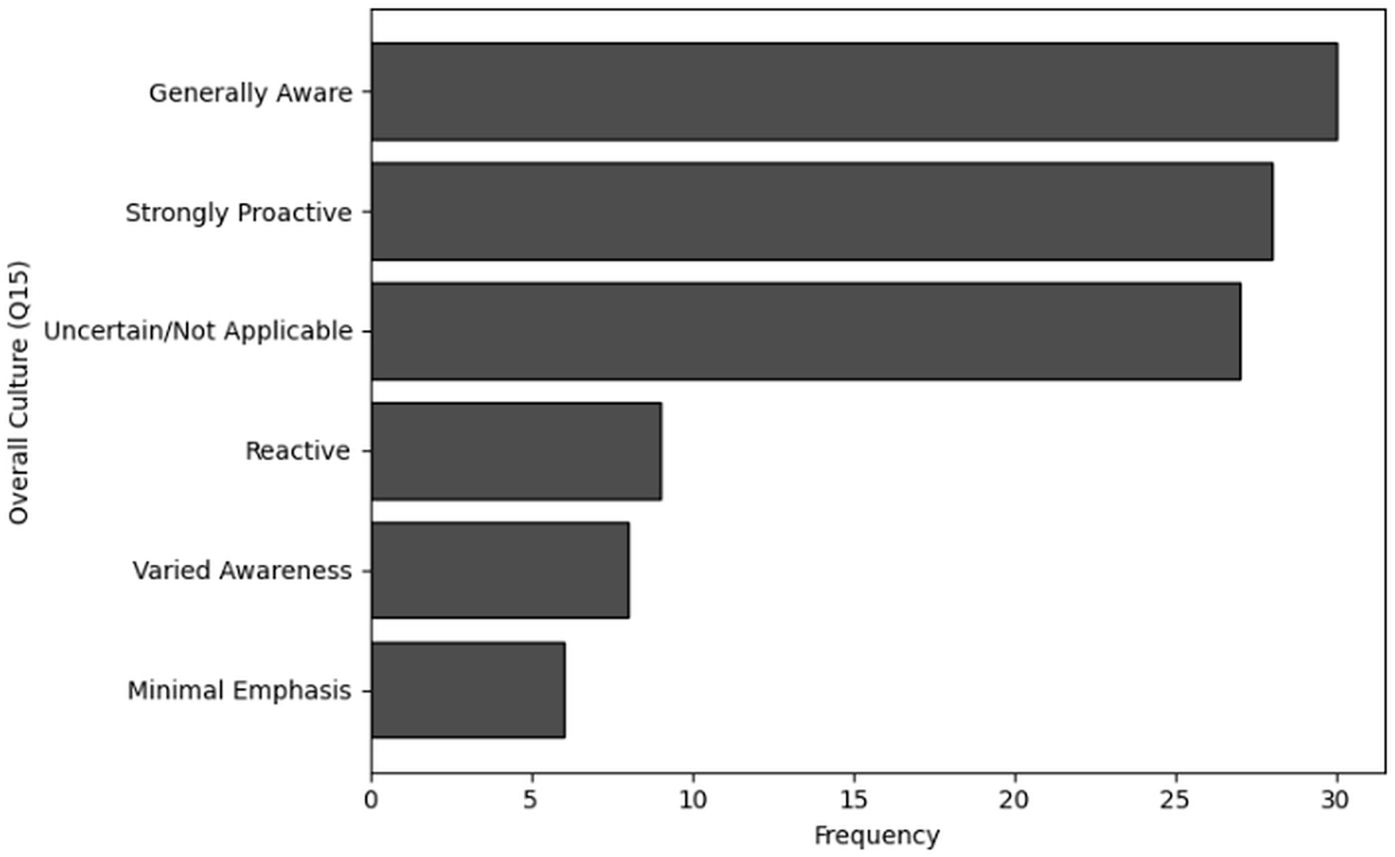
The graphical visualization of frequency analysis for overall culture of cybersecurity in organization through Q15.

**Figure 27:**
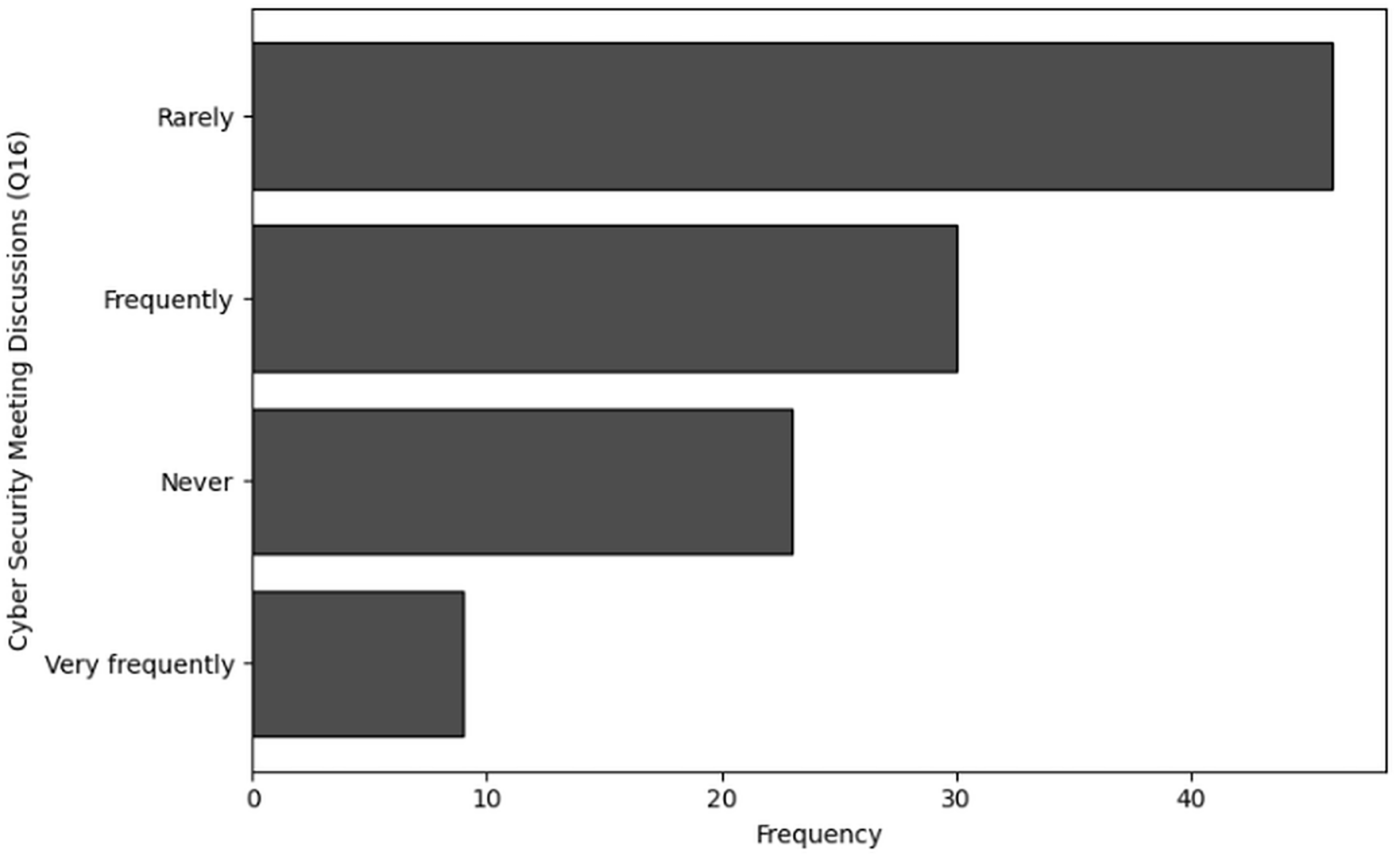
The graphical visualization of frequency analysis for organization’s cybersecurity meeting discussions specific to NGS through Q16.

The purpose of frequency analysis is to summarize the organization’s response to the organization’s overall cybersecurity environment with particular focus on communication methods, policies, and overall culture with NGS data by counting the number of inputs. The results of responses to analyze the frequency of user inputs for clear and accessible cybersecurity policies (Q13), methods of communicating about cybersecurity issues (Q14), overall culture of cybersecurity (Q15) and frequency of cybersecurity discussions in meetings (Q16) are shown in Tables 22, 23, 24, and 25 respectively. The visual representations of frequency analysis for clear and accessible cybersecurity policies (Q13), methods of communicating about cybersecurity issues (Q14), overall culture of cybersecurity (Q15) and frequency of cybersecurity discussions in meetings (Q16) are shown in Figs. 24, 25, and 26, and 27, respectively.

**Table 22:** Results of frequency analysis for organization’s cybersecurity policies specific to NGS through Q13.

Cybersecurity policies (Q13)	Frequency
Unsure	48
No	21
May be	20
Yes	19

**Table 23:** Results of frequency analysis for Organization’s cybersecurity communication methods specific to NGS through Q14.

Cybersecurity communication methods (Q14)	Frequency
Never discussed	36
Email alerts	30
Training sessions	29
Meetings or briefings	17
Internal website or dashboard	11

**Table 24:** Results of frequency analysis for overall culture of cybersecurity in organization through Q15.

Overall culture (Q15)	Frequency
Generally aware	30
Strongly proactive	28
Uncertain/Not applicable	27
Reactive	9
Varied awareness	8
Minimal emphasis	6

**Table 25:** Results of frequency analysis for organization’s cybersecurity meeting discussions specific to NGS through Q16.

Cybersecurity meeting discussions (Q16)	Frequency
Rarely	46
Frequently	30
Never	23
Very frequently	9

**Key observations drawn from the results of frequency analysis:** The conclusions drawn from the frequency analysis to analyze the organization’s response to the organization’s overall cybersecurity environment with particular focus on communication methods, policies, and overall culture with NGS data are presented below. The findings present significant insights:

**Clear and accessible cybersecurity policies (Q13):** The most common response to the question concerning the accessibility and clarity of cybersecurity policy was given by 48 out of 108 respondents, who expressed uncertainty. According to 21 respondents, their company lacks accessible and transparent procedures. A total of 19 respondents verified that their business has clear and accessible cybersecurity rules, indicating a need for enhanced communication and policy enforcement. A total of 20 respondents were confused about the policies’ clarity and accessibility.

**Methods of communicating about cybersecurity issues (Q14):** The most common response, given by 36 out of 108 respondents, regarding communication strategies for cybersecurity issues consisted of these issues are never raised. Cybersecurity-related issues are conveyed *via* email alerts, according to 30 respondents. Meetings were reported to be carried out by 17 respondents, while 29 respondents mentioned conduction of training sessions. The usage of an internal website or dashboard was only mentioned by 11 respondents. Some participants observed that various approaches to communication are used in different organizations, such as internal websites, email alerts, meetings, briefings, and training sessions.

**Overall culture of cybersecurity (Q15):** Most of the respondents (*i.e*., 30 out of the 108) generally understood the value of cybersecurity, with some policies and training supporting this knowledge. Within their companies, 28 respondents reported a very proactive approach to cybersecurity that included consistent training, open communication, and efficient procedures that actively avert problems. Furthermore, 27 responses indicated that they were either unaware of the cybersecurity culture or did not feel it applied to their role or experience. Additionally, nine respondents outlined a reactive strategy in which cybersecurity is prioritized only after an incident occurs and regulations are not strictly followed until an incident occurs. Nonetheless, eight participants observed notable disparities in cybersecurity strategies and awareness among various teams or departments. Finally, six respondents claimed that there was little focus on cybersecurity, citing infrequent training, unclear or weak policies, and a lack of proactive effort to prevent risks.

**Frequency of cybersecurity discussions in meetings (Q16):** The most common response, given by 46 out of 108 respondents, was that cybersecurity discussions in meetings are rare. While 23 respondents reported that cybersecurity is never discussed in their meetings. However, 30 respondents indicated that the topic is often discussed. There is a need for more frequent integration of cybersecurity concerns into organizational communications, since only nine respondents indicated that cybersecurity is mentioned very regularly.

#### Cluster analysis

**Figure 28:**
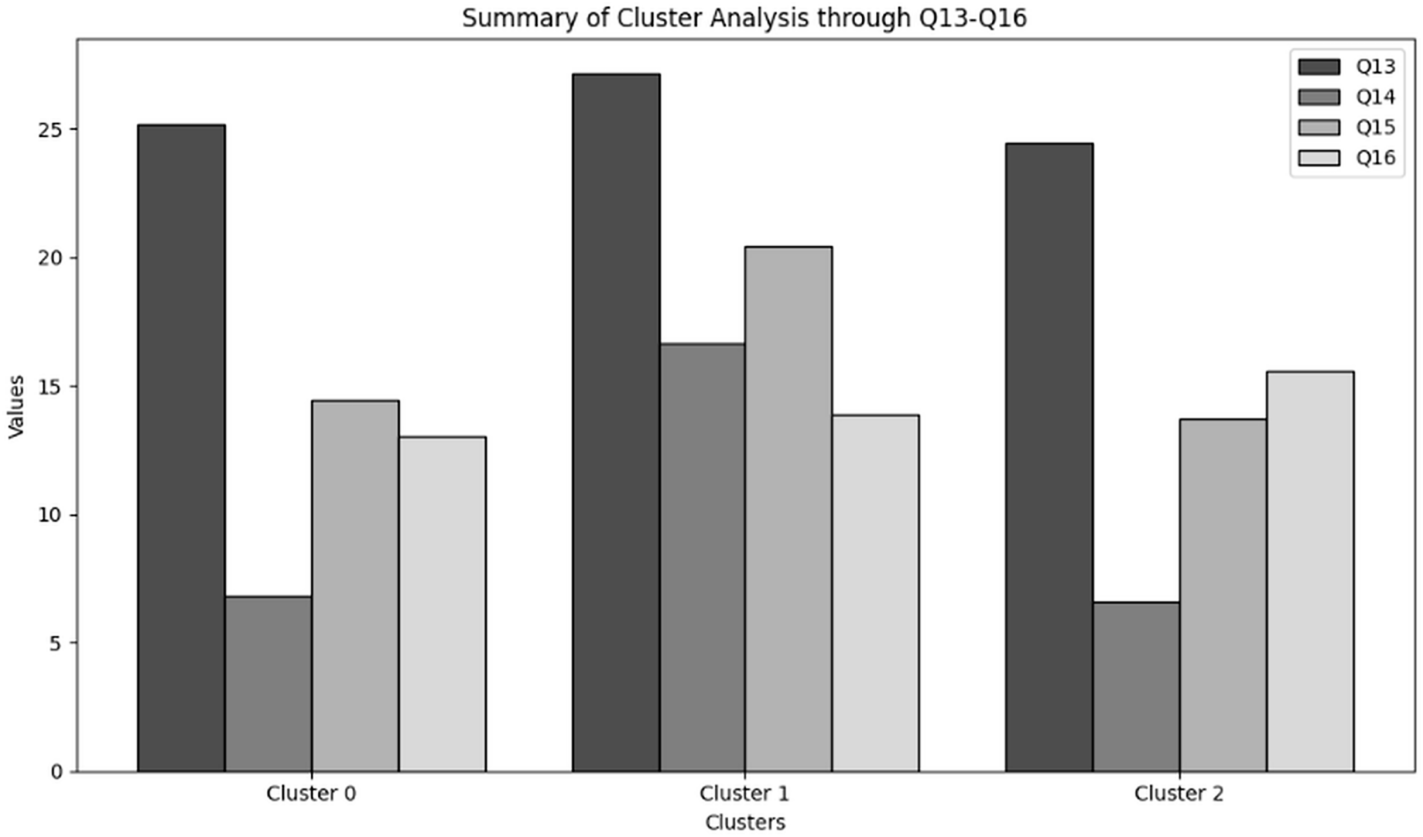
Visual representation of cluster analysis for organizational behavior and cybersecurity culture variables through Q13–Q16.

The purpose of cluster analysis is to summarize responses related to group organizations based on similarities in their cybersecurity culture, in order to identify distinct clusters working with NGS by using all the relevant questions. K-means clustering technique is used to analyze Q13–Q16. A well justified choice of three clusters is taken for the results of cluster analysis. This decision is consistent with the conceptual framework that validates hypothesis H1. It is possible to directly compare variables like training frequency, employee confidence, and policy update processes across robust, moderate, and emergent profiles by dividing organizations into three groups. The random state is also set to 42. It confirms the clustering findings’ consistency, enabling reliable comparisons and verification between several runs. Furthermore, all variables were normalized before clustering to make sure that scale variations wouldn’t skew the K-means algorithm’s standard distance computations. The cluster summary is presented in Table 26. The visual representation of cluster analysis is shown in Fig. 28.

**Table 26:** Summary of cluster analysis through Q13–Q16.

Cluster	Q13	Q14	Q15	Q16
0	25.19	6.84	14.45	13.05
1	27.13	16.63	20.44	13.88
2	24.42	6.61	13.71	15.57

**Key observations drawn from the results of cluster analysis:** The key observations drawn cluster analysis is to summarize responses related to group organizations based on similarities in their cybersecurity culture to identify distinct clusters working with NGS are presented below. The findings present significant insights to find distinct patterns through clusters:

Based on their cybersecurity procedures related to NGS data, three different organisational patterns are brought into focus by the cluster analysis. Each cluster is representing a different set of organizations based on the organizational behaviour and cybersecurity culture.

**Cluster 0** organisations have strong cybersecurity practices. These practices include transparent policies, effective communication channels, a supportive cybersecurity culture, and frequent discussions on cybersecurity-related topics. Organisations in this cluster are perhaps more established and proactive when it comes to cybersecurity.

**Cluster 1** organisations have moderate cybersecurity procedures, with some established guidelines and channels of communication. Although, they do not have as strong of a cybersecurity culture overall, and cybersecurity conversations are infrequent. These organisations need to improve their cybersecurity involvement and policies because of the lack of awareness.

**Cluster 2** organisations have a diverse profile. They struggle with clear policies and efficient communication techniques, but they have effective cybersecurity cultures and regular cybersecurity discussions. To complement their strong cybersecurity culture, these organisations need to broaden their communication techniques and make their policies more easily accessible.

Three unique organisational features are highlighted by the cluster analysis related to NGS data cybersecurity practices. The organisations in Cluster 0 have strong cybersecurity policies, which include well-defined standards, efficient means of communication, and a proactive culture. Furthermore, organisations in Cluster 1 with moderate practices could need to improve their cybersecurity culture and frequency of communication. Additionally, the organisations in Cluster 2 have robust cybersecurity cultures that participate in regular dialogues, but they also struggle with effective communication and clear policies. Therefore, for more effective handling of sensitive NGS data, these insights can assist organisations in identifying areas where their cybersecurity strategies (particularly those in Clusters 1 and 2), need to be improved.

#### Overall conclusion of organization behavior and security culture

According to the analysis of the organization’s overall cybersecurity culture that focuses on communication strategies, policies, and culture there are significant discrepancies in knowledge, communication, and proactive involvement with cybersecurity practices. It shows that there is a lack of clear communication and awareness of cybersecurity regulations associated with NGS operations inside the organization, as a significant portion of respondents answered “Unsure” regarding their presence and accessibility. Additionally, the top response for communication methods highlights the fact that cybersecurity issues are “Never discussed” in many organizations, implying that users are not effectively informed about cybersecurity risks, practices, and policies. Many respondents interpreted the overall cybersecurity culture within organization as uncertain, which reflects a weakly defined cybersecurity culture that is not fully incorporated into day-to-day activities. Furthermore, there is a rare discussion of cybersecurity in meetings and organizational communications, indicating that addressing cybersecurity risks occurs only reactively rather than proactively. To effectively manage the risks connected with NGS data, the findings highlight the necessity for organizations to increase communication and training efforts, strengthen their cybersecurity culture, and make cybersecurity policies more visible and accessible.

### User knowledge and awareness of cybersecurity

This section evaluates users’ knowledge and awareness of NGS technology-specific cybersecurity concerns and best practices. It seeks to specifically identify:

**Awareness of cybersecurity:** It analyses the degree to which users are aware of the cybersecurity threats connected to NGS technologies. To guarantee that users take the appropriate safety measures to protect sensitive genomic data from potential attacks, this understanding is required.

**Understanding the importance of securing genomic data:** It analyses the degree to which users understand the need of protecting genetic data security to protect patient confidentiality and privacy. Comprehending this significance serves to emphasise the necessity of strict cybersecurity protocols while managing such confidential data.

**Familiarity with cybersecurity threats:** It analyses the users’ awareness of common cybersecurity risks and weaknesses that may affect NGS data is measured by their familiarity with cybersecurity threats. This includes being aware of the several types of cyberattacks that can occur, including ransomware, phishing, data breaches, unauthorised access, and targeted attacks on genomic data (Cameron et al., 2025, 2024). Users are more equipped to recognise and address possible security events when they are familiar with these threats.

**Awareness of bio-cybersecurity:** It analyses the users’ knowledge of bio-cybersecurity: Knowledge of the idea of bio-cybersecurity, which includes safeguarding biological systems and data against cyberattacks. The term highlights the connection between biological data protection and cybersecurity and is especially relevant to individuals who work with genomic and other biological data.

Following questions are considered for this section:

**Q17:** Are you aware of the potential cybersecurity risks associated with next-generation sequencing (NGS) technologies?

**Q18:** Do you understand the importance of securing genomic data generated by NGS technologies to protect patient privacy and confidentiality?

**Q19:** Are you familiar with common cybersecurity threats and vulnerabilities that can affect NGS data, such as data breaches, ransomware attacks, unauthorized access, phishing attacks, targeted attacks on genomic data, or manipulation of sequencing results?

**Q20:** Have you heard about the term bio-cybersecurity?

#### Descriptive statistics

**Figure 29:**
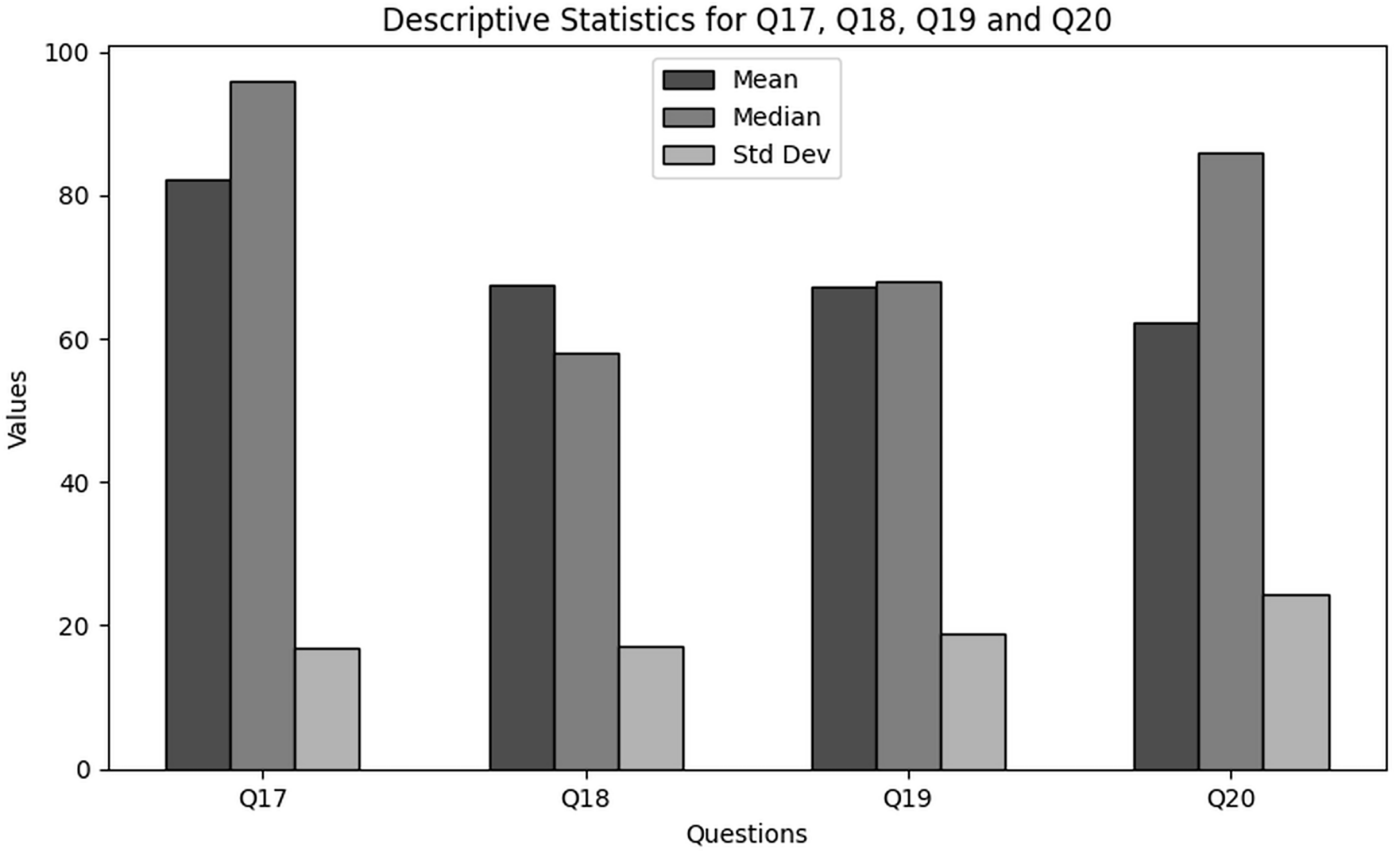
The graphical visualization of descriptive statistics for user knowledge and awareness of cybersecurity through Q17–Q20.

The purpose of descriptive statistics is to summarise the data to obtain an understanding regarding the users’ knowledge and comprehension of NGS technology-specific cybersecurity concerns and best practices. This includes calculating means, medians, and standard deviations as shown in Table 27. For question Q17–Q20, the mean, median, and standard deviation are included in the table to provide an overview of the user knowledge and awareness of cybersecurity. Figure 29 presents the graphical visualization of the descriptive statistics to summarize the values of mean, median and standard deviations for Q17–Q20.

**Table 27:** Results of descriptive statistics for user knowledge and awareness of cybersecurity dealing with NGS data through Q17–Q20.

Statistics	Q17	Q18	Q19	Q20
Mean	82.16	67.37	67.20	62.19
Median	96.00	58.00	68.00	86.00
Std	16.81	17.21	18.87	24.45

**Key observations drawn from the results of descriptive statistics:** The conclusions drawn from the descriptive statistics to analyze the evaluates users’ knowledge and comprehension of NGS technology-specific cybersecurity concerns and best practices through Q17–Q20 are presented below. The findings present significant insights:

**Awareness of cybersecurity risks (Q17):** Considering the high mean and median values, it concludes that users are generally well-aware of the cybersecurity concerns connected to NGS technologies. The proximity of the mean and median indicates that respondents’ levels of awareness generally remain constant. The comparatively low standard deviation suggests that most users’ awareness levels are comparable and varied slowly, suggesting that most respondents are aware of cybersecurity threats.

**Understanding the importance of securing genomic data (Q18):** The results of descriptive statistics highlight the relatively high level of understanding. However the slightly lower median value as compared to the mean, suggesting a distribution where some users’ knowledge of the significance of protecting genetic data is likewise strong. While few respondents does not fully understand the significance of securing genomic data.

**Familiarity with common cybersecurity threats (Q19):** The results of descriptive statistics show a moderate level of expertise with common cybersecurity threats and vulnerabilities that may have an impact on NGS data. This implies that although users are aware, they may do better in terms of developing a detailed understanding of threats. Additionally, the moderate standard deviation indicates some variation in respondents’ awareness with cybersecurity threats, suggesting that while some users may be well-informed, others may require additional exposure to or education about these risks.

**Awareness of bio-cybersecurity (Q20):** According to the statistical results, many of respondents are not able to completely understand the idea of bio-cybersecurity. Although it is not universal, the higher median shows that a significant number of respondents have an adequate level of awareness. Additionally, the higher standard deviation suggests significant variation in awareness levels. This suggests that not all users have a consistent understanding of the concept of bio-cybersecurity, emphasizing the necessity for focused training efforts.

#### Frequency analysis

**Figure 30:**
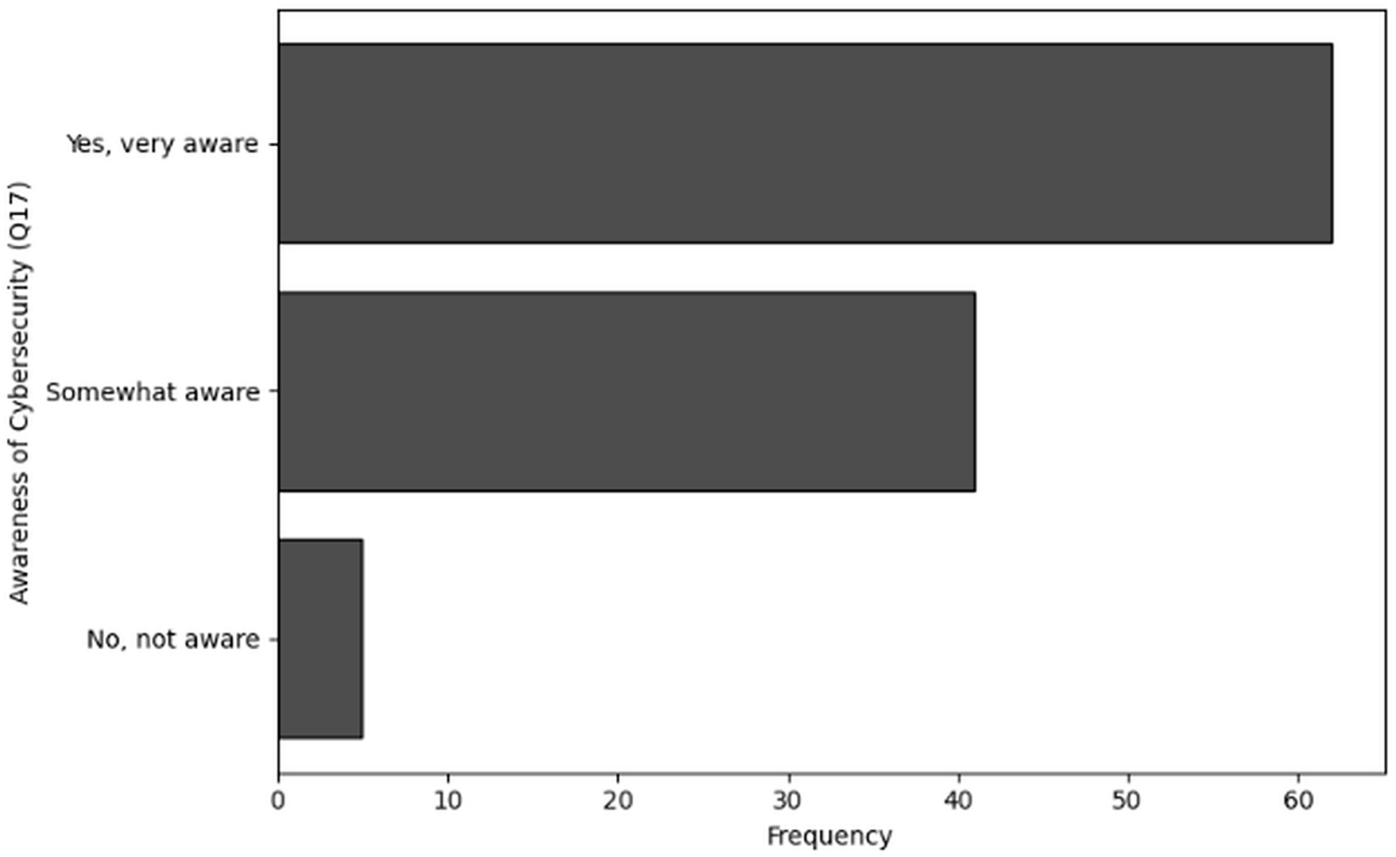
The graphical visualization of frequency analysis for user awareness of cybersecurity through Q17.

**Figure 31:**
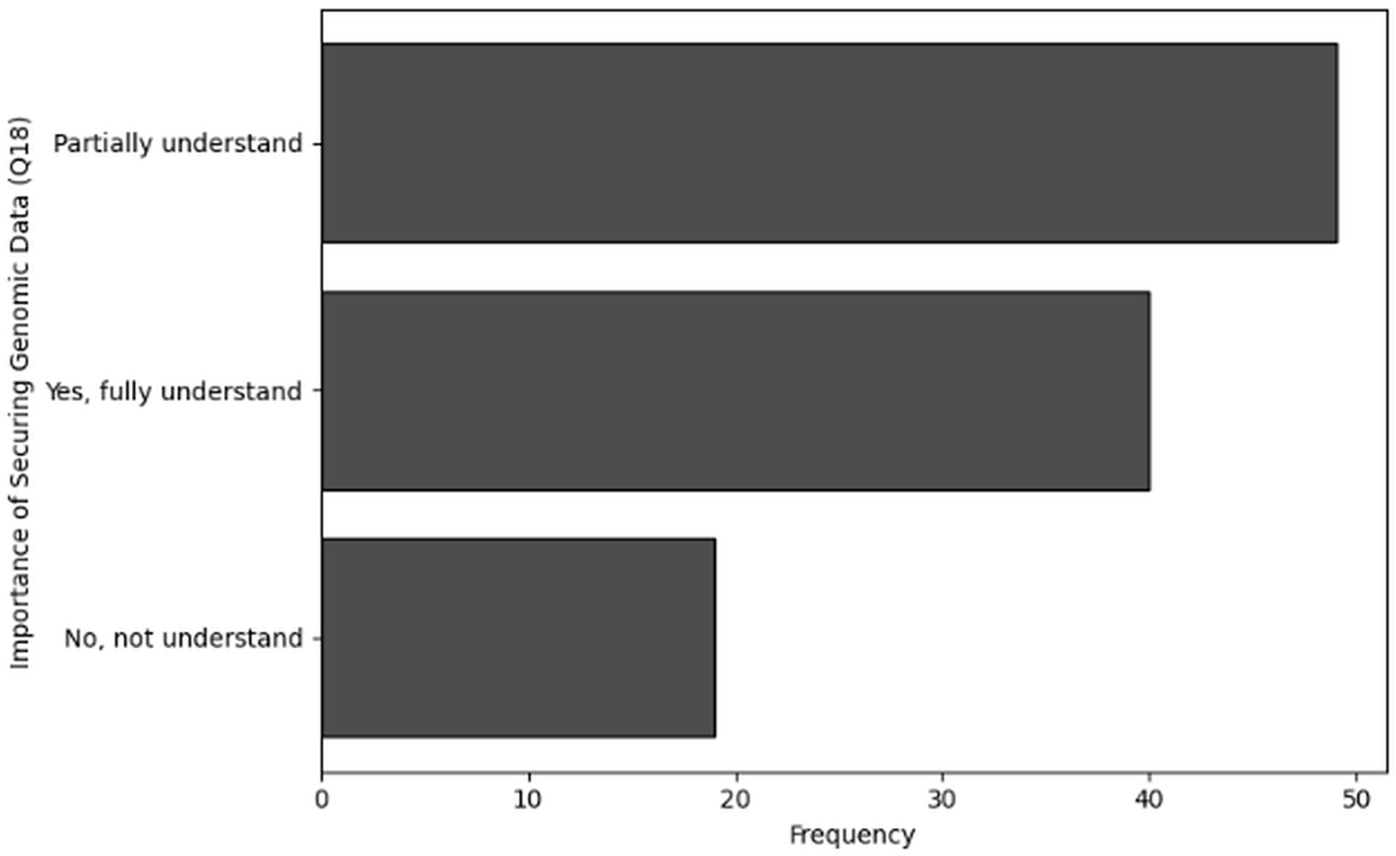
The graphical visualization of frequency analysis for importance of securing genomic data through Q18.

**Figure 32:**
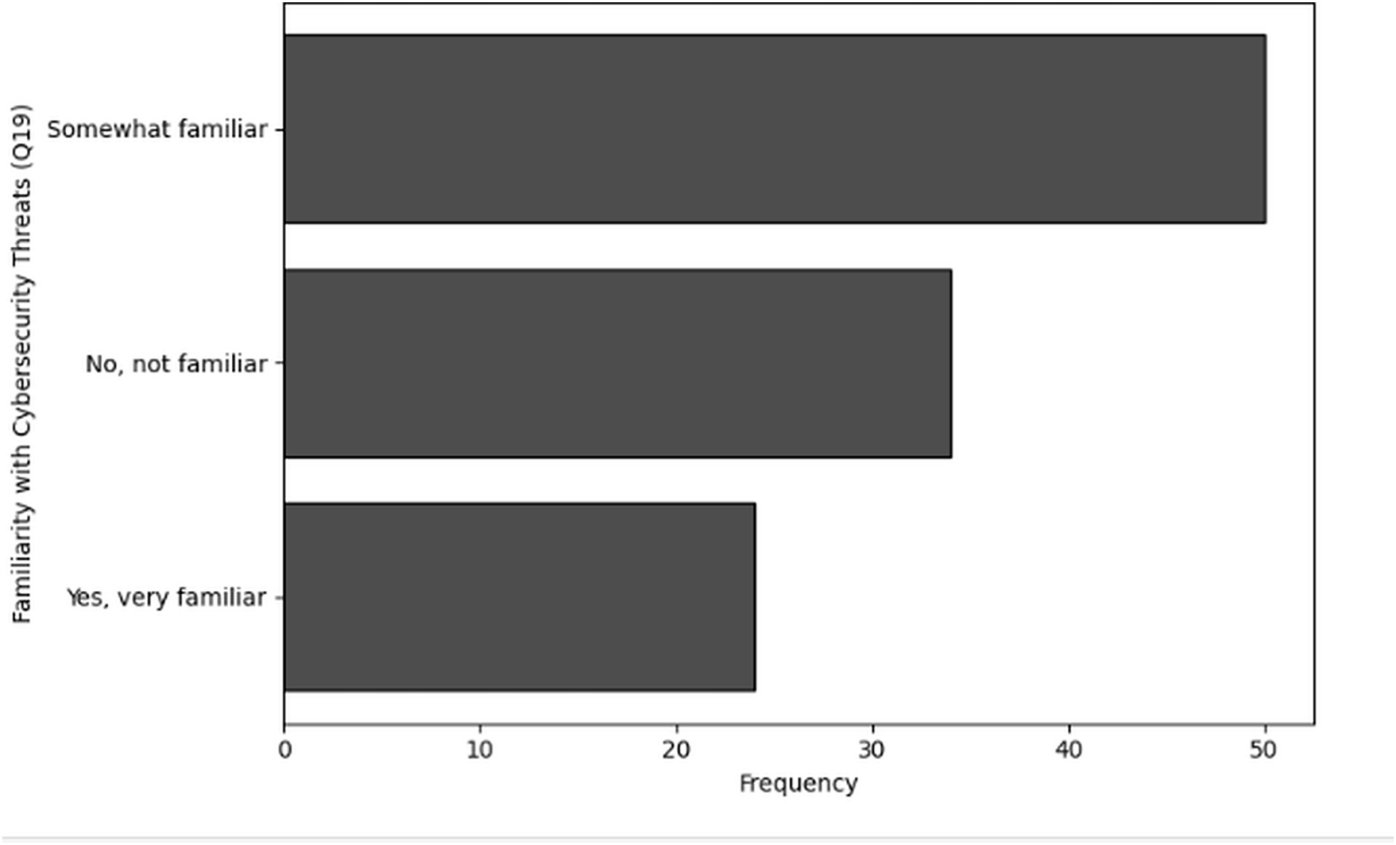
The graphical visualization of frequency analysis for user familiarity with cybersecurity threats through Q19.

**Figure 33:**
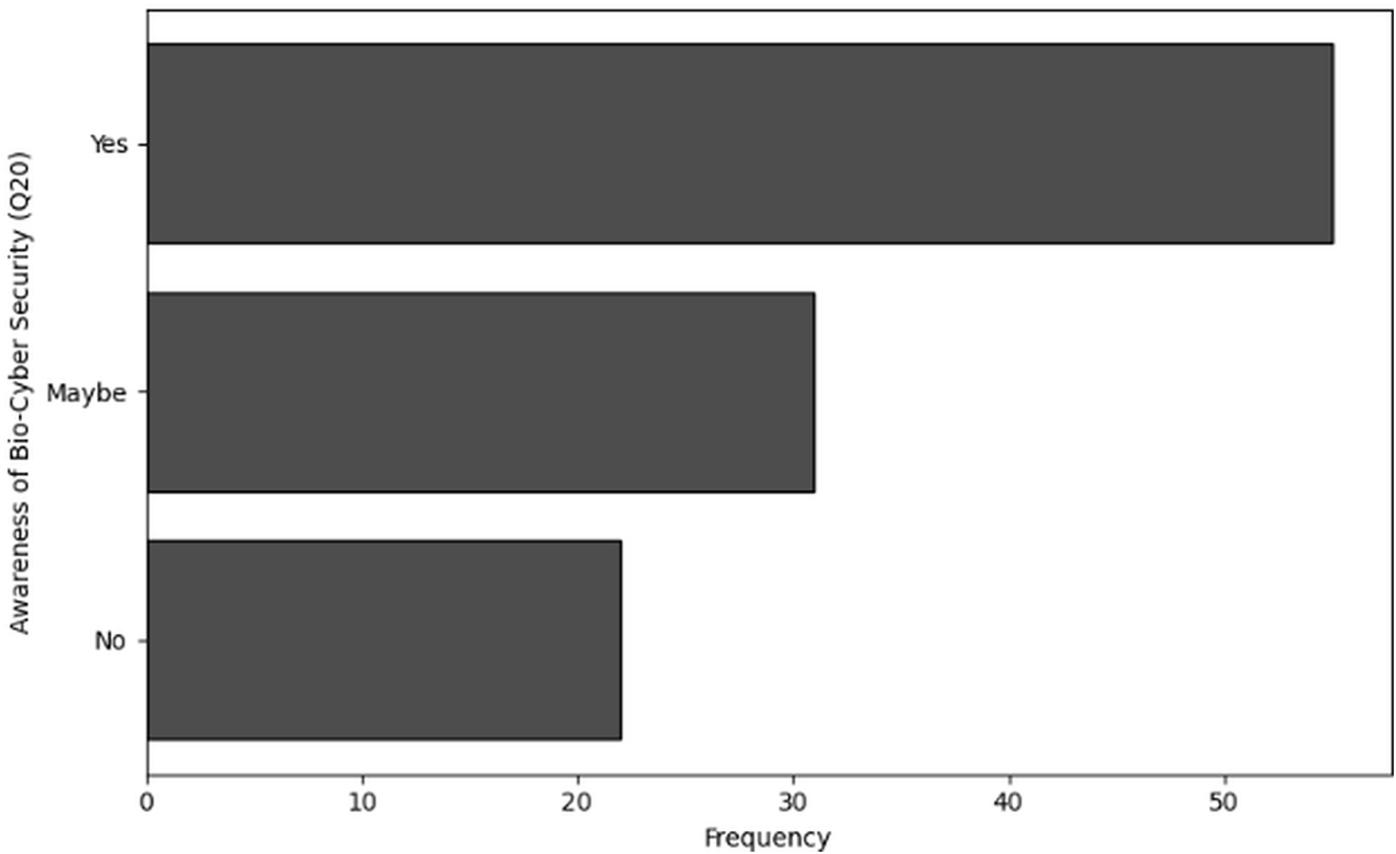
The graphical visualization of frequency analysis for user awareness of bio-cybersecurity through Q20.

The purpose of frequency analysis is to summarize the users’ knowledge and comprehension of NGS technology-specific cybersecurity concerns and best practices by counting the number of inputs. The results of responses to analyze the frequency of user inputs for awareness of cybersecurity risks (17), understanding the importance of securing genomic data (Q18), familiarity with common cybersecurity threats (Q19), and awareness of bio-cybersecurity (Q20) are shown in Tables 28, 29, 30 and 31. The visual representations of frequency analysis for Q17, Q18, Q19 and Q20 are shown in Figs. 30, 31, 32 and 33, respectively.

**Table 28:** Results of frequency analysis for user awareness of cybersecurity through Q17.

Awareness of cybersecurity (Q17)	Frequency
Yes, very aware	62
Somewhat aware	41
No, not aware	5

**Table 29:** Results of frequency analysis for importance of securing genomic data through Q18.

Importance of securing genomic data (Q18)	Frequency
Partially understand	49
Yes, fully understand	40
No, not understand	19

**Table 30:** Results of descriptive statistics for user familiarity with cybersecurity threats through Q19.

Familiarity with cybersecurity threats (Q19)	Frequency
Somewhat familiar	50
No, not familiar	34
Yes, very familiar	24

**Table 31:** Results of frequency analysis for user awareness of cybersecurity through Q20.

Awareness of bio-cybersecurity (Q20)	Frequency
Yes	55
Maybe	31
No	22

**Key observations drawn from the results of cluster analysis:** The conclusions drawn from the frequency analysis to analyze the user knowledge and awareness of cybersecurity working with NGS are presented below. The findings present significant insights:

**Awareness of cybersecurity risks (Q17):** Most of the respondents (*n* = 62) answered “Yes, very aware” which indicates that they are aware of the cybersecurity threats related to NGS technologies. This high degree of awareness is a good sign of users’ level of understanding because it implies that they are knowledgeable about the possible cybersecurity risks unique to NGS technologies. However, many users are somewhat aware. This high count indicates that the users have some knowledge related to cybersecurity in NGS but they are still not confident and there is a lack of understanding while dealing with NGS. Furthermore, only five respondents are not aware regarding cybersecurity which indicates some segment of NGS users need detailed guidance regarding cybersecurity awareness.

**Understanding the importance of securing genomic data (Q18):** Based on the collected data, most respondents either fully (*n* = 40) or somewhat (*n* = 49) understand the relevance of securing genetic data. However, a significant proportion of respondents do not have a clear understanding at all. This suggests that additional training and emphasis on the essential importance of genomic data security is required.

**Familiarity with common cybersecurity threats (Q19):** The responses reflect a wide range of awareness with usual cybersecurity threats. Although most are only somewhat familiar (*n* = 50), many are not familiar at all (34), indicating a knowledge gap that might be filled with focused training. However, some respondents (*n* = 24) have a clear familiarity regarding cybersecurity threats.

**Awareness of bio-cybersecurity (Q20):** There is a varied level of awareness regarding bio-cybersecurity among users (*n* = 55); while most respondents were aware of the concept, a significant number were either unsure (*n* = 31) or unaware (*n* = 22) of it. It also indicates that although while bio-cybersecurity is well known, more individuals still need to gain knowledge about it and become more conscious.

#### Overall conclusion of user knowledge and awareness of cybersecurity

According to the analysis of user knowledge and awareness of cybersecurity in the context of NGS-technologies, all respondents indicated they are quite aware of the potential cybersecurity dangers associated with these technologies, indicating a generally high level of awareness associated with them. While most users either completely or partially grasp the relevance of securing genetic data, a significant portion do not have this important awareness, indicating a knowledge gap that can affect data security. Users’ familiarity with common cybersecurity threats varies considerably; many are only slightly knowledgeable, while a smaller percentage are either not familiar at all or very familiar. This suggests that customized training is necessary to guarantee a more widespread comprehension of the cybersecurity threats that can impact NGS data. Most respondents exhibited a basic understanding of the term “bio-cybersecurity,” yet many of them were either unsure or unaware of it. This heterogeneity points to a possible area for education and communication enhancement by indicating that although people grasp the concept, it has not yet been fully incorporated into the broader understanding of cybersecurity. Overall, there are major gaps in knowledge regarding the significance of data security, familiarity with threats, and awareness of bio-cybersecurity, despite a strong basic awareness of cybersecurity risks. By filling in these gaps, more enhanced and focused training programs could improve users’ cybersecurity strategy when handling NGS data and make sure they are more planned to secure sensitive genomic information from potential threats.

### Validation of hypotheses based on results

The links between the measured variables and the hypotheses are highlighted in this section. The results of this investigation support that the hypotheses by validating that the organizational maturity levels for cybersecurity profiles (*i.e*., robust, moderate, and emergent) are reflected in the findings.

#### Validation of H1

Results achieved from Q7 which highlights regarding the confidence in identifying cybersecurity threats, Q12 which targets cybersecurity breaches affecting NGS data and Cluster analysis clearly validate H1. A comparatively high average confidence level (mean of 72.24) in detecting cybersecurity threats (Q7) is reported by the results in ‘Practical recommendations by organizational profile’. It is concluded that this increased degree of confidence implies more advanced cybersecurity techniques. Furthermore, the statistical results from Q12 show that organizations with strong practices are more likely to update their policies in response to cyber incidents. Additionally, reduced insider risks are associated with this vigilant strategy. Cluster analysis further supports this finding by separating groups based on their cybersecurity culture. It is also observed in ‘Recommendations for organizations’ and ‘Practical recommendations by organizational profile’ that those organizations who prefer for regular trainings, robust cybersecurity policies and rigorous revisions to policies after a security breach exhibits fewer insider threats. Therefore, the conclusion achieved through these results organizations that follows robust cybersecurity practices will exhibit fewer insider threats, higher employee confidence in threat detection, and more frequent incident-driven policy updates.

#### Validation of H2

The statistical results related to awareness of cybersecurity risks (Q17), understanding the importance of securing genomic data (Q18), familiarity with common cybersecurity threats (Q19), and awareness of bio-cybersecurity (Q20) clearly validate H2. The results illustrate that organizations with moderate cybersecurity practices exhibit awareness levels that are neither as substantial as in robust organizations nor as low as in developing. This “middle-ground” performance corresponds to an intermediate threat awareness level. The moderate cluster further defined by this reactive strategy rather than proactive decisions. Therefore, the findings strongly validate H2, showing that organizations with moderate cybersecurity strategies depends on reactive measures after an incident, exhibit inconsistent policy enforcement, and exhibit intermediate levels of threat awareness.

#### Validation of H3

The statistical results achieved related to frequency of cybersecurity training (Q5), frequency of participation in cybersecurity training (Q6), confidence in identifying cybersecurity threats (Q7), detection of potential cybersecurity threat (Q10) and correlation analysis clearly validate H2. According to ‘Recommendations for organizations’, a substantial number of respondents from emergent organizations reported the conduction of minimal to no cybersecurity related trainings. One of the primary indicators of an emerging cybersecurity approach is this low level of training involvements. Additionally, from the cluster analysis performed in Section 5.6 it is evident that organizations with developing cybersecurity practices have lower confidence levels. Furthermore, the results from Q10 (poorest threat detection) reflects that fewer events of threat detection were identified in these emerging organizations. In addition to this, the overall average for Q7 (lowest employee confidence) is high across the study population in ‘Practical recommendations by organizational profile’. Additionally, the results of correlation analysis performed by using the Chi-Square Test indicate that greater susceptibility to insider threats is essentially correlated with minimal training, lower threat detection capabilities, and reduced confidence level which is directly validating H3.

### Scalability, broader applicability, limitations, and reproducibility

Our findings, although generated within the specific context of NGS, have broad applicability across various domains handling sensitive data, including healthcare, financial institutions, and governmental agencies. The recommendations, particularly those related to cybersecurity training effectiveness, insider threat mitigation, and organizational cybersecurity maturity profiling (robust, moderate, emergent), are structured in a manner that ensures adaptability and generalizability. Clearly defined maturity profiles allow other domains to benchmark their cybersecurity practices, identify gaps, and implement appropriate interventions. The role-specific cybersecurity training modules, incident-driven policy updates, and strategies to foster proactive cybersecurity cultures are universally relevant, promoting scalability and broader implementation across diverse organizational contexts.

Nevertheless, several limitations must be acknowledged. First, the study relied on a relatively modest sample size of 120 participants, potentially limiting the generalizability of findings. Participants originated from diverse geographic and organizational contexts, which might introduce variability affecting the consistency of results across different settings. Additionally, the voluntary nature of participation and reliance on self-reported data raise concerns about potential biases, including voluntary response and social desirability biases. Future research should address these limitations by including larger, geographically broader, and systematically sampled participant pools to validate findings more robustly.

To ensure reproducibility, comprehensive methodological transparency has been maintained. Detailed descriptions of the questionnaire design, participant selection criteria, ethical considerations, and statistical analyses—including descriptive statistics, frequency analysis, cross-tabulation, chi-square tests, and cluster analysis—are explicitly documented. All survey instruments and analytical frameworks are provided clearly, facilitating straightforward replication in similar or different contexts. The dataset generated from this study is openly accessible for academic research, subject to anonymization protocols that remove personal and organizational identifiers to ensure participant confidentiality and compliance with data protection regulations.

## Conclusion, recommendations and future work

### Conclusion

The study underscores the critical role of human factors and organizational behavior in mitigating insider threats within NGS environments. Despite advancements in cybersecurity technologies, organizations remain vulnerable due to gaps in training, inconsistent policy enforcement, and a lack of proactive security culture. Key findings reveal:

- **Training deficiencies:** Irregular and generic cybersecurity training leaves employees, especially early-career professionals, ill-prepared to identify and respond to NGS-specific threats.
- **Low threat detection confidence:** While awareness of general cybersecurity concepts is moderate, confidence in threat detection and mitigation remains inconsistent, particularly among students and non-IT staff.
- **Reactive policies:** Many organizations update policies only after incidents, highlighting a reactive rather than proactive approach to cybersecurity.
- **Organizational maturity disparities:** Cluster analysis identified three profiles—robust, moderate, and emergent—with stark differences in preparedness. Robust organizations, characterized by frequent training and cross-departmental communication, exhibit fewer insider threats.

These findings validate the study’s hypotheses, emphasizing that insider threats are exacerbated by insufficient training, poor engagement, and underfunded risk management. The convergence of these factors creates vulnerabilities that malicious or negligent insiders can exploit, jeopardizing sensitive genomic data.

### Recommendations for organizations

To address these challenges, organizations handling NGS data should adopt the following measures:

#### Implement role-specific cybersecurity training

- **Frequency:** Conducting quarterly (rather than annual or biannual) role-specific training sessions ensures that employees retain critical cybersecurity knowledge, stay updated on emerging threats, and reinforce best practices through repetition. Frequent training reduces the likelihood of human error—such as accidental data breaches or falling for phishing scams—by keeping cybersecurity top of mind.
- **Content:** Incorporating realistic exercises such as phishing drills and mock data breaches is critical for developing practical cybersecurity skills in NGS environments. Unlike passive training methods, these simulations actively engage employees in recognizing and responding to threats they may encounter in their daily workflows. For NGS specifically, simulations help staff identify vulnerabilities unique to genomic data handling, such as detecting manipulated sequencing files or recognizing social engineering attempts targeting lab personnel. These exercises also provide measurable metrics like phishing click-through rates and incident response times, enabling organizations to identify knowledge gaps and track improvement.
- **Metrics:** Track participation, post-training assessments, and incident reports to evaluate effectiveness. Monitoring training participation, assessment scores, and incident reports provides actionable insights into cybersecurity program effectiveness. Participation data identifies engagement gaps, while assessments reveal specific knowledge deficiencies among different roles (*e.g*., bioinformaticians *vs* lab technicians).

#### Foster a proactive security culture

- **Leadership engagement:** Designate “cybersecurity champions” in each department to promote accountability. Implementing department-level cybersecurity champions—typically senior staff like lead bioinformaticians or lab supervisors—creates a frontline defense against insider threats. These trained advocates serve three critical functions: (1) monitoring unusual activities in NGS workflows (*e.g*., unauthorized access to BAM files), (2) modeling secure practices like proper credential management, and (3) translating complex security protocols into department-specific guidance. In genomic research environments, champions are particularly effective at identifying subtle risks like sample mishandling or pipeline vulnerabilities that might escape centralized IT oversight.
- **Incentivization:** Reward employees who report vulnerabilities or complete advanced training (*e.g*., recognition, career advancement).
- **Communication:** Regularly discuss cybersecurity in meetings and use multiple channels (emails, dashboards) to reinforce policies.

#### Enhance incident response and policy updates

- **Drills:** Conduct biannual incident response drills (*e.g*., ransomware attacks on sequencing databases). Conducting semi-annual simulated attacks (*e.g*., ransomware targeting sequencing databases or unauthorized data exfiltration attempts) prepares teams for real cybersecurity crises. These drills test both technical responses like system isolation/recovery and human factors including communication chains and decision-making under pressure. The drills also reveal hidden vulnerabilities in unique genomic data pipelines while building cross-departmental coordination between bioinformaticians, lab staff, and IT security teams.
- **Feedback loops:** Use post-incident reviews to update policies and training programs promptly.

#### Allocate resources strategically

- **Budget reallocation:** Increase funding for preventive measures (*e.g*., monitoring tools, role-based training) beyond the current $\sim$8.2% allocated to insider threats.
- **Collaborations:** Partner with academic institutions or government agencies (*e.g*., NIH Cyberbiosecurity initiatives) to access shared resources.

#### Adopt clear and enforceable policies

- **Standardize protocols:** Mandate encryption for genomic data, multi-factor authentication for LIMS access, and strict access controls.
- **Visibility:** Integrate policies into onboarding and refresher sessions, with compliance tracked *via* centralized dashboards.

#### Leverage regulatory frameworks

- Align practices with standards like NIST Cybersecurity Framework, HIPAA, or GDPR to ensure compliance and global scalability (Khadam et al., 2023).

### Practical recommendations by organizational profile

Based on the organizational clustering into *robust*, *moderate*, and *emergent* cybersecurity maturity levels, we propose the following targeted strategies to help organizations improve their cybersecurity posture:

**Emergent organizations**

These organizations exhibit minimal cybersecurity training and weak internal communication. To improve, we recommend:

- Implementing baseline cybersecurity training programs tailored to NGS-specific risks.
- Establishing basic access control and incident reporting protocols.
- Appointing a designated cybersecurity liaison to bridge gaps between technical and operational teams.

**Moderate organizations**

These organizations show partial engagement with cybersecurity but lack consistency. For these, we suggest:

- Formalizing training frequency (*e.g*., quarterly sessions) with NGS-context relevance.
- Conducting incident-response simulations to reinforce preparedness and learning.
- Improving cross-departmental communication between lab personnel, IT staff, and leadership.

**Robust organizations**

**These organizations demonstrate strong cybersecurity maturity.**

To sustain and advance their practices, we recommend:

- Introducing continuous feedback loops (*e.g*., post-training surveys, risk reporting).
- Performing regular policy reviews and updates based on emerging threats.
- Expanding employee engagement initiatives, such as recognition for secure practices and advanced role-specific modules.

These tailored recommendations aim to guide organizations at different maturity levels toward more effective and sustainable cyber-biosecurity practices, particularly in the context of sensitive NGS environments.

### Future directions

- Extended dataset and organizations: Future research should expand the dataset to include more organizations across diverse geographical regions and sectors to improve generalizability of findings.
- Machine learning applications: Implement machine learning algorithms to analyze user behavior patterns and detect potential insider threats proactively. Techniques like anomaly detection and predictive modeling could identify suspicious activities before they escalate.
- Continuous improvement: Regularly assess training effectiveness and policy relevance through employee feedback and threat simulations.
- Longitudinal studies: Conduct longitudinal studies to evaluate the long-term impact of implemented cybersecurity measures on reducing insider threats.

By prioritizing these measures, organizations can transform their cybersecurity posture from reactive to resilient, safeguarding sensitive genomic data against insider threats while fostering a culture of shared responsibility.

## Additional Information and Declarations

### Data Availability

The following information was supplied regarding data availability:

The data and code are available at GitHub and Zenodo:

- https://github.com/EngrDarakhshanSyed/Cyber-Security-inNGS.

- Syed, D. (2025). Next Generation Sequencing Under Attack: Investigating Insider Threats and Organizational Behaviour [Data set]. Zenodo. https://doi.org/10.5281/zenodo.15277816.
